# Coumarin-1,2,3-Triazole Conjugates as Molecular Scaffolds for the Selective Induction of ROS-Driven Apoptosis in Cancer Cells in the Development of Pyruvate Kinase M2 Inhibitors

**DOI:** 10.1021/acsomega.5c08635

**Published:** 2025-12-09

**Authors:** Gabriel Ouverney, Amanda de Andrade Borges, Acácio Souza da Silva, Caroline Reis Santiago Paschoal, Paula Alvarez Abreu, Analice Gonçalves Rodrigues da Cruz, Mateus de Freitas Brito, Lucas Silva Abreu, Vitor Francisco Ferreira, Fernando de Carvalho da Silva, Bruno Kaufmann Robbs, Luana da Silva Magalhães Forezi

**Affiliations:** † Graduate Program in Morphological Sciences, Institute of Biomedical Sciences, 28125 Federal University of Rio de Janeiro, Fundão, Rio de Janeiro, RJ 21941-902, Brazil; ‡ Department of Organic Chemistry, Institute of Chemistry, 28110 Fluminense Federal University, Valonguinho Campus, Niterói, RJ 24020-150, Brazil; § Institute of Biodiversity and Sustainability (NUPEM), 314177 Federal University of Rio de Janeiro, Macaé, RJ 27965-045, Brazil; ∥ Graduate Program in Pharmaceutical Sciences, School of Pharmacy, Ilha Do Fundão, Rio de Janeiro, RJ 21941-599, Brazil; ⊥ Department of Pharmaceutical Technology, School of Pharmacy, Fluminense Federal University, Niterói, RJ 24020-141, Brazil; # Department of Basic Science, Nova Friburgo University Campus, Fluminense Federal University, Nova Friburgo, RJ 28625-650, Brazil

## Abstract

The increasing incidence of cancer and the emergence of drug resistance underscore the urgent need for new therapeutic options. This study aimed to synthesize and evaluate new coumarin–triazole hybrids for their cytotoxic selectivity and safety profile, providing insights into their potential as anticancer scaffolds. Twelve novel coumarin-based compounds were obtained in good yields (40–80%) and evaluated through *in vitro*, *in silico*, and *in vivo* assays. Among them, compound **7f** displayed the highest antiproliferative potency and selectivity, with selectivity indices (SI) of 4.61 for B16F-10, 3.38 for HCT116, and 2.98 for 4T1, and an average SI of 2.18 across SCC-4, SCC-9, and SCC-25 cell lines. Mechanistic assays indicated that the cytotoxic effect of **7f** is associated with oxidative stress-induced apoptosis, as evidenced by elevated ROS levels and activation of caspases 3/7. *In vivo* toxicity assessment in C57BL/6 mice revealed no significant changes in body weight, food intake, or macroscopic organ alterations at doses of up to 400 mg/kg, indicating a favorable safety profile. Additionally, *in vitro* biochemical evaluation and molecular docking suggested that **7f** interacts with Pyruvate Kinase M2 (PKM2) and inhibits its glycolytic activity in a dose-dependent manner. Overall, the findings identify compound **7f** as a selective and low-toxicity coumarin hybrid that exerts cytotoxic effects through ROS-mediated apoptosis, representing a promising lead structure for future optimization in anticancer drug development.

## Graphical abstract

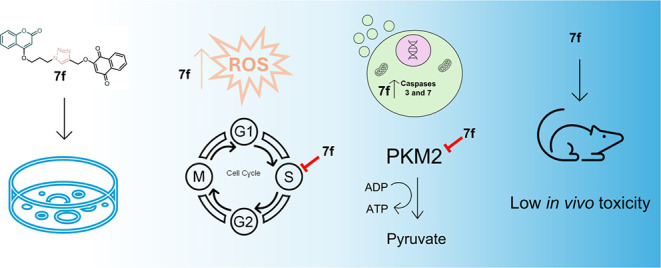

## 1 Introduction

Several substances with different pharmacophoric groups have attracted interest due to their synthetic versatility and relevant biological properties, especially those containing heterocyclic cores, which are present in over 70% of current drugs.^1^ In this context, the search for new chemical entities capable of modulating molecular targets associated with cancer, such as enzymes involved in the altered metabolism of tumor cells, has gained increasing prominence in the literature. The structural combination of bioactive scaffolds, such as coumarin, 1*H*-1,2,3-triazole and naphthoquinone, has emerged as a promising approach for identifying new drug candidates with optimized antitumor activity.^2–4^ These compounds exhibit a broad spectrum of biological activities, including bactericidal, antifungal, anti-inflammatory, and antiproliferative effects.^5–8^ The structural combination of coumarins with 1,2,3-triazoles has been studied as a promising strategy for developing new hybrid molecules with enhanced efficacy, lower toxicity, and multiple mechanisms of action against various diseases.^9^ These compounds interact with various pharmacological targets through noncovalent interactions, including hydrogen bonding, π–π stacking, and hydrophobic interactions.^10^ Coumarin, triazole, and naphthoquinone derivatives are reported to efficiently target several vital pathways in carcinogenesis, such as PI3K/AKT/mTOR, STAT3 transcription factor, vasculogenesis through inhibition of VEGF, repression of antiapoptotic proteins from BCL-2 family and activation of apoptotic cascade through effector caspases.^11,12^ In many cases, coumarin and naphthoquinone derivatives exert pro-oxidative effects by increasing the intracellular levels of reactive oxygen species (ROS). This accumulation is particularly detrimental to cancer cells, which often possess a saturated antioxidant system and a diminished ability to neutralize elevated ROS levels, unlike normal cells, which maintain more effective antioxidant defenses.^13^ In addition, derivatives of these classes of compounds, such as shikonin, are reported to have, among their mechanisms of action, targeting PKM2 activity, which leads to lower pyruvate production and reduced ATP quantification.^14^

One of the hallmarks of cancer, one of the most significant problems faced by most of the health systems worldwide, is a shift in cellular metabolism, prioritizing glycolysis and lactic fermentation over the electron transport chain—a phenomenon known as the Warburg Effect.^15^ A central player in this process is the M2 isoform of pyruvate kinase (PKM2), which is overexpressed in many tumors and may be associated with cancer resistance, tumor progression, and poor prognosis.^16–18^ PKM2 confers a metabolic advantage to cancer cells due to its reduced pyruvate kinase activity, which leads to the accumulation of upstream glycolytic intermediates. These metabolites can then be redirected into biosynthetic pathways, such as the pentose phosphate pathway and serine biosynthesis.^19^ The relevance of targeting PKM2 in therapy leading to inhibition of Warburg Effect-related cell events, such as diminished lactate production and GLUT-1 expression, ultimately resulting in decreased proliferation and cell viability, has already been well documented.^20^ For instance, apoptosis and regression of NSCLC tumors are directly related to lower cytosolic and nuclear PKM2 expression following treatment with a small-molecule inhibitor and shRNA.^21^ PKM2 is aberrantly upregulated in clinical samples from patients with oral squamous cell carcinoma (OSCC), where its elevated expression correlates with aggressive phenotypes and poor prognosis; conversely, pharmacological inhibition or genetic silencing of PKM2 in SCC-derived oral cancer cell lines markedly suppresses proliferation, migration, and metastatic potential.^22,23^ Our group recently demonstrated that a coumarin-1,2,3-triazole-naphthoquinone derivative strongly inhibits PKM2 enzymatic activity, thereby supporting the rationale that integrating these structural scaffolds in the present study could provide an effective strategy to counteract PKM2-driven oncogenic processes.^24^ Consequently, PKM2 emerges as a promising target in the development of anticancer therapeutics.

In this study, 12 new coumarin hybrids, comprising seven coumarin-triazole conjugates **7a**–**g** and five coumarin-triazole hybrids **7h**–**7l**, were synthesized from 4-hydroxycoumarin, representing pioneering examples of a novel class of potent cytotoxic compounds against oral squamous cell carcinoma (OSCC). This investigation explores the potential mechanism of action and evaluates the pharmacokinetic and toxicity profiles of the most promising derivative using a combination of *in silico*, *in vitro*, and *in vivo* approaches to assess its viability as a candidate for developing novel PKM2 inhibitors.

## 2 Results and Discussion

This study aimed to synthesize and evaluate new coumarin-1,2,3-triazole hybrids as potential anticancer agents targeting PKM2. Twelve novel compounds were prepared and characterized, followed by *in vitro* screening for cytotoxicity and selectivity. The most active derivative was further investigated through mechanistic assays, molecular docking, enzymatic inhibition, and *in vivo* toxicity studies to elucidate its mode of action and safety profile.

### 2.1 Chemistry

A series of coumarin-1,2,3-triazole conjugates was synthesized through a multistep procedure starting from 4-hydroxycoumarin (**1**). Intermediates of types **3a** and **3b** were obtained via the *O*-alkylation of 4-hydroxycoumarin (**1**) with dibromoethane or dibromopropane in the presence of potassium carbonate and dry DMF at 65 °C for 3 h, affording the compounds **3a** and **3b** in 86% and 87% yields, respectively. These intermediates were subsequently treated with sodium azide in DMSO, using potassium iodide as a catalyst at 70 °C, to give azido compounds **4a** (74%) and **4b** (72%). The resulting azides were then subjected to a CuAAC protocol with a series of presynthesized **6a**–**e** and commercially available alkynes **6f**–**h**, in a water/*tert*-butanol mixture, affording the desired compounds **7a**–**l**. These final compounds were obtained in yields ranging from 40% to 80%, calculated after purification by silica gel column chromatography using a gradient of ethyl acetate and hexane (Scheme 1).

**Scheme 1:**
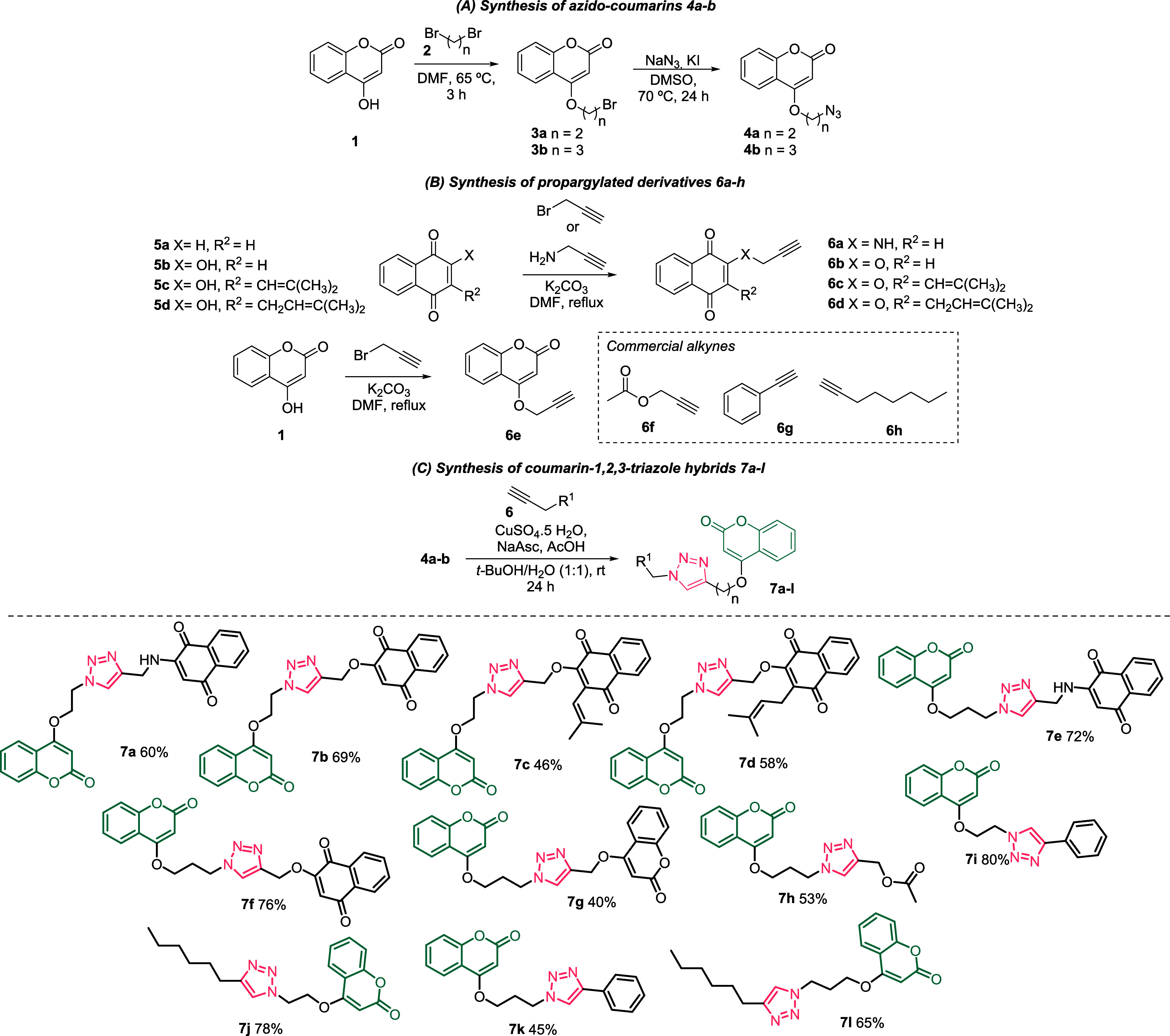
Synthesis of Coumarin-Based Derivatives **7a–l.**

### 2.2 Biological Assays

#### 2.2.1 Compounds Selectively Inhibit Cancer Cell Lines

Initially, 12 compounds **7a**–**l** were screened against the SCC-9 oral squamous cell carcinoma cell line using an MTT cell viability assay. Carboplatin, a DNA-alkylating agent commonly used in the clinic settings for the treatment of oral cancer, was employed as a positive control to benchmark the activity of the novel compounds.^25^ Cells treated with the vehicle (DMSO) alone served as the negative control in all experiments, ensuring that the observed effects could be exclusively attributable to the tested compounds. This assay enables the determination of compound potency, expressed as the IC_50_. All compounds were tested at a maximum concentration of 150 μM, above which they formed well-structured crystals; therefore, compounds that did not exhibit cytotoxicity were assigned an IC_50_ value of >150 μM. Among the 12 compounds, only **7c** and **7f** effectively induced cytotoxicity in the SCC-9 line, with **7f** having the greatest potency (IC_50_ = 12.29 μM) (Table 1). Compounds that did not exhibit cytotoxicity against SCC-9 were not tested in the remaining cell lines. Despite the structural similarity between some hybrids (e.g., **7b** and **7f**), the incorporation of a single methylene unit in the alkyl spacer appears to markedly influence cytotoxic potency. The modification in linker architecture among drug candidates can modulate molecular flexibility, lipophilicity, and the spatial orientation of key pharmacophoric moieties, thereby potentially enhancing target engagement and cellular uptake.^26,27^ Thus, this structural adjustment may not only facilitate improved membrane permeability of compound **7f** but also contribute to more favorable interactions with biological targets implicated in its cytotoxic activity.

**Table 1:** Compounds **7c** and **7f** Exert Cytotoxic Potential in Oral Cancer Cells^a^

	SCC-9	
Compounds	IC_50_ (μM)	SD
**7a**	>200	N.D.
**7b**	>200	N.D.
**7c**	28.95	0.03
**7d**	>150	N.D.
**7e**	>150	N.D.
**7f**	12.29	0.06
**7g**	>150	N.D.
**7h**	>200	N.D.
**7i**	>200	N.D.
**7j**	>200	N.D.
**7k**	N.D.	N.D.
**7l**	>150	N.D.
**Carboplatin**	150.40	0.07

a Determination of cytotoxicity of 12 compounds (**7a**–**7l**) is indicated by half-maximal concentration (IC_50_) against the SCC-9 cell line obtained from the MTT cell viability assay. Standard deviation is indicated as SD. Statistical data are representative of three different experiments. Not determined = N.D.

The initial screening was performed in SCC-9 cells because it is usually more sensitive to anticancer drugs. However, to restrict possible abnormalities in the behavior of a single cell line when compared with cancer cells in patients, it is important to consider other oral cancer cell lines as well as determine whether the effect is general or cancer-specific. Therefore, the cytotoxicity of selected substances was tested in two additional SCC tumor cell lines (SCC-4 and SCC-25; Table 2) and also on cancer cell lines from different origins (Table 3). SCC-9 and SCC-25 demonstrated greater resistance to **7c** and **7f,** whereas SCC-4 was the most sensitive cell line (Table 2). Squamous cell carcinoma lines exhibit distinct phenotypic characteristics, including differences in colony formation, proliferation rates, and cellular morphology.^28^ These lines also comprise heterogeneous populations, including CD44+ subpopulations that express proliferative genes, contributing to tumor aggressiveness and the acquisition of resistance, as well as mesenchymal-like cell subpopulations that exhibit intrinsic drug resistance.^29,30^ Another important distinction among these cell lines lies in their differential miRNA expression profiles. Studies have shown that differential expressions of specific miRNAs, such as miR-365, are associated with the resistant phenotype observed in SCC-9 and SCC-25, whereas SCC-4 displays a slower proliferation rate and greater sensitivity.^31^ Furthermore, miR-375 and miR-155 have been implicated in resistance mechanisms in SCC-9 and SCC-25 and are absent in SCC-4 cells.^32^ This may partially explain the differences in the activity of both compounds in the tested cancer cell lines.

**Table 2:** Coumarin Hybrid **7f** Selectively Inhibits Other Oral Cancer Cell Lines^a^

		**Cancer OSCC cells**									
	SCC-9			SCC-4			**SCC-25**			Primary Gingival Fibroblast	
Compounds	IC_50_ (μM)	SD	SI	IC_50_ (μM)	SD	SI	IC_50_ (μM)	SD	SI	IC_50_ (μM)	Average SI
**7c**	28.95	0.03	1.07	16.99	0.04	1.82	28.51	0.04	1.08	30.90	1.32
**7f**	12.29	0.06	1.85	7.93	0.08	2.87	11.09	0.03	2.05	22.77	2.26
**Carboplatin**	150.40	0.07	2.31	142.20	0.10	2.45	248.90	0.03	1.40	347.90	2.05

a Half-maximal inhibitory concentration (IC_50_) in other human OSCC is indicated. Human gingival fibroblast was used to calculate the selectivity index (SI). SD is the standard deviation. Statistical data are representative of three different experiments.

**Table 3:** Compound **7f** Selectively Inhibits Other Cancer Cell Lines^a^

	**7f**		
Cell Line	IC_50_ (μM)	SD	SI
**4T1**	7.63	0.02	2.98
**Hep-G2**	15.98	0.02	1.42
**HCT116**	6.72	0.02	3.38
**B16–F10**	4.93	0.04	4.93
**Primary Gingival Fibroblast**	22.77	0.04	-

a Half-maximal inhibitory concentration (IC_50_) in human tumor-derived hepatocellular carcinoma Hep-G2 and human colon carcinoma HCT116, altogether the inhibition toward murine tumor-derived cell lines from breast and melanoma 4T1 and B16–F10, respectively, is indicated. Human gingiva fibroblast was used to calculate the selectivity index (SI). SD = standard deviation. All statistical indicators represent results from at least three different experiments.

To further evaluate the efficacy of **7c** and **7f,** both compounds were subjected to an MTT cell viability assay using primary human gingival fibroblasts (HGF) as the normal cell model, under the same concentration conditions as in the cancer cells (Table 2). The inclusion of normal cells enables the assessment of a compound’s selectivity, a critical parameter in early-stage drug discovery. Table 2 presents the IC_50_ values of **7c** and **7f** in normal cells along with their average selectivity indices (SI).

Selectivity indices quantify the cytotoxic preference of compounds for cancerous over normal cells. Compounds exhibiting an SI value ≥ 2 are generally considered selective.^33,34^ Accordingly, after integrating both potency and selectivity data, **7f** (average SI of 2.26) demonstrated superior biological activity compared to the other compounds, exhibiting greater potency and selectivity comparable to that of carboplatin, a reference drug in oral cancer treatment.

Further screening of **7f** was conducted against additional cancer cell lines, including Hep-G2 (human hepatocellular carcinoma), HCT116 (human colon carcinoma), 4T1 (murine breast cancer), and B16–F10 (murine melanoma). Among these, B16–F10 (IC_50_ = 4.93 μM) was the most sensitive to **7f**, reflecting the highest selectivity index. Compound **7f** also demonstrated favorable selectivity in 4T1 and HCT116 cells, with SI values of 2.98 and 3.38, respectively. However, it did not exhibit selective inhibition in Hep-G2 cells (IC_50_ = 15.98 μM; SI = 1.42), as shown in Table 3. It will be interesting, in further studies, to analyze the high selectivity observed in B16–F10 and determine if it correlates with the putative targets proposed below.

The collected data indicates that **7f** is the most promising compound identified in this study for the development of effective antiproliferative agents, and it stands out as a leading candidate for subsequent *in vivo* evaluation. However, before animal studies are initiated, it is essential to verify their safety profile through additional *in vitro* toxicity assessments. Consequently, **7f** was subjected to a hemolysis assay to investigate its potential membrane-disrupting (surfactant-like) effects.

Hemolysis assay is a well-established tool in drug development, providing insights into compound selectivity and the risk of side effects.^35^ In this experiment, 1% Triton X-100 was used as a positive control due to its known hemolytic activity at concentrations above 0.01%.^36^ The level of hemolysis induced by **7f** was deemed acceptable (around 5%), especially considering that the compound was solubilized in DMSO, a solvent capable of inducing hemolysis on its own (Figure 1A). In contrast, neither carboplatin nor PBS affected the membrane integrity. Based on these results, the coumarin hybrid **7f** demonstrated a favorable safety margin and was selected for subsequent *in vivo* toxicological evaluation. To further characterize its safety profile, an acute toxicity study was performed in 12-week-old mice, with compound **7f** administered at three dose levels ranging from 100 to 400 mg/kg. The animals were monitored daily, and no signs of morbidity or mortality were detected (Table S1). Moreover, no significant changes were observed in body weight or food intake throughout the study duration (Figure 1B,C).

**Figure 1:**
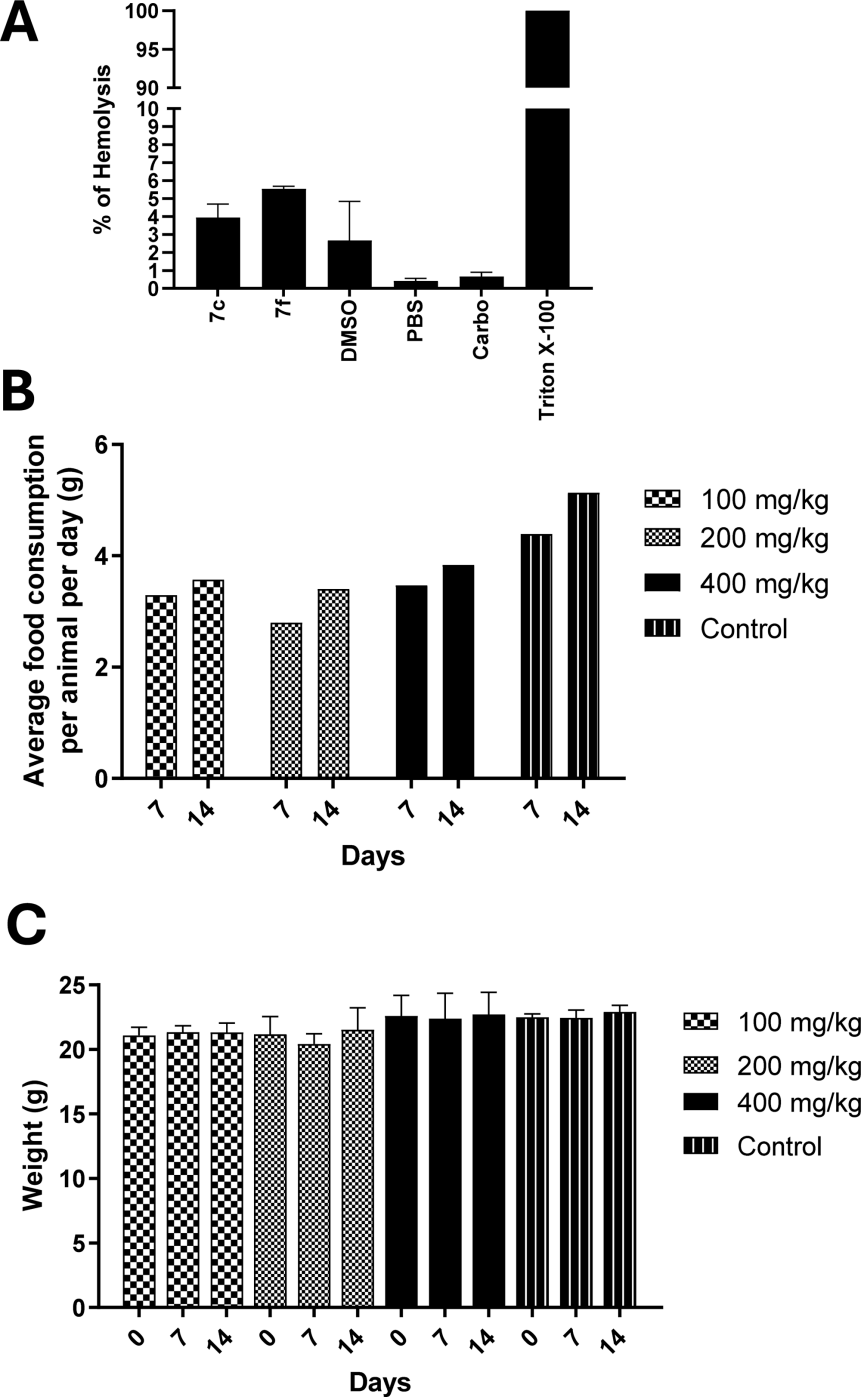
Tolerance of **7f** was demonstrated both *in vitro* and *in vivo*. (A) Hemolytic potential of **7f**. Graphical representation of the results from treatment of erythrocytes with **7f**, DMSO, PBS, carboplatin, and Triton X-100. (B,C) **7f** exerted low toxicity in C57BL/6 mice. Results from food consumption (B) and body weight (C) after 14 days from intraperitoneal administration of **7f** in different concentrations (100, 200, and 400 mg/kg) and Control (3% DMSO).

#### 2.2.2 Compound **7f** Induces Cellular Events Indicative of Apoptosis

To accurately investigate the potential cell death pathway induced by **7f** in cancer cells, video microscopy was initially employed to determine the timing of cell death and monitor the associated morphological changes. As shown in Figure 2A, SCC-9 cells treated with 2 × IC_50_ **7f** exhibited a pronounced inhibition of proliferation, in contrast to the untreated (DMSO) control group, which maintained active proliferation. Morphological alterations, including the disruption of intercellular junctions, membrane blebbing, and subsequent proliferation arrest, were observed after 6 h, with cytotoxic effects becoming more evident after 12 h.

**Figure 2:**
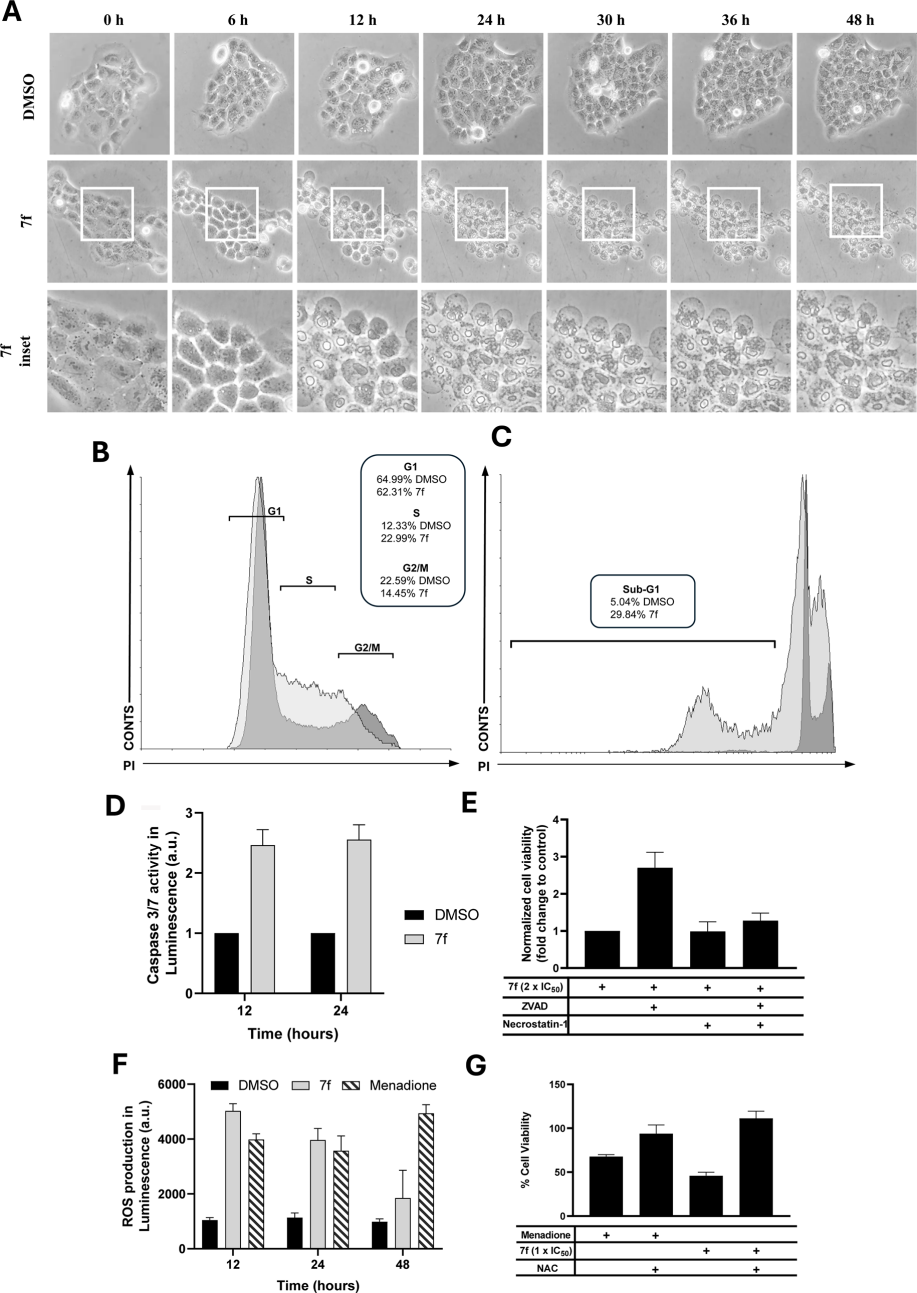
Cell death induced by **7f** suggests a process of ROS-induced apoptosis. (A) Morphological changes in SCC-9 cells treated with 2 × IC_50_ **7f** and nontreated (DMSO) are indicated throughout 48 h. Representative flow cytometry analysis in SCC-9 cells treated with 2 × IC_50_ **7f** (light gray) and nontreated (DMSO) (dark gray). Cells were treated for 24 h for cell cycle (B) and for 48 h for DNA fragmentation (sub-G1) analysis (C). (D) Effector caspases 3 and 7 activity were analyzed by luminometry in SCC-9 cells treated with 2 × IC_50_ of **7f** for 12 and 24 h. Luminescence was normalized by the DMSO group. (E) Cell viability assay using SCC-9 cells treated with **7f** alone or in combination with 20 μM z-VAD-fmk (ZVAD) and 20 μM necrostatin-1 (Nec-1) for 24 h. (F) Production of hydrogen peroxide (H_2_O_2_) is indicated as a luminescent sign for SCC-9 cells treated with **7f**, DMSO, and menadione, negative and positive controls, respectively. (G) Cell viability assay with SCC-9 cells treated with **7f** alone or in the presence of 10 mM *N*-acetyl-l-cysteine (NAC). Cells were treated with 1 × IC_50_ for 48 h. Menadione was the positive control. All results represent at least three independent experiments.

The administration of chemotherapeutic agents often affects the cell cycle, potentially inducing cell death. In particular, p53 activation and its downstream effector p21 inhibit cyclin-dependent kinases (CDKs), thereby disrupting cell cycle progression.^37^ SCC-9 cells treated with 2 × IC_50_ **7f** for 24 h exhibited S-phase arrest, which was approximately twice as frequent as in the control group (DMSO) (Figure 2B). This arrest is typically mediated by DNA damage, which inhibits CDK2-cyclin E and A complex formation, which plays a key role in the G1 and S-phase checkpoints.^38,39^ Notably, previous studies have demonstrated that a 2-fold increase in S-phase arrest correlates with a 3-fold increase in apoptosis incidence.^40^ Consistent with our findings, Radwan and coworkers reported that coumarin derivatives induced a 12% increase in S-phase arrest in Hep-G2 cells after 24 h, accompanied by phosphatidylserine externalization and late necrosis.^41^

In our study, DNA fragmentation (sub-G1) analysis revealed a 5-fold increase in the sub-G1 population of SCC-9 cells after 48 h of treatment with **7f** (Figure 2C). This is in line with previous reports on naphthoquinone derivatives, which similarly induced apoptosis, late necrosis, and increased sub-G1 populations.^42^ DNA fragmentation is also associated with at least a 2-fold increase in caspases 3 and 7 activity, further supporting the pro-apoptotic mechanism of action of **7f**.^43^

To further elucidate the mechanisms underlying cell death induced by **7f**, caspases 3 and 7 activity was assessed in SCC-9 cells treated with 2 × IC_50_ of the compound for 12 and 24 h. The luminescent signal detected in treated cells was approximately 2-fold higher than that in the nontreated (DMSO) control group (Figure 2D), indicating activation of effector caspases. Previous studies have demonstrated that a 2- to 3-fold increase in caspase-3/7 activity is typically associated with hallmark apoptotic events, including DNA fragmentation, chromatin condensation, and phosphatidylserine externalization.^43,44^ Therefore, the observed magnitude of caspase activation is consistent with the induction of apoptosis under the tested conditions. Additionally, the presence of caspase 3 excludes the possibility of pyroptosis in cells after **7f** treatment, although the morphology of dead cells may resemble this death pathway, as it was described that caspase 3 can cleave GSDMD, reduce pore formation, and inflammation.^45^

To further elucidate the mechanisms underlying **7f-**induced cytotoxicity, SCC-9 cells were exposed to selective inhibitors of apoptosis (z-VAD-fmk, ZVAD) and necroptosis (necrostatin-1), either alone or in combination. These two inhibitors were chosen based on the pro-apoptotic characteristics observed to date, despite the lack of membrane blebbing. Nonetheless, microscopy evidenced a significant release of intracellular contents, which could be a necroptotic marker and, therefore, warranted further investigation. The treatments were limited to 24 h to avoid cytotoxic effects associated with prolonged exposure, particularly linked to the pan-caspase inhibitor ZVAD.^46^ The mode of action of necrostatin-1 is based on the suppression of the pro-necroptotic protein RIP1 kinase.^47^ Cell death was significantly attenuated in cells pretreated with ZVAD, as evidenced by the increased cell viability in this group compared to **7f** treatment alone (Figure 2E). In contrast, pretreatment with Nec-1, either alone or in combination with ZVAD, failed to reverse the cytotoxic effect of **7f**, suggesting that necroptosis is unlikely to be the predominant mechanism underlying the observed cell death morphology.

Considering that oxidative stress is a well-established trigger of programmed cell death—particularly through caspase-3-dependent apoptotic pathways in cancer cells—we evaluated the pro-oxidant potential of compound 7f by quantifying intracellular hydrogen peroxide (H_2_O_2_) levels.^45^ The exposure of SCC-9 cells to **7f** resulted in a significant increase in ROS after 12 and 24 h (Figure 2F), higher than the positive control Menadione. The decline in ROS at the later time point of 48 h is explained by the advanced stage of cell death. The molecular structure of **7f** incorporates both naphthoquinone and coumarin moieties. While 1,4-naphthoquinone derivatives are characterized by exerting cytotoxicity via ROS generation, coumarin derivatives are typically associated with antioxidant properties.^48,49^ However, under specific redox conditions, coumarins can also participate in Fenton-type reactions, intensifying the oxidative stress.^50^ This duality may explain the observed oxidant behavior. Reactive oxygen species are addressed as promoters of mitochondrial permeabilization due to oxidation of the outer membrane, which in turn can initiate apoptosis through cytochrome c release, apoptosome assembly, and consequently, effector caspase 3 activity.^51^ Altogether, these findings suggest that the oxidant nature of **7f** is intimately linked to its apoptotic potential through caspase 3 and 7 activation. To evaluate the contribution of oxidative stress, SCC-9 cells were pretreated with the antioxidant *N*-acetyl-l-cysteine (NAC) for 2 h and then maintained with NAC for 48 h of exposure to **7f** (Figure 2G). NAC protected cell death **7f**-induced cytotoxicity, as well as menadione, thereby confirming the oxidative nature of **7f**’s mechanism of action.

The complexity in cancer therapy with the contribution of multiresistant tumors demands the creation of hybrid molecules that could surpass resistance by acting on central targets, thus optimizing chemotherapeutic approaches.^52,53^ Therefore, this work aimed to identify the potential molecular targets of the most promising coumarin hybrid. Previous research conducted by our group demonstrated that coumarin-based compounds act as potent inhibitors of pyruvate kinase M2 (PKM2).^24^ Considering these findings and the notable biological activity of **7f**, this study focused on investigating whether its mechanism of action involves interference with the PKM2 signaling pathway. As an initial approach, the interaction between **7f** and PKM2 was assessed *in silico* through a molecular docking analysis.

### 2.3 PKM2 Inhibition Mediated by **7f**

#### 2.3.1 Molecular Docking Studies

All chemical structures of the docked compounds are shown in Supplementary Figure S37 for clarification. The interaction of **7f** with PKM2 was investigated alongside PKM2 inhibitors shikonin and lapachol for comparative purposes (Figure 3).^14,54^ Hybrid **7f** exhibited a binding mode similar to that of lapachol, binding at the entrance of the active site and overlapping with the adenine portion of the cocrystallized ATP. Comparatively, **7f** appeared to have a binding pose similar to ATP, with the pentane ring positioned closer to the ribose of the reference moiety (Figure 3A–E). This compound is more favorable than shikonin *redocking* (−8.0 kcal·mol^–1^) (Figure 3B), lapachol (−7.7 kcal·mol^–1^) (Figure 3C), and ATP (−9.4 kcal·mol^–1^) (Figure 3D) when compared with the binding energy of **7f** with PKM2 that was −9.9 kcal·mol^–1^ (Figure 3E) in the rabbit enzyme (PDB entry 1a49). Compound **7f** maintained an interaction with the residue Asn75 via van der Waals forces, which appears to be important for the enzyme inhibition. The Lys207 residue was also conserved in the interaction with all compounds through hydrogen bonding, except for lapachol due to its size. However, lapachol maintained this interaction in a weaker form through van der Waals forces. Thus, due to its binding mode comparable to ATP in the crystallized PKM2-ATP complex, the similarity of the interaction with the inhibitor lapachol, and its binding affinity compared to the other reference compounds, the analysis suggests that PKM2 is a likely target involved in the anticancer activity presented by **7f**. In addition to PKM2, other proteins were identified as putative targets of **7f** in a target search through structural similarity, such as carbonic anhydrase XII (CAXII) and topoisomerase IIα (Supplementary Figures S38 and S39).

**Figure 3:**
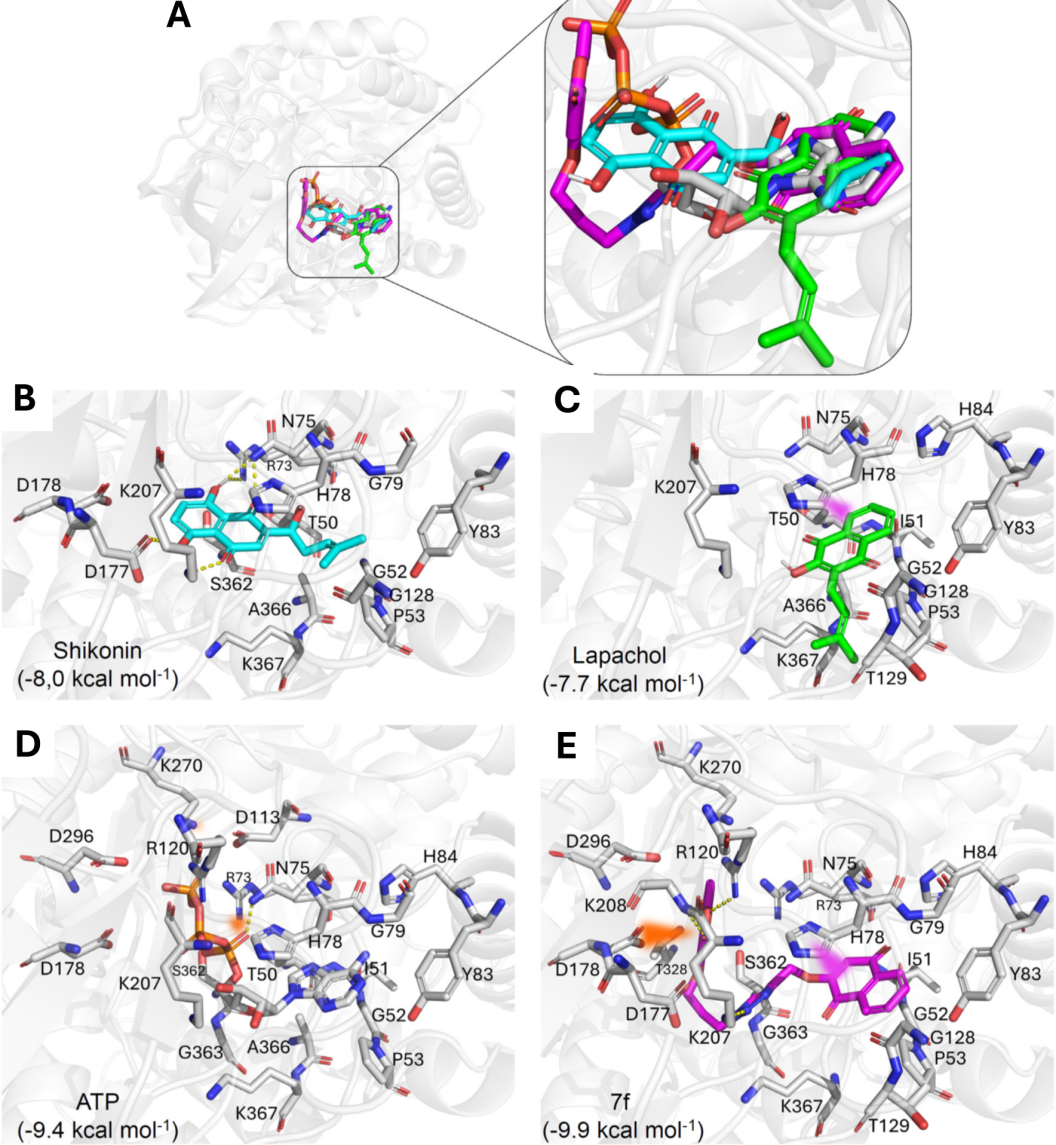
Binding mode in tyrosine protein kinase 2 (PKM2) of the compounds (A) cocrystallized ATP (white), lapachol (green), shikonin (cyan), and **7f** (magenta). Interaction between the compounds (B) shikonin (−8 kcal mol^–1^), (C) lapachol (−7.7 kcal mol^–1^), (D) cocrystallized ATP (redocking −9.4 kcal mol^–1^) and (E) **7f** (−9.9 kcal mol^–1^) with the residues of PKM2. In yellow dashed lines are the hydrogen bonding interactions, in pink triangles are the π–π interactions, and in orange are the salt bridge, π–cation, or π–anion interactions.

Supported by the bioinformatics results, we proceeded to investigate the *in vitro* inhibition of PKM2 induced by **7f**. The initial step involved an enzymatic reaction in which a PKM2 solution was incubated with varying concentrations of the coumarin hybrid. The reaction was carried out for 30 min, sufficient for complete substrate depletion. As shown in Figure 4, compound **7f** inhibited PKM2 activity in a dose-dependent manner, with an IC_50_ value of 55.13 μM (raw data at Supplementary Table S2).

**Figure 4:**
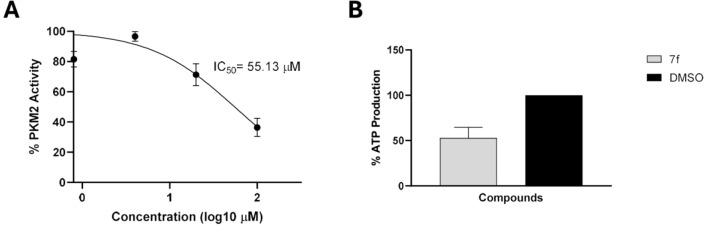
PKM2 activity was inhibited in the presence of **7f**. (A) Nonlinear regression curve indicating the inhibition of PKM2 activity. PK activity was assessed with an LDH-coupled assay. The concentrations vary from 0.8 μM to 100 μM. (B) ATPase activity of PKM2 was reduced upon 1 × IC_50_ **7f** treatment in comparison to vehicle (DMSO) and nontreated PKM2. PKM2 reaction without the presence of phosphoenolpyruvate (PEP) was used as a negative control.

This analysis relied on an LDH-coupled system that measures NADH oxidation into NAD+, a product of lactate dehydrogenase activity.^19^ To further validate the direct inhibition of PKM2 by **7f,** ATP production resulting from phosphoenolpyruvate (PEP) conversion into pyruvate was quantified.^19^ To confirm the direct inhibition of PKM2, the production of ATP was measured in the enzymatic reaction in the presence of 1 × IC_50_ of **7f** (55.13 μM), which inhibited approximately 50% of the ATP production. These results suggest direct interaction between **7f** and PKM2, although they do not confirm whether the inhibition takes place at the enzyme’s site.

Given the complexity of cancer and the challenge of overcoming multidrug resistance, the hybridization of compounds may enhance anticancer activity and develop target-specific drug candidates.^53^ In this context and with the results presented in this work, coumarin derivatives represent a strategic approach for enhancing anticancer activity through PKM2 inhibition.

Indeed, PKM2 inhibition by naphthoquinone derivatives has been shown to affect cellular metabolism, leading to S-phase arrest in the cell cycle, apoptosis via caspase 3 activation, and late necrosis in ovarian cancer cells, effects mirrored by **7f** in oral cancer cells.^55^ Moreover, PKM2 inhibition has been explored as an adjuvant mechanism, as demonstrated by shikonin-mediated necroptosis restoring cisplatin sensitivity in bladder cancer via RIP3 induction.^16^

Compared to compound **6e**, a previously reported PKM2 inhibitor from our group, **7f** showed slightly reduced inhibitory potency but significantly greater *in vivo* tolerability, making it a promising scaffold for the development of safer PKM2-targeted therapies.^24^

As demonstrated in the cytotoxicity assay using SCC-9 cells, the greater potency of compound 7f compared to its activity in the PKM2 cell-free assay suggests that PKM2 inhibition is unlikely to be the primary mechanism of action. Rather, the pronounced cytotoxicity may be more attributable to the intense ROS production induced by this coumarin derivative. Notably, previously reported imidazopyri­(mi)­dine-flanked tetrazole derivatives with favorable PKM2 inhibitory activity also induce apoptosis primarily through ROS-mediated mechanisms.^56^ These findings support the notion that while oxidant compounds such as 7f may impact PKM2-related pathways, their dominant cytotoxic effect is largely driven by oxidative stress.

## 3 Conclusion

The data collected throughout this study converge to identify compound **7f** as the most promising molecule among those evaluated. From the initial screening against cancer cell lines, **7f** consistently exhibited the lowest IC_50_ values, highlighting it as the most potent compound among the 12 tested. In addition to its strong antiproliferative potency, **7f** demonstrated a satisfactory selectivity higher than 2 against oral cancer cells (2.26) as well as in 4T1 (2.98), HCT116 (3.38), and B16F10 (4.61). Furthermore, the low toxicity profile of **7f** was supported by its lack of significant hemolytic activity and *in vivo* studies in mice, where the compound was well tolerated at doses up to 400 mg/kg. These results, in combination with the prediction of positive drug-likeness properties, indicate that **7f** may be a favorable candidate for further *in vivo* testing of therapeutic potential. Among the tested compounds, **7f** provided the clearest evidence of apoptosis induction, promoting the generation of reactive oxygen species (ROS), increased effector caspase activity, and DNA fragmentation, events typically associated with apoptosis-triggered cell death. A noteworthy aspect of this study was the effort to better understand the potential involvement of coumarin derivatives in modulating the PKM2 enzymatic activity. As demonstrated, **7f** can directly inhibit this pathway, suggesting an additional mechanism through which it may exert anticancer effects. However, further studies are required to validate this interaction, particularly through the assessment of glucose uptake and lactate production in treated cells, including appropriate controls such as shikonin, to enable a direct comparison of **7f** with established PKM2 inhibitors. Nevertheless, the induction of ROS appears to be the predominant effect of **7f**, as evidenced by the reversal of its cytotoxicity upon treatment with the antioxidant *N*-acetyl-l-cysteine, thereby positioning PKM2 modulation as a putative secondary mechanism. These approaches could support the use of **7f** as a scaffold for the development of PKM2 inhibitors, through either structural modifications or synthesis of new analogues. In conclusion, this study identified a promising molecule, compound **7f**, that demonstrated potent, selective, and low *in vivo* toxicity, with a seemingly well-defined mechanism of action involving apoptosis and ROS production as well as notable potential to inhibit PKM2.

## 4 Materials and Methods

### 4.1 Chemistry

The melting ranges of the substances were obtained in a Thermo Scientific 9100 apparatus or Fischer–Jones apparatus (Melting Point Apparatus) series 50200082. Spectra in the infrared region were obtained using a PerkinElmer FT-IR spectrometer, models 1600 Series and Spectrum One, dual beam, on anhydrous potassium bromide pellets. Absorption values were expressed in wavenumber, using the reciprocal centimeter (cm^–1^) as the unit. Nuclear magnetic resonance spectra were acquired on a Varian Unity spectrometer operating at 500.00 MHz (for ^1^H NMR) and 125.0 MHz (for ^13^C NMR), using CDCl_3_ or DMSO-d_6_ as the solvent, as specified in each case. NMR spectra were typically obtained at room temperature. Chemical shift values (δ) were reported in parts per million (ppm) relative to tetramethylsilane (TMS) used as an internal standard, and coupling constants (*J*) were reported in Hertz (Hz). Signal areas were obtained by an electronic integration. The multiplicities of absorption bands in the ^1^H NMR spectrum have been described as follows: s (singlet), d (doublet), t (triplet), q (quartet), quint (quintet), m (multiplet), dd (double doublet), td (triplet of doublets), and ddd (doublet of doublet of doublets). High-resolution mass spectra were obtained on a MICROMASS Q-TOF mass spectrometer (Waters) with electrospray ionization (ESI). All solvents and reagents used were purchased from commercial sources, such as Sigma-Aldrich Brazil (São Paulo, SP, Brazil). According to the needs in the methodologies adopted, the solvents were treated, distilled, and dried according to the processes described in the literature. Chromatography monitoring of all reactions was performed by thin-layer chromatography (TLC), using silicagel 60 F_254_ chromatosheets, 0.2 mm thick (ref Merck 5554). Most substances were purified by column chromatography using silicagel 60 (70–230 mesh) supplied by Merck.

#### 4.1.1 General Procedures for Synthesis of *O*-Alkylated Coumarins **3a** and **3b**

Compound **1** (2.00 g, 12.3 mmol) was dissolved in 10 mL of DMF, followed by the addition of anhydrous K_2_CO_3_ (3.40 g, 24.7 mmol). Then dibromoethane or dibromopropane (24.7 mmol) was added dropwise to the stirred reaction mixture. The stirring continued for 3 h at 65 °C. The reaction mixture was extracted with CH_2_Cl_2_, the organic extracts were washed with water, dried over Na_2_SO_4_, and filtered, and the solvent was removed in vacuo. The residue was recrystallized from EtOH, leading to the *O*-alkylated products **3a** and **3b** in 86% and 87% yield, respectively.^57^

#### 4.1.2 General Procedures for Synthesis of Azidocoumarins **4a** and **4b**

A mixture of coumarin **3** or **4** (1 g, 1 mmol) and NaN_3_ (3 mmol) in 10 mL of dry DMF was reacted. Then a minimum amount of KI was added. The reaction mixture was worked up to afford coumarin **4a** and **4b** as an off-white powdery solid and was used without further purification.^58^

#### 4.1.3 General Procedures for Synthesis of Nor-Lapachol (**5c**)

In a round-bottom flask, lawsone (**5b**, 10 mmol), β-alanine (2.8 mmol), and toluene (100 mL) as a solvent were added. Separately, a mixture of isobutyraldehyde (20 mmol) and acetic acid (0.15 mL) in toluene (10 mL) was prepared and then introduced into the flask. The resulting mixture was refluxed for 1.5 h using a Dean–Stark apparatus coupled to a condenser in an open system. When the reaction was completed, the mixture was cooled to room temperature and extracted with a saturated sodium carbonate solution until the aqueous layer lost its purple color. The aqueous layer was then acidified to pH 2–3 using an aqueous o-phosphoric acid solution of 85% w/v. After 24 h, the precipitate was filtered under vacuum, washed with ice-cold water, and dried in a desiccator to yield an orange solid identified as compound **5c**. Orange solid (2.19 g, 96%).

#### 4.1.4 General Procedures for Synthesis of 2-(Prop-2-ynylamino) naphthalene-1,4-dione (**6a**)

1,4-Naphthoquinone (**5a**, 1 mmol) was dissolved in ethanol (10 mL), and propargyl amine (2 mmol) was added dropwise at room temperature. The reaction progress was monitored by TLC and considered complete after 1.5 h. Subsequently, the resulting mixture was filtered and washed with cold ethanol and diethyl ether, yielding a light brown solid, identified as compound **6a**. This product was used in the next step without further purification. Light brown solid (1.69 g, 80%).

#### 4.1.5 General Procedures for **6b**

In a 125 mL round-bottomed flask, 1 mmol of lawsone (**5b**), 4 mmol of potassium carbonate, and 15 mL of DMF were added. The reaction mixture was left under magnetic stirring at room temperature for 20 min. Then, 4 mmol of propargyl bromide was added via a syringe and was stirred for 24 h at 60 °C. After this time, the reaction mixture was filtered and washed with chloroform, and the filtrate was evaporated under reduced pressure. The product was purified by column chromatography using flash silica gel, with a mixture of ethyl acetate and hexane as eluent in a concentration gradient. Compound **6b** was obtained as a fine yellow solid in 52% yield.

#### 4.1.6 General Procedures for Synthesis of Compounds **6c** and **6d**

In a 50 mL round-bottom flask containing **5b** or **5d** (1 mmol) and potassium carbonate (1.5 mmol), 5 mL of dry acetone was added, and the mixture was stirred for 15 min at room temperature. Propargyl bromide (1.5 mmol) was added dropwise. The reaction mixture was stirred for 72 h, during which it was necessary to add 1.5 mmol of propargylamine every 24 h until complete consumption of the starting material. After that, the reaction mixture was extracted with dichloromethane (30 mL) and washed with water (3 × 25 mL). The organic layer was dried over anhydrous sodium sulfate, filtered off, and the solvent was removed by reduced-pressure evaporation. The resultant residue was purified by flash column chromatography using a hexane/ethyl acetate gradient to afford products **6c** or **6d**, respectively. **6c**, orange solid (0.873 g, 82%).

#### 4.1.7 General Procedures for Synthesis of 4-(Prop-2-yn-1-yloxy)-2H-chromen-2-one **6e**

In a 125 mL round-bottom flask, 1.2 mmol of 4-hydroxycoumarin (**1**), 2.4 mmol of potassium carbonate, and 7 mL of DMF were added. The reaction mixture was left under magnetic stirring at room temperature for 20 min. Then, 1.8 mmol of propargyl bromide was added and stirred for 24 h at 120 °C. After this time, the reaction mixture was filtered and washed with ethyl acetate, and the filtrate was evaporated under reduced pressure. The product was purified by column chromatography using flash silica gel, employing chloroform as an eluent. Compound **6e** was obtained as a fine white solid in 80% yield.^24^

#### 4.1.8 General Procedures for Synthesis of Coumarin-1,2,3-Triazole Conjugates **7a–l**

In a 125 mL round-bottom flask, the azido compounds **4a** or **4b** (300 mg, 1 equiv) and alkyne (**6a**–**h**) (1 equiv), copper sulfate pentahydrate (4 equiv), sodium ascorbate (4 equiv), and 30 mL of a water/*tert*-butanol (1:1) mixture were added and then left under magnetic stirring for 24 h. Following this, the mixture was extracted with dichloromethane (3 × 20 mL), and the combined organic phases were dried over anhydrous sodium sulfate. Finally, the solvent was removed under reduced pressure, and the crude product **7a**–**l** was then purified by chromatography on a silica gel column, with a mixture of ethyl acetate and hexane as the eluent in a concentration gradient.

##### 4.1.8.1 2-(((1-(2-((2-Oxo-2H-chromen-4-yl)­oxy)­ethyl)-1H-1,2,3-triazol-4-yl)­methyl)­amino) naphthalene-1,4-dione (**7a**)

**Orange solid**; yield 60%; mp >215 °C (dec); ^1^H NMR (500 MHz, DMSO-d_6_) δ 8.20 (s, 1H), 7.95 (dd, *J* = 7.8, 1.3 Hz, 1H), 7.89 (dd, *J* = 7.8, 1.3 Hz, 1H), 7.83–7.74 (m, 2H), 7.70 (td, *J* = 7.6, 1.4 Hz, 1H), 7.61 (dd, *J* = 8.0, 1.6 Hz, 1H), 7.56–7.48 (m, 1H), 7.27 (d, *J* = 8.3 Hz, 1H), 7.18 (t, *J* = 7.0 Hz, 1H), 5.89 (s, 1H), 5.75 (s, 1H), 4.89 (t, *J* = 4.9 Hz, 2H), 4.62 (t, *J* = 4.9 Hz, 2H), 4.50 (d, *J* = 6.3 Hz, 2H); ^13^C NMR (125 MHz, DMSO-d_6_) δ 181.32, 181.24, 164.00, 161.12, 152.50, 147.93, 143.10, 134.53, 132.82, 132.41, 131.97, 130.18, 125.59, 125.14, 123.79, 123.61, 122.41, 116.09, 114.72, 100.43, 90.86, 67.57, 48.23, 37.29; HRMS (ESI) *m*/*z* [M + Na]^+^ calcd for C_24_H_18_N_4_NaO_5_^+^ 465.1169, found 465.1161. Purity (HPLC-DAD, 254 nm): 98.8%.

##### 4.1.8.2 2-((1-(2-((2-Oxo-2H-chromen-4-yl)­oxy)­ethyl)-1H-1,2,3-triazol-4-yl)­methoxy) naphthalene-1,4-dione (**7b**)

**Off-white solid**; yield 69%; mp >220 °C (dec); ^1^H NMR (500 MHz, DMSO-d_6_) δ 8.48 (s, 1H), 7.96 (t, *J* = 7.8 Hz, 2H), 7.88–7.76 (m, 2H), 7.69 (dd, *J* = 7.9, 1.3 Hz, 1H), 7.58 (t, *J* = 7.3 Hz, 1H), 7.32 (d, *J* = 8.3 Hz, 1H), 7.28 (t, *J* = 7.6 Hz, 1H), 6.58 (s, 1H), 5.93 (s, 1H), 5.29 (s, 2H), 4.96 (t, *J* = 4.8 Hz, 2H), 4.67 (t, *J* = 4.9 Hz, 2H); ^13^C NMR (125 MHz, DMSO-d_6_) δ 184.75, 179.91, 161.71, 159.45, 153.31, 141.75, 134.84, 133.98, 133.12, 132.09, 131.41, 126.49, 126.24, 125.98, 124.52, 123.22, 116.77, 115.55, 111.62, 91.64, 68.14, 63.00, 49.06; HRMS (ESI) *m*/*z* [M + Na]^+^ calcd for C_24_H_17_N_3_NaO_6_^+^ 466.1009, found 466.1003. Purity (HPLC-DAD, 254 nm): 95.4%.

##### 4.1.8.3 2-(2-Methylprop-1-en-1-yl)-3-((1-(2-((2-oxo-2H-chromen-4-yl)­oxy)­ethyl)-1H-1,2,3-triazol-4-yl)­methoxy)­naphthalene-1,4-dione (**7c**)

**Yellow solid**; yield 46%; mp >130 °C (dec); ^1^H NMR (500 MHz, DMSO-d_6_) δ 8.30 (s, 1H), 7.99–7.94 (m, 1H), 7.92–7.87 (m, 1H), 7.84–7.76 (m, 2H), 7.68 (dd, *J* = 7.9, 1.6 Hz, 1H), 7.62 (ddd, *J* = 8.7, 7.3, 1.6 Hz, 1H), 7.35 (dd, *J* = 8.4, 1.1 Hz, 1H), 7.28 (td, *J* = 7.6, 1.1 Hz, 1H), 5.92 (s, 1H), 5.74 (s, 1H), 5.36 (s, 2H), 4.92 (t, *J* = 4.9 Hz, 2H), 4.61 (t, *J* = 5.0 Hz, 2H), 1.77 (d, *J* = 1.5 Hz, 3H), 1.45 (d, *J* = 1.3 Hz, 3H); ^13^C NMR (125 MHz, DMSO-d_6_) δ 184.23, 180.87, 164.06, 161.15, 154.98, 152.58, 142.76, 141.60, 133.84, 133.42, 132.57, 131.36, 131.04, 130.56, 125.56, 125.51, 124.85, 123.89, 122.55, 116.18, 114.77, 114.55, 90.87, 67.66, 64.95, 48.26, 25.67, 20.98; HRMS (ESI) *m*/*z* [M + H]^+^ calcd for C_26_H_24_N_3_O_6_^+^ 498.1659, found 498.1662. *It was not possible to determine the purity of the sample by HPLC, as the compound did not show adequate solubility in the solvents commonly used for the analysis (acetonitrile and methanol). The sample precipitated during injection, resulting in partial blockage of the chromatographic column and compromising the performance of the assay.*

##### 4.1.8.4 2-(3-Methylbut-2-en-1-yl)-3-((1-(2-((2-oxo-2H-chromen-4-yl)­oxy)­ethyl)-1H-1,2,3-triazol-4-yl)­methoxy)­naphthalene-1,4-dione (**7d**)

**Yellow solid**; yield 58%; mp 177–179 °C; ^1^H NMR (500 MHz, DMSO-d_6_) δ 8.39 (s, 1H), 8.03–7.95 (m, 1H), 7.94–7.85 (m, 1H), 7.81–7.79 (m, 2H), 7.67 (d, *J* = 7.9 Hz, 1H), 7.61 (t, *J* = 8.0 Hz, 1H), 7.35 (d, *J* = 8.3 Hz, 1H), 7.26 (t, *J* = 7.6 Hz, 1H), 5.91 (s, 1H), 5.48 (s, 2H), 4.93 (t, *J* = 4.9 Hz, 2H), 4.86 (t, *J* = 7.5 Hz, 1H), 4.61 (t, *J* = 5.0 Hz, 2H), 2.96 (d, *J* = 7.3 Hz, 2H), 1.50 (s, 6H); ^13^C NMR (125 MHz, DMSO-d_6_) δ 184.25, 180.95, 164.05, 161.13, 155.83, 152.58, 142.67, 134.24, 133.88, 133.47, 132.55, 132.22, 131.17, 130.94, 125.63, 125.47, 125.07, 123.84, 122.51, 119.71, 116.15, 114.75, 90.83, 67.66, 65.33, 48.28, 25.07, 22.40, 17.27; HRMS (ESI) *m*/*z* [M + Na]^+^ calcd for C_29_H_25_N_3_NaO_6_^+^ 534.1635, found 534.1627. Purity (HPLC-DAD, 254 nm): 93.2%.

##### 4.1.8.5 2-(((1-(3-((2-Oxo-2H-chromen-4-yl)­oxy)­propyl)-1H-1,2,3-triazol-4-yl)­methyl)­amino) naphthalene-1,4-dione (**7e**)

**Orange solid**; yield 72%; mp 173–175 °C; ^1^H NMR (500 MHz, DMSO-d_6_) δ 8.11 (s, 1H), 7.97 (d, *J* = 7.6 Hz, 1H), 7.91 (d, *J* = 7.6 Hz, 1H), 7.80 (t, *J* = 7.5 Hz, 1H), 7.77–7.68 (m, 3H), 7.61 (t, *J* = 7.8 Hz, 1H), 7.33 (d, *J* = 8.2 Hz, 2H), 5.82 (s, 1H), 5.76 (s, 1H), 4.59 (t, *J* = 6.8 Hz, 2H), 4.46 (d, *J* = 6.2 Hz, 2H), 4.24 (t, *J* = 5.7 Hz, 2H), 2.40 (quint, *J* = 6.5 Hz, 2H); ^13^C NMR (125 MHz, DMSO-d_6_) δ 181.98, 181.95, 165.19, 161.94, 153.24, 148.65, 143.55, 135.23, 133.52, 133.12, 132.67, 130.87, 126.28, 125.82, 124.48, 123.80, 123.44, 116.79, 115.64, 101.08, 91.09, 67.19, 47.10, 38.00, 29.24; HRMS (ESI) *m*/*z* [M + H]^+^ calcd for C_25_H_21_N_4_O_5_^+^ 457.1506, found 457.1516. Purity (HPLC-DAD, 254 nm): 95.7%.

##### 4.1.8.6 2-((1-(3-((2-Oxo-2H-chromen-4-yl)­oxy)­propyl)-1H-1,2,3-triazol-4-yl)­methoxy)­naphthalene-1,4-dione (**7f**)

**Light brown solid**; yield 76%; mp >170 °C (dec); ^1^H NMR (500 MHz, DMSO-d_6_) δ 8.38 (s, 1H), 7.97 (t, *J* = 8.4 Hz, 2H), 7.89–7.78 (m, 2H), 7.75 (d, *J* = 8.1 Hz, 1H), 7.62 (t, *J* = 7.8 Hz, 1H), 7.35 (dd, *J* = 8.3, 5.5 Hz, 2H), 6.59 (s, 1H), 5.85 (s, 1H), 5.25 (s, 2H), 4.66 (t, *J* = 6.9 Hz, 2H), 4.28 (t, *J* = 5.8 Hz, 2H), 2.45 (quint, *J* = 6.4 Hz, 2H); ^13^C NMR (125 MHz, DMSO-d_6_) δ 184.19, 179.23, 164.53, 161.27, 158.74, 152.57, 140.77, 134.23, 133.37, 132.45, 131.32, 130.65, 125.85, 125.32, 125.25, 123.84, 122.80, 116.11, 114.97, 110.79, 90.45, 66.55, 62.27, 46.58, 28.57; HRMS (ESI) *m*/*z* [M + Na]^+^ calcd for C_25_H_19_N_3_NaO_6_^+^ 480.1166, found 480.1148. Purity (HPLC-DAD, 254 nm): 97.2%.

##### 4.1.8.7 4-(3-(4-(((2-Oxo-2H-chromen-4-yl)­oxy)­methyl)-1H-1,2,3-triazol-1-yl)­propoxy)-2H-chromen-2-one (**7g**)

**Green solid**; yield 40%; mp >168 °C (dec); ^1^H NMR (500 MHz, DMSO-d_6_) δ 8.43 (s, 1H), 7.75 (d, *J* = 7.8 Hz, 1H), 7.69 (d, *J* = 7.8 Hz, 1H), 7.63 (q, *J* = 8.5 Hz, 2H), 7.38–7.31 (m, 4H), 6.12 (s, 1H), 5.86 (s, 1H), 5.41 (s, 2H), 4.67 (t, *J* = 6.8 Hz, 2H), 4.29 (t, *J* = 5.7 Hz, 2H), 2.46 (q, *J* = 6.4 Hz, 2H); ^13^C NMR (125 MHz, DMSO-d_6_) δ 164.52, 164.12, 161.26, 161.20, 152.58, 152.54, 132.49, 132.43, 125.09, 123.86, 123.77, 122.75, 122.58, 116.17, 116.09, 114.93, 114.91, 91.08, 90.39, 66.58, 62.70, 46.64, 28.49; HRMS (ESI) *m*/*z* [M + H]^+^ calcd for C_24_H_20_N_3_O_6_^+^ 446.1347, found 446.1344. Purity (HPLC-DAD, 254 nm): 98.9%.

##### 4.1.8.8 (1-(3-((2-Oxo-2H-chromen-4-yl)­oxy)­propyl)-1H-1,2,3-triazol-4-yl)­methyl acetate (**7h**)

**White solid**; yield 53%; mp 137–139 °C; ^1^H NMR (500 MHz, DMSO-d_6_) δ 8.19 (s, 1H), 7.73 (d, *J* = 7.9 Hz, 1H), 7.64 (t, *J* = 7.9 Hz, 1H), 7.45–7.19 (m, 2H), 5.84 (s, 1H), 5.09 (s, 2H), 4.62 (t, *J* = 6.9 Hz, 2H), 4.26 (t, *J* = 5.8 Hz, 2H), 2.43 (p, *J* = 6.4 Hz, 2H), 2.00 (s, 3H); ^13^C NMR (125 MHz, DMSO-d_6_) δ 170.47, 165.21, 161.98, 153.26, 142.53, 133.14, 125.23, 124.48, 123.47, 116.79, 115.64, 91.08, 67.26, 57.61, 47.17, 29.21, 20.98; HRMS (ESI) *m*/*z* [M + Na]^+^ calcd for C_17_H_17_N_3_NaO_5_^+^ 366.1060, found 366.1064. Purity (HPLC-DAD, 254 nm): 98.1%.

##### 4.1.8.9 4-(2-(4-Phenyl-1H-1,2,3-triazol-1-yl)­ethoxy)-2H-chromen-2-one (**7i**)

**Off-white solid**; yield 80%; mp >200 °C (dec); ^1^H NMR (500 MHz, DMSO-d_6_) δ 8.69 (s, 1H), 7.83 (d, *J* = 6.8 Hz, 2H), 7.76 (dd, *J* = 7.9, 1.6 Hz, 1H), 7.62 (ddd, *J* = 8.6, 7.3, 1.6 Hz, 1H), 7.44 (t, *J* = 7.7 Hz, 2H), 7.38–7.29 (m, 3H), 5.97 (s, 1H), 4.96 (t, *J* = 5.0 Hz, 2H), 4.71 (t, *J* = 5.0 Hz, 2H); ^13^C NMR (125 MHz, DMSO-d_6_) δ 164.11, 161.16, 152.60, 146.31, 132.58, 130.56, 128.67, 127.65, 125.01, 123.95, 122.60, 121.79, 116.20, 114.84, 90.97, 67.56, 48.41; HRMS (ESI) *m*/*z* [M + H]^+^ calcd for C_19_H_16_N_3_O_3_^+^ 334.1186, found 334.1191. Purity (HPLC-DAD, 254 nm): 98.8%.

##### 4.1.8.10 4-(2-(4-Hexyl-1H-1,2,3-triazol-1-yl)­ethoxy)-2H-chromen-2-one (**7j**)

**White solid**; yield 78%; mp 167–169 °C; ^1^H NMR (500 MHz, DMSO-d_6_) δ 7.96 (s, 1H), 7.71 (dd, *J* = 8.0, 1.6 Hz, 1H), 7.68–7.58 (m, 1H), 7.36 (d, *J* = 8.3 Hz, 1H), 7.33 (t, *J* = 7.6 Hz, 1H), 5.92 (s, 1H), 4.84 (t, *J* = 5.0 Hz, 2H), 4.62 (t, *J* = 5.1 Hz, 2H), 2.60 (t, *J* = 7.5 Hz, 2H), 1.55 (p, *J* = 7.3 Hz, 2H), 1.31–1.09 (m, 7H), 0.91–0.76 (m, 3H); ^13^C NMR (125 MHz, DMSO-d_6_) δ 164.10, 161.15, 152.61, 146.85, 132.58, 123.88, 122.57, 122.23, 116.20, 114.84, 90.86, 67.69, 47.99, 30.72, 28.66, 27.86, 24.75, 21.69, 13.57; HRMS (ESI) *m*/*z* [M + H]^+^ calcd for C_19_H_24_N_3_O_3_^+^ 342.1812, found 342.1820. Purity (HPLC-DAD, 254 nm): 99.9%.

##### 4.1.8.11 4-(3-(4-Phenyl-1H-1,2,3-triazol-1-yl)­propoxy)-2H-chromen-2-one (**7k**)

**White solid**; yield 45%; mp 148–150 °C; ^1^H NMR (500 MHz, DMSO-d_6_) δ 8.64 (s, 1H), 7.82 (d, *J* = 7.6 Hz, 3H), 7.72 (d, *J* = 7.9 Hz, 1H), 7.62 (t, *J* = 8.0 Hz, 1H), 7.44 (t, *J* = 7.6 Hz, 3H), 7.39–7.29 (m, 3H), 7.22 (t, *J* = 7.6 Hz, 1H), 5.89 (s, 1H), 4.66 (t, *J* = 6.8 Hz, 2H), 4.31 (t, *J* = 5.8 Hz, 2H), 2.48 (quint, *J* = 6.3 Hz, 2H); ^13^C NMR (125 MHz, DMSO-d_6_) δ 164.58, 161.31, 152.57, 146.30, 132.43, 130.69, 128.60, 127.55, 124.98, 123.75, 122.78, 121.30, 116.10, 114.97, 90.44, 66.74, 46.72, 28.54; HRMS (ESI) *m*/*z* [M + Na]^+^ calcd for C_20_H_17_N_3_NaO_3_^+^ 370.1162, found 370.1163. Purity (HPLC-DAD, 254 nm): 98.5%.

##### 4.1.8.12 4-(3-(4-Hexyl-1H-1,2,3-triazol-1-yl)­propoxy)-2H-chromen-2-one (**7l**)

**Gray solid**; yield 65%; mp 107–110 °C; ^1^H NMR (500 MHz, DMSO-d_6_) δ 7.80 (s, 1H), 7.70 (d, *J* = 7.7 Hz, 1H), 7.64 (t, *J* = 7.8 Hz, 1H), 7.35–7.32 (m, 2H), 5.77 (s, 1H), 4.53 (t, *J* = 6.7 Hz, 2H), 4.22 (t, *J* = 5.7 Hz, 2H), 2.54 (t, *J* = 7.5 Hz, 2H), 2.39 (q, *J* = 6.2 Hz, 2H), 1.51 (q, *J* = 7.2 Hz, 3H), 1.26–1.20 (m, 6H), 0.82 (t, *J* = 6.7 Hz, 3H); ^13^C NMR (125 MHz, DMSO-d_6_) δ 164.50, 161.15, 152.56, 146.83, 132.30, 132.28, 123.66, 122.68, 121.45, 115.96, 114.98, 90.24, 66.56, 46.12, 30.58, 28.41, 27.83, 24.71, 21.52, 13.36; HRMS (ESI) *m*/*z* [M + H]^+^ calcd for C_20_H_26_N_3_O_3_^+^ 356.1969, found 356.1961. Purity (HPLC-DAD, 254 nm): 99.6%.

### 4.2 Biological Assays

#### 4.2.1 Cells and reagents

Human cancer cell lines SCC-4, SCC-9, and SCC-25 cells, derived from a human oral tongue SCC (squamous cell carcinoma), hepatocellular carcinoma Hep-G2, human colon carcinoma HCT116 cell, and murine tumor-derived cell lines from breast and melanoma (4T1 and B16–F10) were obtained from the American Type Culture Collection (ATCC) (ATCC numbers: CRL-1624, CRL-1629, CRL-1628, HB-8065, CCL-247, CRL-2539, and CRL-6475, respectively). Primary normal human gingival fibroblasts (HGF) were obtained from ATCC (PCS-201-018). SCCs were maintained in 1:1 DMEM/F12 (Dulbecco’s modified Eagle medium and Ham’s F12 medium; Gibco (Thermo Fisher, Waltham, MA, USA) supplemented with 10% (v/v) FBS (fetal bovine serum; Invitrogen, Thermo Fisher, Waltham, MA, USA) and 400 ng/mL hydrocortisone (Sigma-Aldrich Co., St. Louis, MO, USA), while other cell lines were maintained in DMEM supplemented with 10% (v/v) FBS. Cells were grown in a humidified environment containing 5% CO_2_ at 37 °C. For all biological experiments, tested compounds and control menadione were solubilized in 100% DMSO (Sigma-Aldrich Co., St. Louis, MO, USA) to a final concentration of 10 mM. The chemotherapeutic agent carboplatin stock was commercially acquired (Fauldcarbo; Libbs Farmacêutica, São Paulo, SP, Brazil) and was used as a standard anticancer compound.

#### 4.2.2 Cell Viability Assay

The viability of SCC cell lines and primary human fibroblast cells was evaluated using the MTT assay to determine half maximal inhibitory concentration (IC_50_).^59^ Briefly, cells were grown in triplicate in 96-well plates (5 × 10^3^ cells/well) until confluence. Then the medium was removed, fresh medium was added, and the cells were returned to the incubator in the presence of different compounds. DMSO at the same concentrations was used as a 100% cell viability control. After 48 h, cells were incubated with 5 mg/mL MTT reagent (3-(4,5-dimethyl-2-thiazolyl)-2,5-diphenyl-2-H-tetrazolium bromide) (Sigma-Aldrich Co., St. Louis, MO, USA) for 3.5 h. After that, formazan crystals were dissolved in MTT solvent solution (DMSO/methanol 1:1 v/v), and the absorbance at 560 nm was evaluated using an EPOCH microplate spectrophotometer (BioTek Instruments, Winooski, VT, USA) with the background absorbance at 670 nm subtracted. Each of the 12 compounds (**7a**–**7l**) was tested at six different concentrations, ranging from 6.25 to 200 μM in cancer and normal cell lines. Control (carboplatin) was tested at six different concentrations ranging from 25 μM to 800 μM in cancer cells and from 50 μM to 1600 μM in normal cells.

#### 4.2.3 Hemolysis Assay

A hemolysis assay to evaluate surfactant effects of tested substances was performed using human blood approved by the Research Ethics Committee of Universidade Federal Fluminense (CAAE: 43134721.4.0000.5626). Erythrocytes are isolated upon three repeated centrifugation cycles at 1500 rpm for 15 min. Between each cycle of centrifugation, supernatant was removed, and a pH 7.5 PBS (phosphate-buffered saline) solution supplemented with 10 mM glucose was added to remove lysed erythrocytes. The number of cells was determined in an Automatic Cell Counter (Thermo Fisher, Waltham, MA, USA). Erythrocytes were then plated in 96-well plates at a concentration of 4 × 10^8^ cells/well in triplicates, and 10 μL of compounds were added at a final concentration of 300 μM in PBS with glucose (final volume 100 μL). Ten μL of PBS was used as a negative control, and 10 μL of PBS with 0.1% Triton X-100 was used as a positive control. Data reading was performed with EPOCH (BioTek Instruments, Winooski, VT, USA) at an absorbance of 540 nm.

#### 4.2.4 Acute Toxicity Assay *In Vivo*

To determine the tolerated doses of the most selective compound *in vivo*, the acute toxicity study for compound **7f** was performed in 12-week-old C57BL/6 female mice via intraperitoneal injection and was approved by the University Animal Ethics Board under registration number 2699110419. All experiments were performed in accordance with the Brazilian guidelines and regulations. Dosing and analysis were performed according to 423 OECD guidelines and reviewed by ref. 60. An experimental approach was conducted using experimental groups (*n* = 3) in which each group received a single dose of **7f** (100, 200, or 400 mg/kg). Higher doses were determined based on the tolerance observed in the previous dosing. Control group animals received only 3% DMSO in PBS vehicle. Animals were examined every day, twice a day, for mortality and morbidity for 14 days, and all animals were sacrificed by cervical dislocation. The animals’ body weight and average food consumption were measured every 7 days.

#### 4.2.5 Videomicroscopy by Time-Lapse

The acquisition of images from the morphological changes in the cells treated with the most selective compound was conducted by creating a time-lapse video recording using an adapted Leica DMi1 (Leica Microsystems, Wetzlar, Germany) microscope. SCC-9 cells were plated in 35 mm culture dishes and then treated after 24 h with 2 × IC_50_ of **7f** (24.58 μM) or DMSO (vehicle) for a total of 48 h. Cells were maintained in a controlled chamber containing 5% CO_2_ and 37 °C. Images were captured every minute and integrated using Framelapse software (Neximo Laboratories, Uttar Pradesh, India).

#### 4.2.6 Cell Cycle and Sub-G1 Analysis

The interference of **7f** in the cell cycle (G1, M, and G2/M) and DNA fragmentation (sub-G1) was assessed by flow cytometry analysis using a Guava Muse Cell Analyzer (Cytek Biosciences, Fremont, CA, USA). DNA staining was conducted using a pH 7.6 propidium iodide buffer composed of 3.4 mM Trizma Hydrochloride (Sigma-Aldrich Co., St. Louis, MO, USA), 10 mM NaCl (Sigma-Aldrich Co., St. Louis, MO, USA), 0.1% (v/v) NP40 (Sigma-Aldrich Co., St. Louis, MO, USA), 700 U/L RNase, and 0.075 mM propidium iodide (Sigma-Aldrich Co., St. Louis, MO, USA). An amount of 1 × 10^5^ and 5 × 10^5^ SCC-9 cells were seeded in six-well plates for the treatment with the negative control (DMSO) and **7f**, respectively. And then, 24 h later, the supernatant was removed, and cells were treated with 2 × IC_50_ of **7f** or DMSO for 24 and 48 h. Cells were then trypsinized and stained with propidium iodide buffer. DNA content was analyzed by collecting 10,000 events in the aforementioned cytometer. Data were analyzed using Flowing Software (Turku Biosciences, Turku, Finland).

#### 4.2.7 Detection of Effector Caspases and Violet Crystal Cell Viability Assay

Analysis of activation of caspases 3 and 7 was assessed by using the Caspase-Glo 3/7 Assay Kit (Promega Corporation, Madison, WI, USA; G8090). The reagent provided by the kit causes cell lysis; therefore, parallelly, a cell viability assay using violet crystal was performed to normalize the detection of active caspases by viable cells. Caspases 3 and 7 procedures consisted of seeding 5 × 10^3^ SCC-9 cells in a 96-well plate. The next day, cells were treated with 2 × IC_50_ of the indicated compounds for 12 and 24 h. A plate with wells without cells was used as a control. Cells and cell-free wells remained in treatment at times of interest. Then, caspase activity was detected according to the manufacturer’s instructions. Results were obtained using a TD 20/20 luminometer (Turner Designs, Sunnyvale, CA, USA). For the wells that were used exclusively for cell viability evaluation, 5 × 10^3^ SCC-9 cells were seeded in a 96-well plate and treated the next day with 2 × IC_50_ of the indicated compounds for 12 and 24 h. Then the medium was removed, and the wells were gently washed with PBS. PBS was removed, and cells were fixed with 100% alcohol that was removed after 10 min. Cells were stained with 0.05% violet crystal for 10 min. Wells were gently washed with deionized water, and 100 μM methanol was added to each well, followed by agitation of the plate for 10 min. Absorbance of wells was read in an EPOCH (BioTek Instruments, Winooski, VT, USA) at an absorbance of 595 nm.

#### 4.2.8 Detection of H_2_O_2_ Detection of HO Production

The production of reactive oxygen species (ROS) was assessed by the detection of hydrogen peroxide (H_2_O_2_) using the ROS-Glo H_2_O_2_ assay kit (Promega Corporation, Madison, WI, USA; G8820). To carry out the experiment, 1 × 10^4^ SCC-9 cells were plated per well in a 96-well plate, and after 24 h of incubation, the medium was removed, and the wells were treated with 2 × IC_50_ of compound **7f** or with DMSO for the times of 12, 24, and 48 h. A plate with wells without cells was used as a control. Cells and cell-free wells remained in treatment at times of interest, and 2 h before completion of the times, well volumes were removed to reduce to 40 μL per well. Then the positive control of menadione (Sigma-Aldrich Co., St. Louis, MO, USA), a polycyclic aromatic ketone based on 1,4-naphthoquinone, was used to treat its respective well, and H_2_O_2_ substrate from the kit was added for all treatments, followed by the incubation of the plate for the remaining 2 h. Then the detection solution was added, and the plates remained at room temperature for 20 min, following the reading in the TD 20/20 luminometer (Turner Designs, Sunnyvale, CA, USA). The reading of bioluminescence detected is indicated as arbitrary units (a.u.). Additionally, the cell viability was assessed through pretreatment of cells with 10 mM *N*-acetyl-l-cysteine (NAC) (Sigma-Aldrich Co., St. Louis, MO, USA) for 2 h. And then cells were treated with 1 × IC_50_ of **7f** and 20 μM of menadione solutions containing NAC for 48 h. At the end of the time, the MTT cell viability assay was performed.

#### 4.2.9 Cytotoxicity Assay (Cell Death Inhibition)

This investigation consists of pretreating cells with different death inhibitors. For this assay, day SCC-9 cells were seeded in a 96-well plate, and the next day, they were pretreated for 2 h with 20 μM z-VAD-fmk (ZVAD) (Promega Corporation, Madison, WI, USA) and 20 μM necrostatin-1 (Nec-1) (Sigma-Aldrich Co., St. Louis, MO, USA) alone or in combination. After the 2 h, cells were treated with a 2 × IC_50_ of **7f** solution containing or not containing the respective inhibitors. The plate was incubated for 24 h, and the result was determined through an MTT cell viability assay. The absorbance was measured with an EPOCH (BioTek Instruments, Winooski, VT, USA) spectrometer at 560 nm.

#### 4.2.10 Pyruvate Kinase M2 Activity

LDH-coupled Pyruvate Kinase M2 (PKM2) activity assay was performed according to published protocols.^61^ For the assay, a pH 7.5 buffer solution composed of 50 mM Tris, 100 mM KCl, and 10 mM MgCl_2_ was prepared, to which 30 ng of human recombinant PKM2 (Sigma-Aldrich Co., St. Louis, MO, USA), 0.6 mM adenosine diphosphate (ADP), 0.5 mM phosphoenolpyruvate (PEP), 180 μM NADH, 10 μM fructose 1,6-bisphosphate (FBP), and 8 units of LDH were added. PEP was added only right before measurement to start the reaction. The change in absorbance at 340 nm due to oxidation of NADH by LDH was measured from 0 to 30 min using an EPOCH microplate spectrophotometer (BioTek Instruments, Winooski, VT, USA). The activity of PK was defined as % in relation to the indicated control.

#### 4.2.11 Quantification of ATP Production from PKM2 Reaction

The measurement of PKM2 activity through ATP production was conducted to confirm whether the enzymatic inhibition observed in the previous assay was indeed due to PKM2. The master mix was prepared as indicated above. In a microtube containing the master mix prepared in the presence of 1 × IC_50_ **7f**, the control received the addition of 0.5 mM PEP for 3 min. Afterward, CellTiter-Glo reagent (Promega, Madison, WI, USA) was added as indicated by the manufacturer’s instructions, and the samples were kept shielded from light for 2 min. After the specified time, the sample was taken for reading in a Turner Designs TD 2020 luminometer (Turner Designs, Sunnyvale, CA, USA).

### 4.3 Statistical Analysis

Half-maximal inhibitory concentration (IC_50_) values were obtained from the MTT assay of at least three independent replicates through nonlinear regression analysis using GraphPad Prism 5.0 software (Intuitive Software for Science, San Diego, CA, USA). Data were presented as means ± standard deviation (SD). A log dose–response curve (inhibitor vs response) using the least-squares method was used to determine the IC_50_ and SD from the data. The selectivity index (SI) was calculated as the ratio between the IC_50_ in normal cells and the IC_50_ in cancer cells, obtained by dividing the IC_50_ of normal cells by the IC_50_ of cancer cells.

### 4.4 Molecular Docking Studies

Molecular docking was performed using **7f** as a molecular target. Initially, the ligand was built, optimized, and electrostatic charges were calculated using Spartan’10 software (Wavefunction Inc., CA, USA). A conformational analysis was performed using the MMFF force field, and the lowest-energy conformer was optimized using the semiempirical PM3 method. Subsequently, an energy calculation was conducted using the Hartree–Fock method with the 6-31G* basis set (HF/6-31G*), and Mulliken charges were applied. The naphthoquinones shikonin and lapachol were subjected to the same protocol to obtain their structures and were used for comparative purposes. For molecular docking studies, Autodock Tools 1.5.7 and Autodock Vina 1.1.2.^62^ Solvents and artifacts present in the proteins were removed; polar hydrogens and Gasteiger charges were added; and the proteins were kept rigid. Ligands were kept flexible, and their torsional bonds were automatically defined using Autodock Tools version 1.5.7. The docking procedures employed in this study were previously reported and validated by our group.^24,42,63^ The binding pose with the lowest energy was visually inspected, and its interactions were analyzed using Discovery Studio Visualizer 2019 (Dassault Systèmes BIOVIA, San Diego, 2019) and PyMOL v 2.5.0 (The PyMOL Molecular Graphics System, version 2.5.0, Schrödinger, LLC, New York, NY, USA).

## Supplementary Material



## Data Availability

Data will be available upon request.

## Notes

^1^H NMR spectrum (DMSO-d_6_) (Figures S1, S4, S7, S10, S13, S16, S19, S22, S25, S28, S31, and S34); ^13^C NMR spectrum (DMSO-d_6_) (Figures S2, S5, S8, S11, S14, S17, S20, S23, S26, S29, S32, and S35); HRMS spectrum (Figures S3, S6, S9, S12, S15, S18, S21, S24, S27, S30, S33, and S36); the monitoring of the animals indicated low toxicity (Table S1); chemical structures of the compounds analyzed (Figure S37); binding mode of compounds (Figures S38 and S39); raw data used for IC_50_ calculation (Table S2); predicted safety profile (Table S3) (PDF)
