# Supplementary material for: Coumarin-1,2,3-Triazole Conjugates as Molecular Scaffolds for the Selective Induction of ROS-Driven Apoptosis in Cancer Cells in the Development of Pyruvate Kinase M2 Inhibitors
Source: ACS Omega. 2025 Dec 9;10(50):61899–914. doi: 10.1021/acsomega.5c08635 (PMC12750388; doi:10.1021/acsomega.5c08635)
Supplement: Supplementary file 1 [file ao5c08635_si_001.pdf]

SUPPLEMENTARY INFORMATION

Coumarin–1,2,3-Triazole Conjugates as Molecular Scaffolds for the Selective Induction of ROS-Driven Apoptosis in Cancer Cells in Development of Pyruvate Kinase M2 Inhibitors

Gabriel Ouverney,[a#] Amanda de Andrade Borges,[b#] Acácio Souza da Silva,[b] Caroline Reis Santiago Paschoal,[c,d] Paula Alvarez Abreu,[c] Analice Gonçalves Rodrigues da Cruz,[b] Mateus de Freitas Brito,[b] Lucas Silva Abreu,[b] Vitor Francisco Ferreira,[e] Fernando de Carvalho da Silva,[b] Bruno Kaufmann Robbs,[f,*] and Luana da Silva Magalhães Forezi[b,*]

[a]Graduate Program in Morphological Sciences, Institute of Biomedical Sciences, Federal University of Rio de Janeiro, Fundão, Rio de Janeiro 21941-902, RJ, Brazil.

[b]Department of Organic Chemistry, Institute of Chemistry, Valonguinho Campus, Fluminense Federal University, Niterói 24020-150, RJ, Brazil.

[c]Institute of Biodiversity and Sustainability (NUPEM), Federal University of Rio de Janeiro, Macaé 27965-045, RJ, Brazil.

[d]Graduate Program in Pharmaceutical Sciences, School of Pharmacy, Ilha do Fundão, Rio de Janeiro 21941-599, RJ, Brazil.

[e] Department of Pharmaceutical Technology, School of Pharmacy, Fluminense Federal University, Niterói 24020-141, RJ, Brazil.

[f]Department of Basic Science, Nova Friburgo University Campus, Fluminense Federal University, Nova Friburgo 28625-650, RJ, Brazil.

*Correspondent authors: luanaforezi@id.uff.br, brunokr@id.uff.br

#These authors contributed equally to this work.

^{1}H NMR spectrum, ^{13}C NMR spectrum and MS spectrum of intermediary and final compounds.

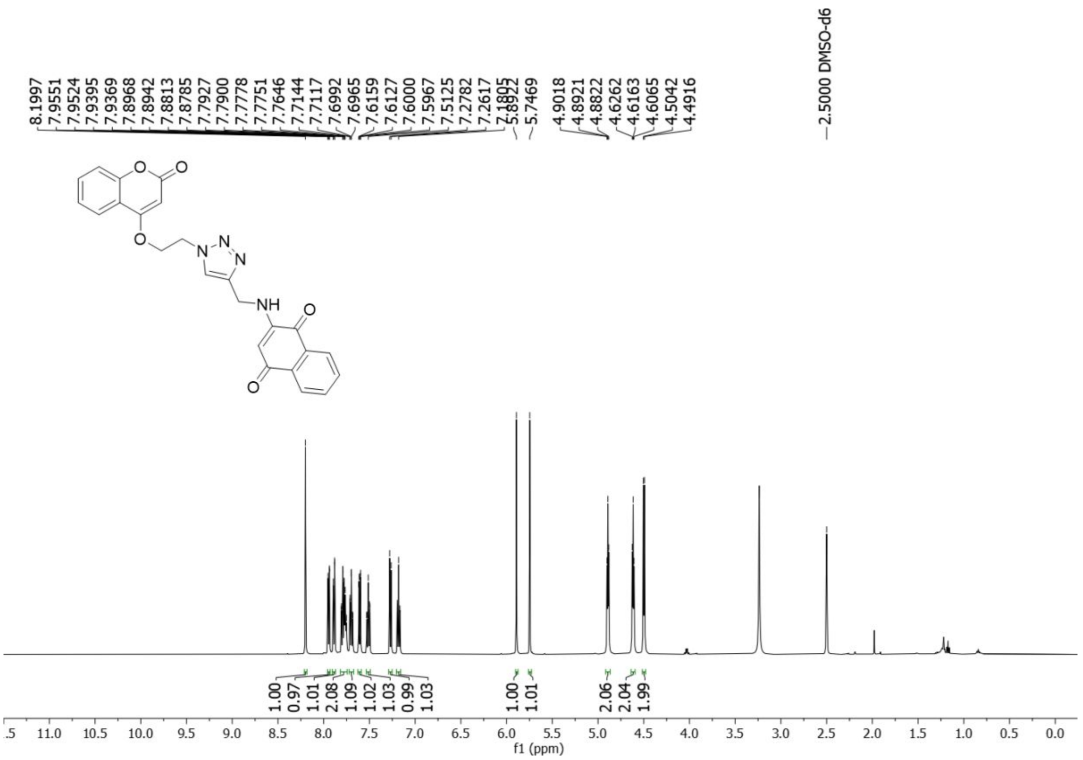

Figure S1. ^{1}H NMR spectrum (500.00 MHz, DMSO-d$_{6}$) of 2-(((1-(2-((2-oxo-2*H*-chromen-4-yl)oxy)ethyl)-1*H*-1,2,3-triazol-4-yl)methyl)amino) naphthalene-1,4-dione (**7a**)

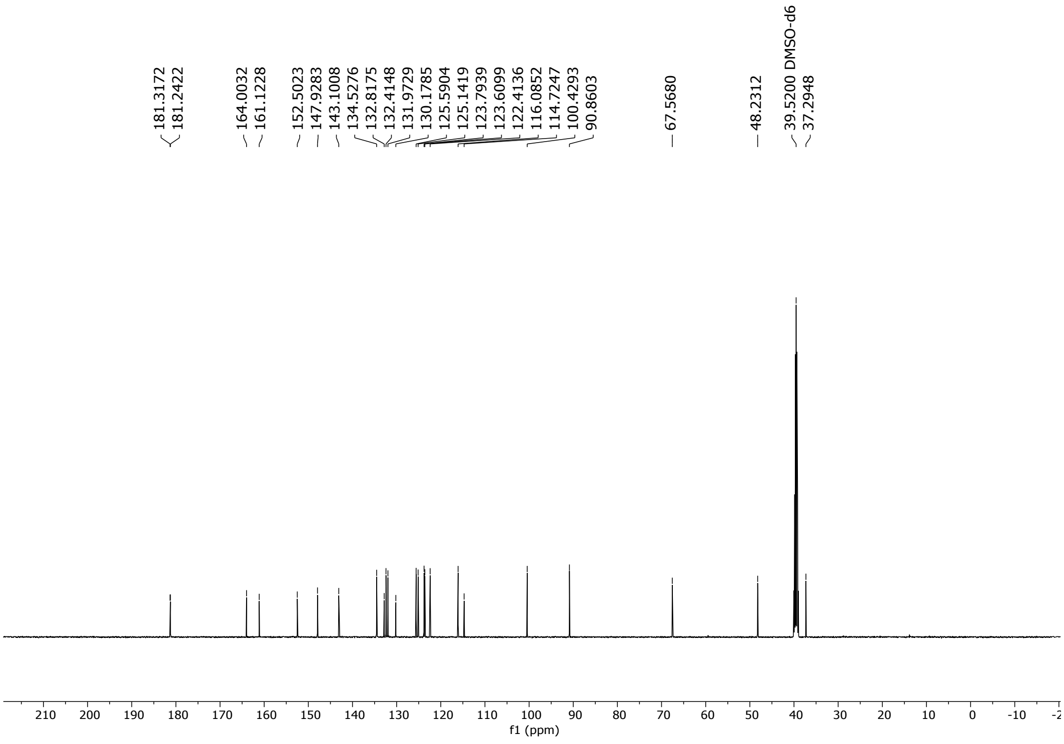

Figure S2. ^{13}C NMR spectrum (125.00 MHz, DMSO-d$_{6}$) of 2-(((1-(2-((2-oxo-2*H*-chromen-4-yl)oxy)ethyl)-1*H*-1,2,3-triazol-4-yl)methyl)amino) naphthalene-1,4-dione (**7a**)

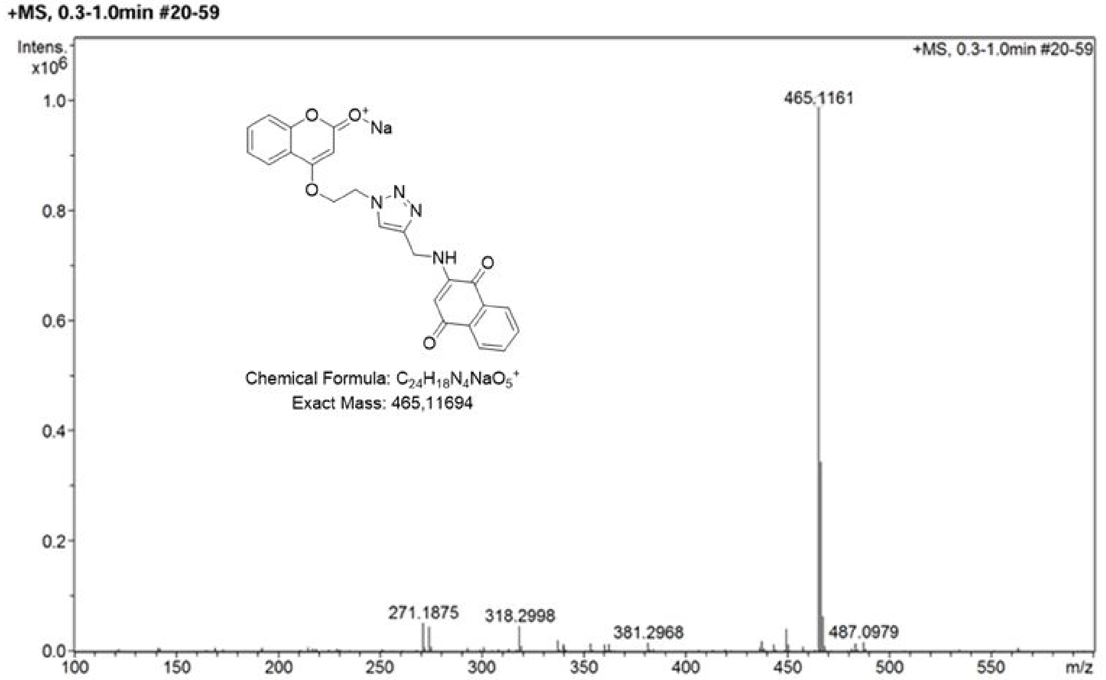

Figure S3. HRMS spectrum of 2-(((1-(2-((2-oxo-2*H*-chromen-4-yl)oxy)ethyl)-1H-1,2,3-triazol-4-yl)methyl)amino) naphthalene-1,4-dione (**7a**)

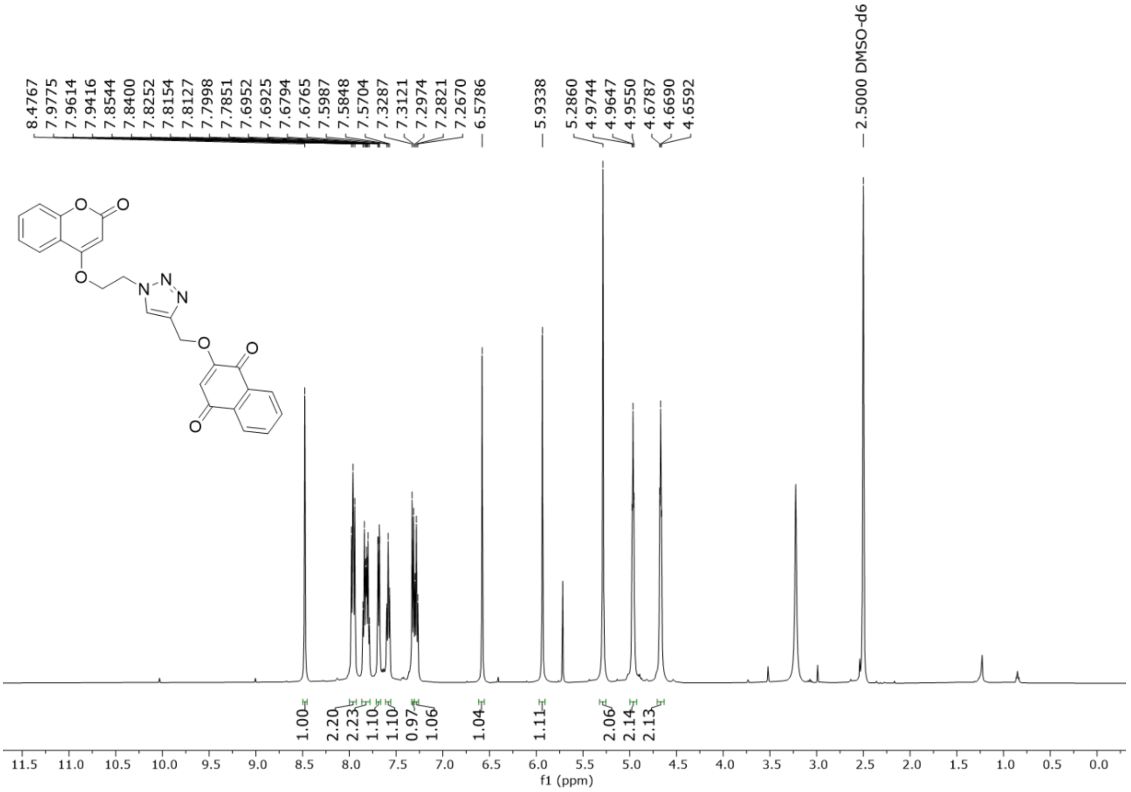

Figure S4. ^{1}H NMR spectrum (500.00 MHz, DMSO-d_6) of 2-((1-(2-((2-oxo-2*H*-chromen-4-yl)oxy)ethyl)-1*H*-1,2,3-triazol-4-yl)methoxy) naphthalene-1,4-dione (**7b**)

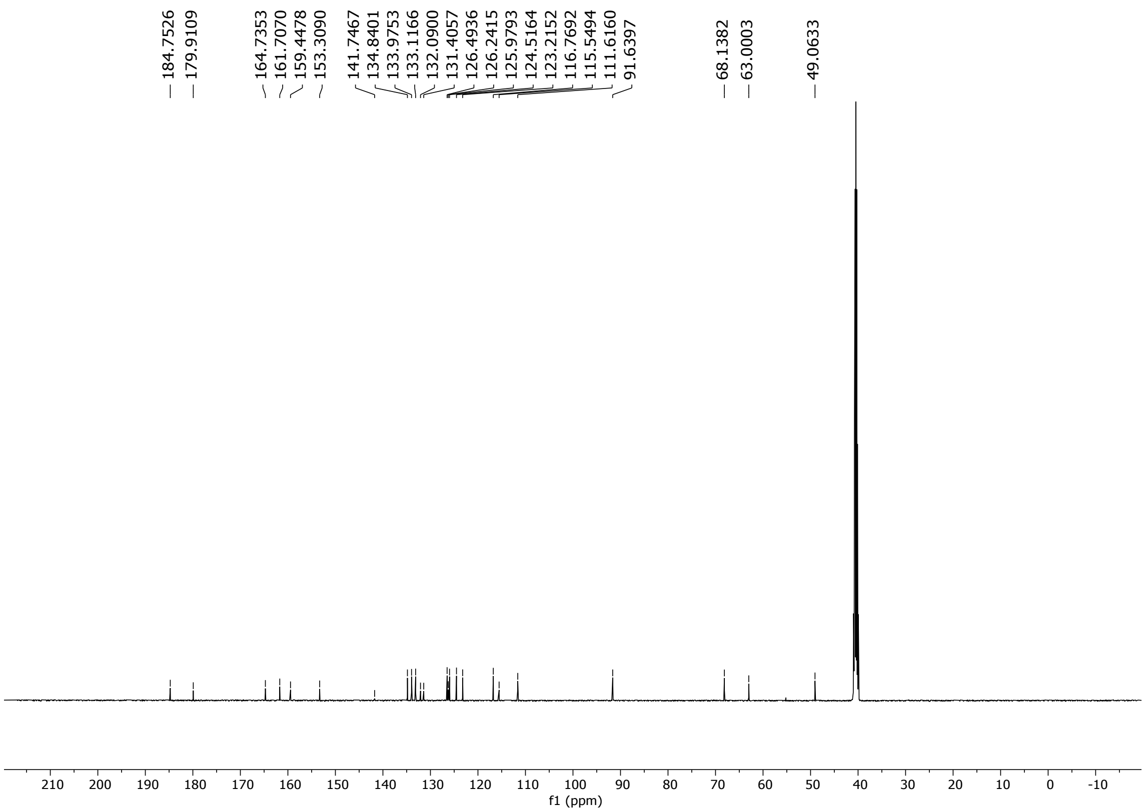

Figure S5. ^{13}C NMR spectrum (125.00 MHz, DMSO-d$_6$) of 2-((1-(2-((2-oxo-2*H*-chromen-4-yl)oxy)ethyl)-1*H*-1,2,3-triazol-4-yl)methoxy) naphthalene-1,4-dione (**7b**)

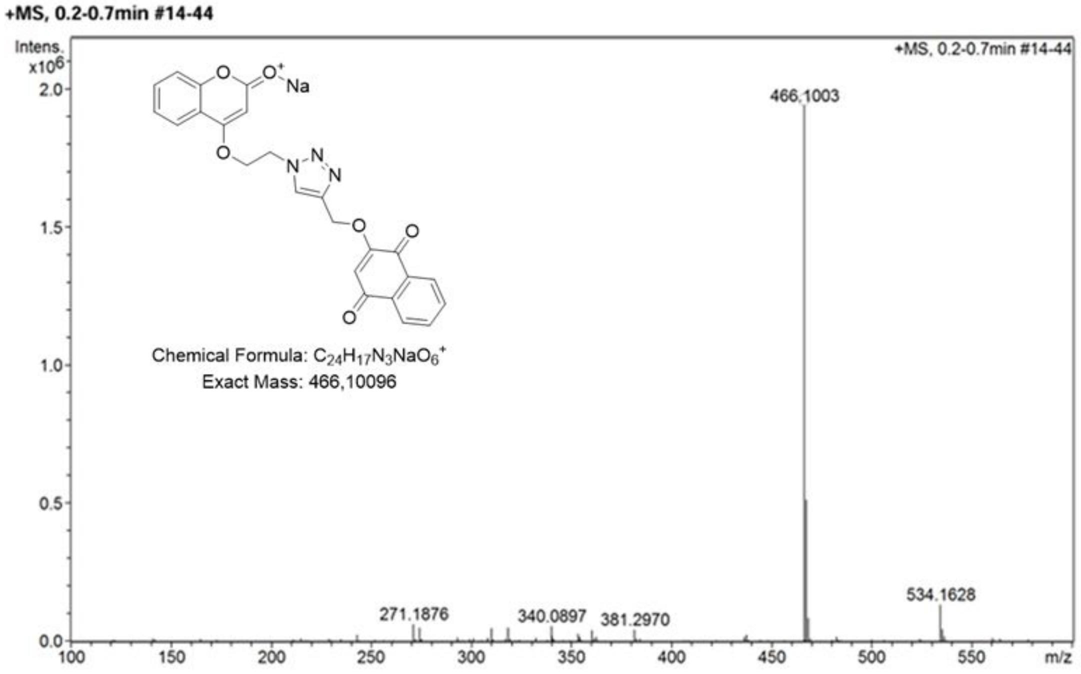

Figure S6. HRMS spectrum of 2-((1-(2-((2-oxo-2*H*-chromen-4-yl)oxy)ethyl)-1*H*-1,2,3-triazol-4-yl)methoxy) naphthalene-1,4-dione (**7b**)

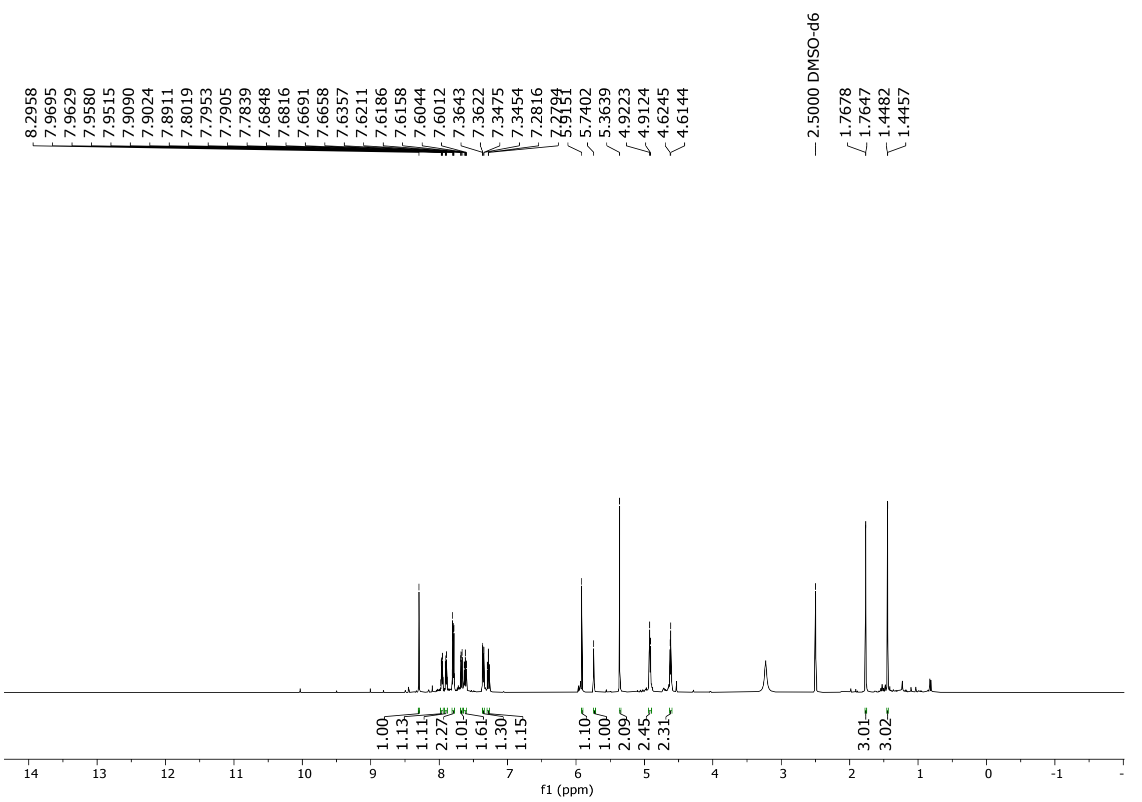

Figure S7. ^{1}H NMR spectrum (500.00 MHz, DMSO-d_6) of 2-(2-methylprop-1-en-1-yl)-3-((1-(2-((2-oxo-2*H*-chromen-4-yl)oxy)ethyl)-1*H*-1,2,3-triazol-4-yl)methoxy)naphthalene-1,4-dione (**7c**)

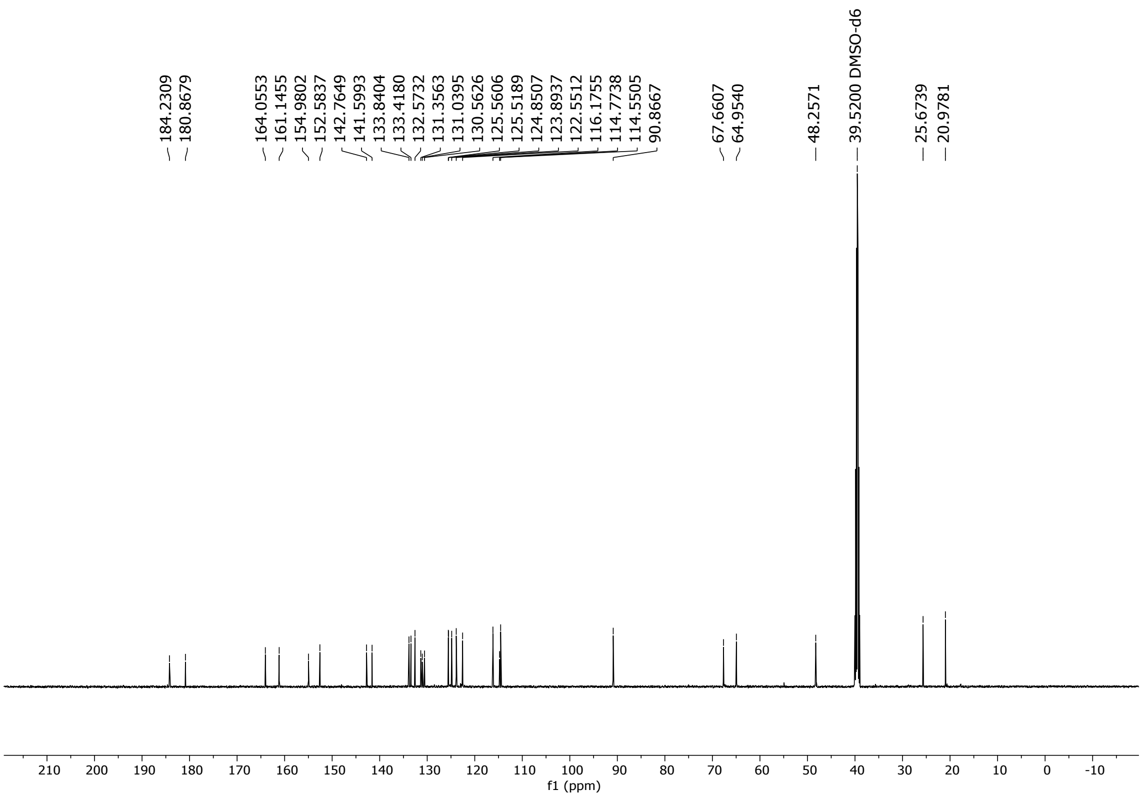

Figure S8. ^{13}C NMR spectrum (125.00 MHz, DMSO-d_6) of 2-(2-methylprop-1-en-1-yl)-3-((1-(2-((2-oxo-2*H*-chromen-4-yl)oxy)ethyl)-1*H*-1,2,3-triazol-4-yl)methoxy)naphthalene-1,4-dione (**7c**)

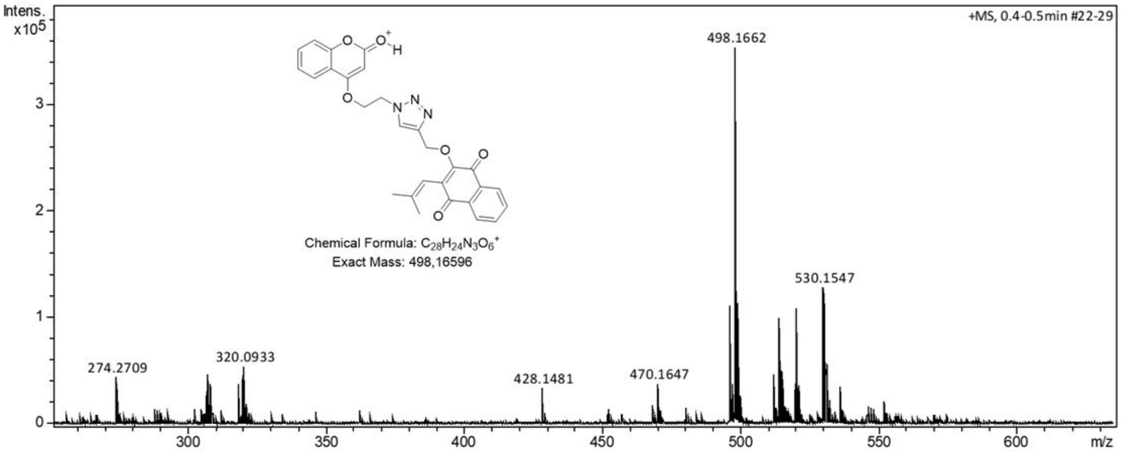

Figure S9. HRMS spectrum of 2-(2-methylprop-1-en-1-yl)-3-((1-(2-((2-oxo-2*H*-chromen-4-yl)oxy)ethyl)-1*H*-1,2,3-triazol-4-yl)methoxy)naphthalene-1,4-dione (**7c**)

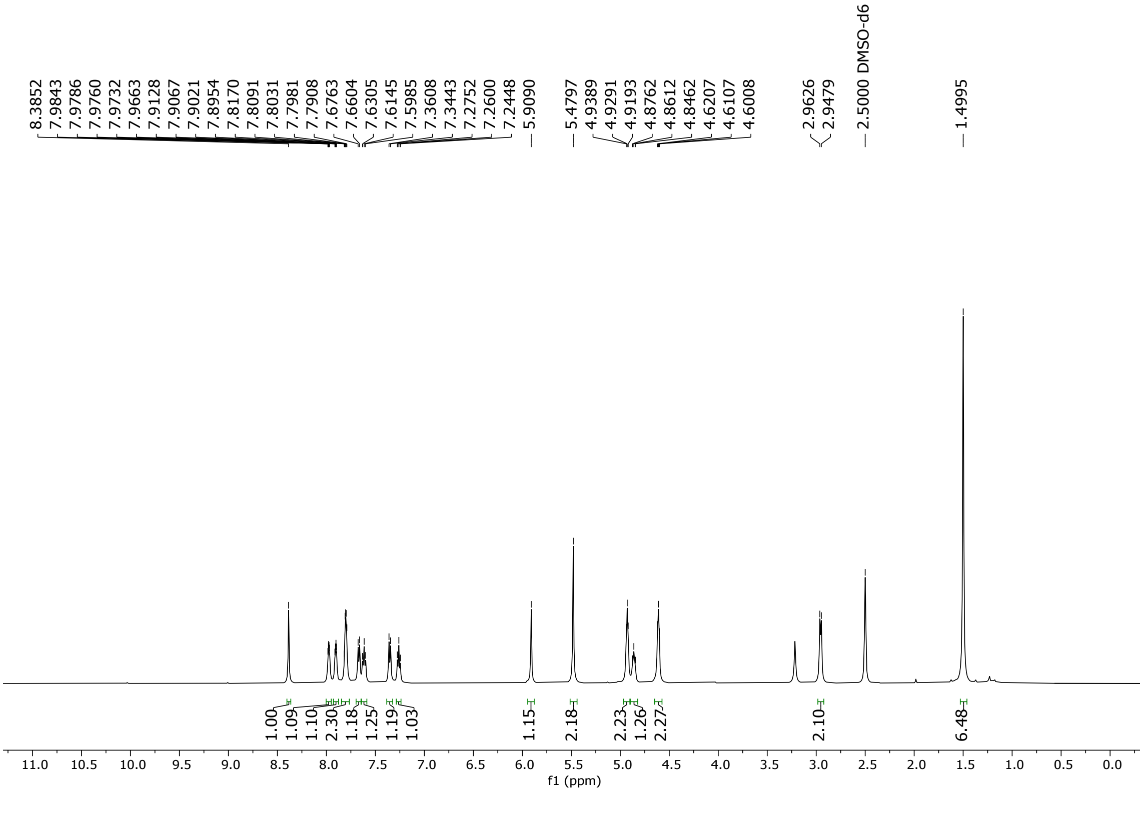

Figure S10. ^1H NMR spectrum (500.00 MHz, DMSO-d_6) of 2-(3-methylbut-2-en-1-yl)-3-((1-(2-((2-oxo-2*H*-chromen-4-yl)oxy)ethyl)-1*H*-1,2,3-triazol-4-yl)methoxy)naphthalene-1,4-dione (**7d**)

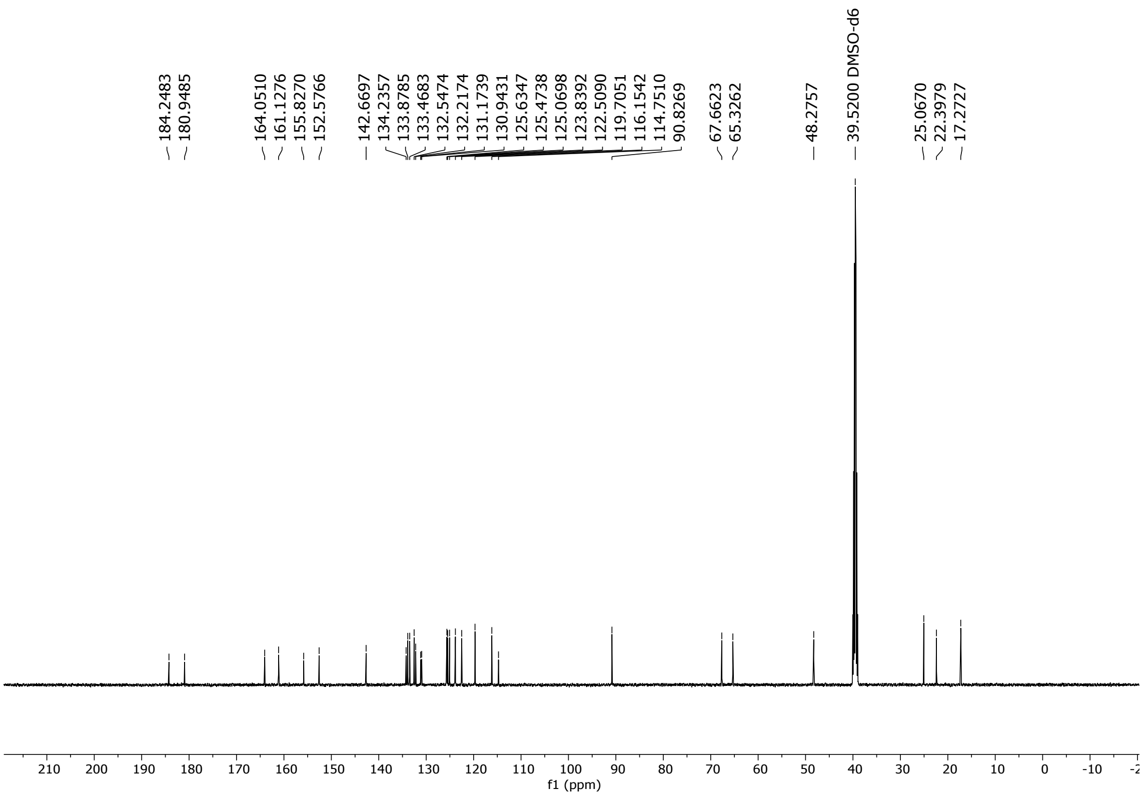

Figure S11. ^{13}C NMR spectrum (125.00 MHz, DMSO-d_6) of 2-(3-methylbut-2-en-1-yl)-3-((1-(2-((2-oxo-2*H*-chromen-4-yl)oxy)ethyl)-1*H*-1,2,3-triazol-4-yl)methoxy)naphthalene-1,4-dione (**7d**)

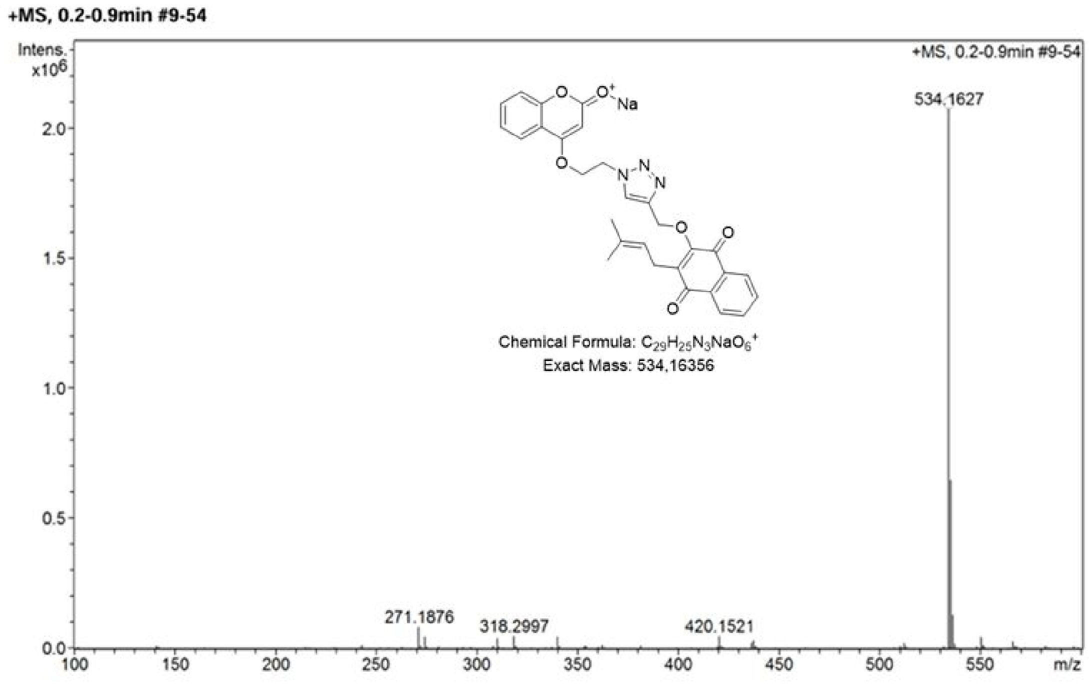

Figure S12. HRMS spectrum of 2-(3-methylbut-2-en-1-yl)-3-((1-(2-((2-oxo-2*H*-chromen-4-yl)oxy)ethyl)-1*H*-1,2,3-triazol-4-yl)methoxy)naphthalene-1,4-dione (**7d**)

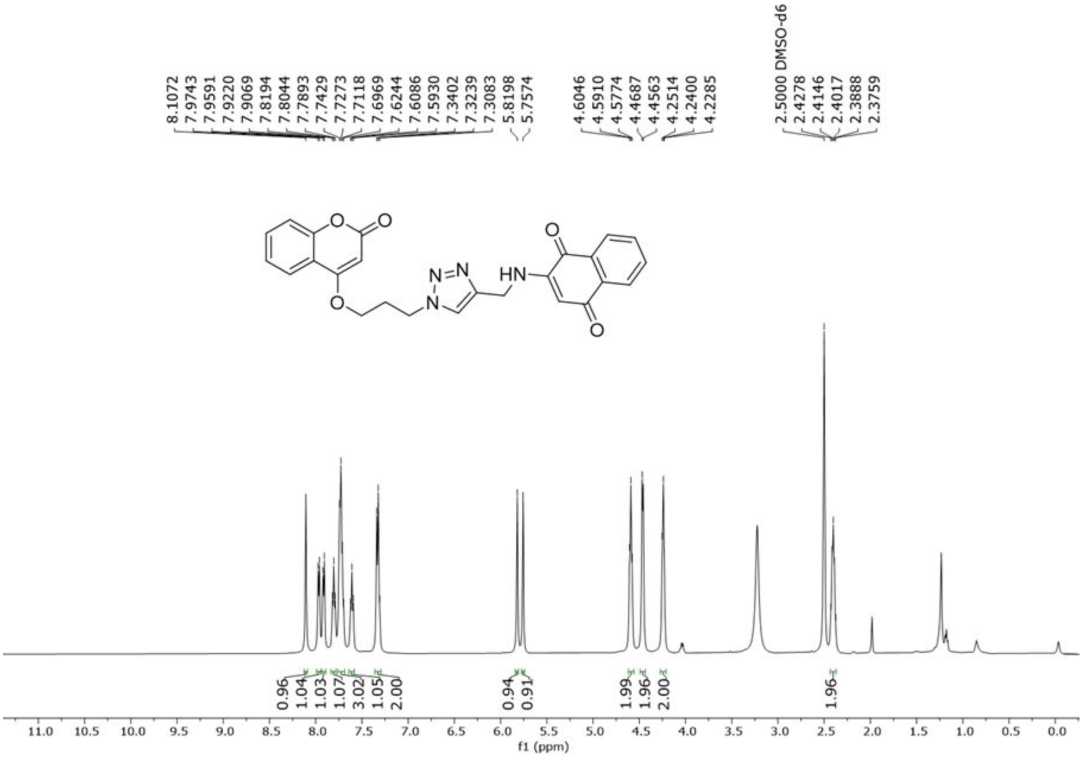

Figure S13. ^{1}H NMR spectrum (500.00 MHz, DMSO-d$_{6}$) of 2-(((1-(3-((2-oxo-2*H*-chromen-4-yl)oxy)propyl)-1*H*-1,2,3-triazol-4-yl)methyl)amino) naphthalene-1,4-dione (**7e**)

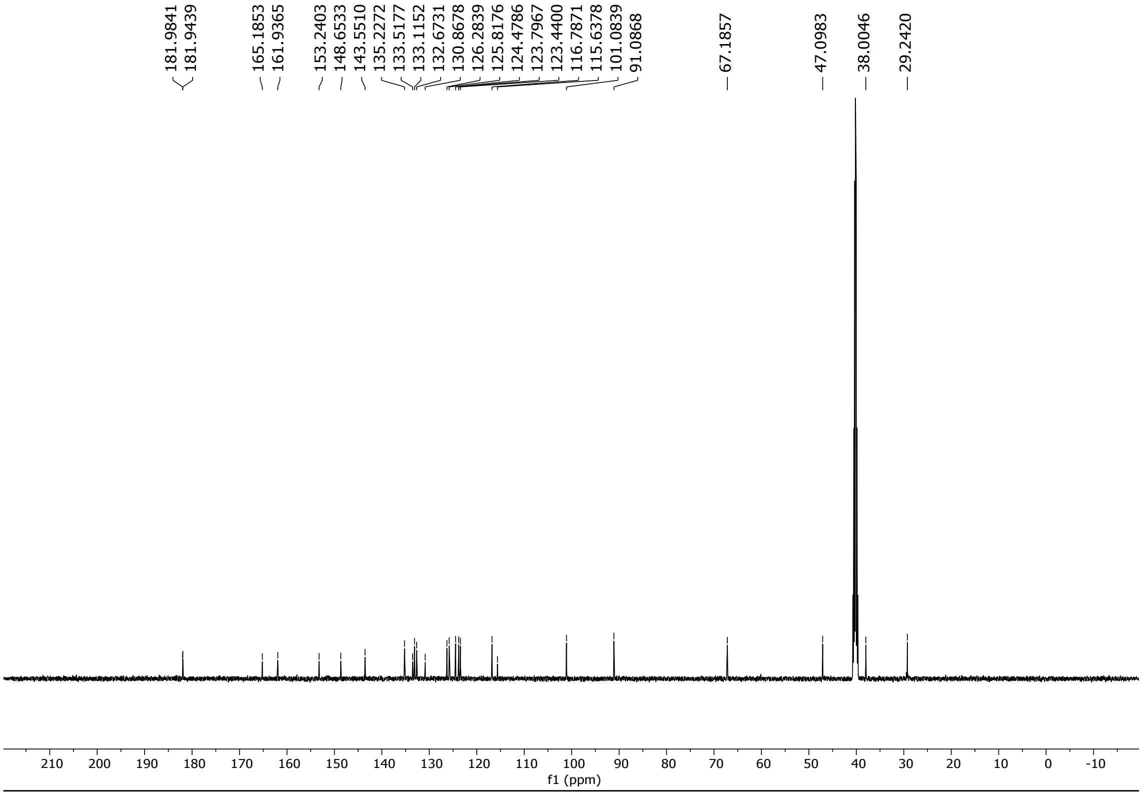

Figure S14. ^{13}C NMR spectrum (125.00 MHz, DMSO-d$_{6}$) of 2-(((1-(3-((2-oxo-2*H*-chromen-4-yl)oxy)propyl)-1*H*-1,2,3-triazol-4-yl)methyl)amino) naphthalene-1,4-dione (**7e**)

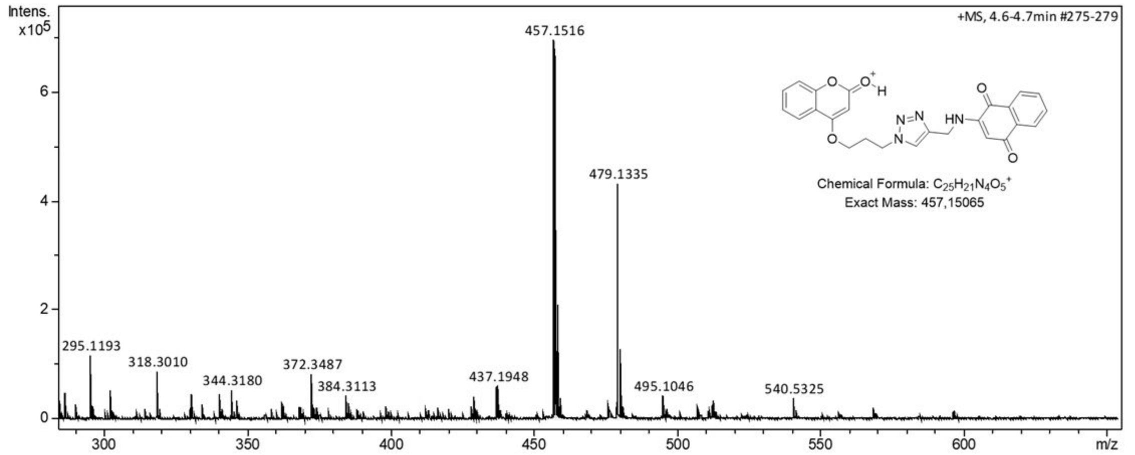

Figure S15. HRMS spectrum of 2-(((1-(3-((2-oxo-2*H*-chromen-4-yl)oxy)propyl)-1*H*-1,2,3-triazol-4-yl)methyl)amino) naphthalene-1,4-dione (**7e**)

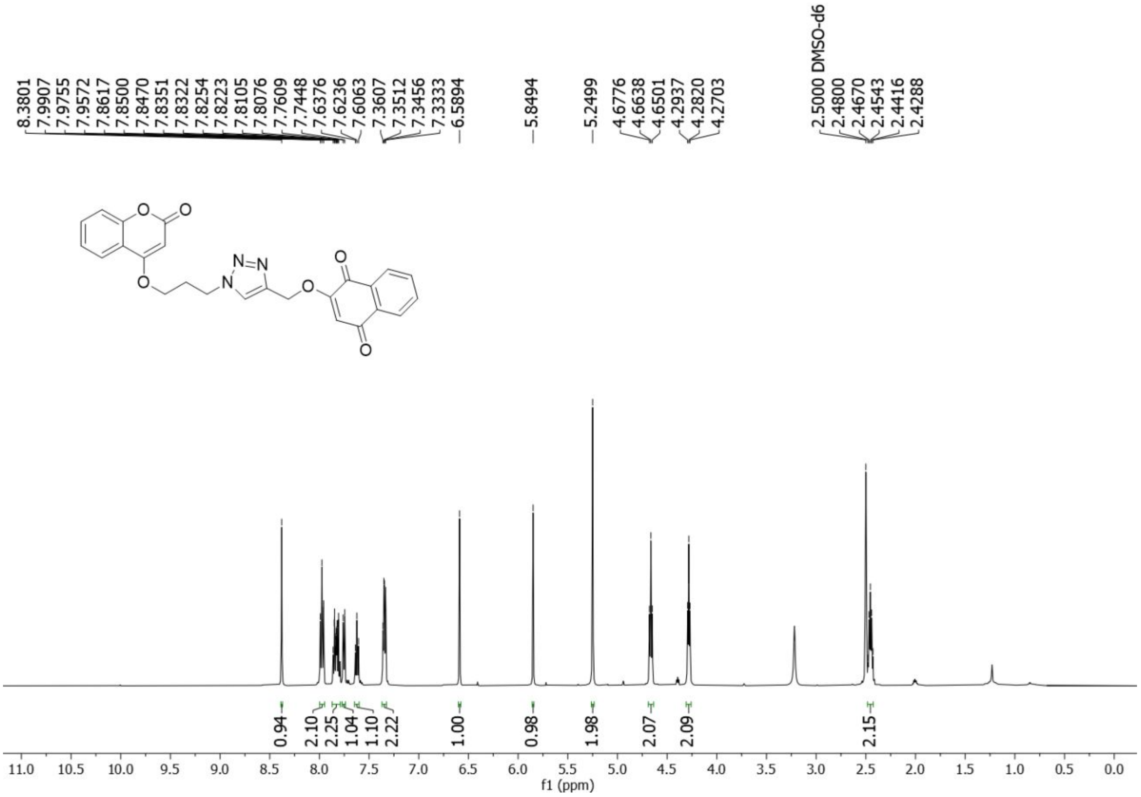

Figure S16. ^{1}H NMR spectrum (500.00 MHz, DMSO-d$_6$) of 2-((1-(3-((2-oxo-2*H*-chromen-4-yl)oxy)propyl)-1*H*-1,2,3-triazol-4-yl)methoxy) naphthalene-1,4-dione (**7f**)

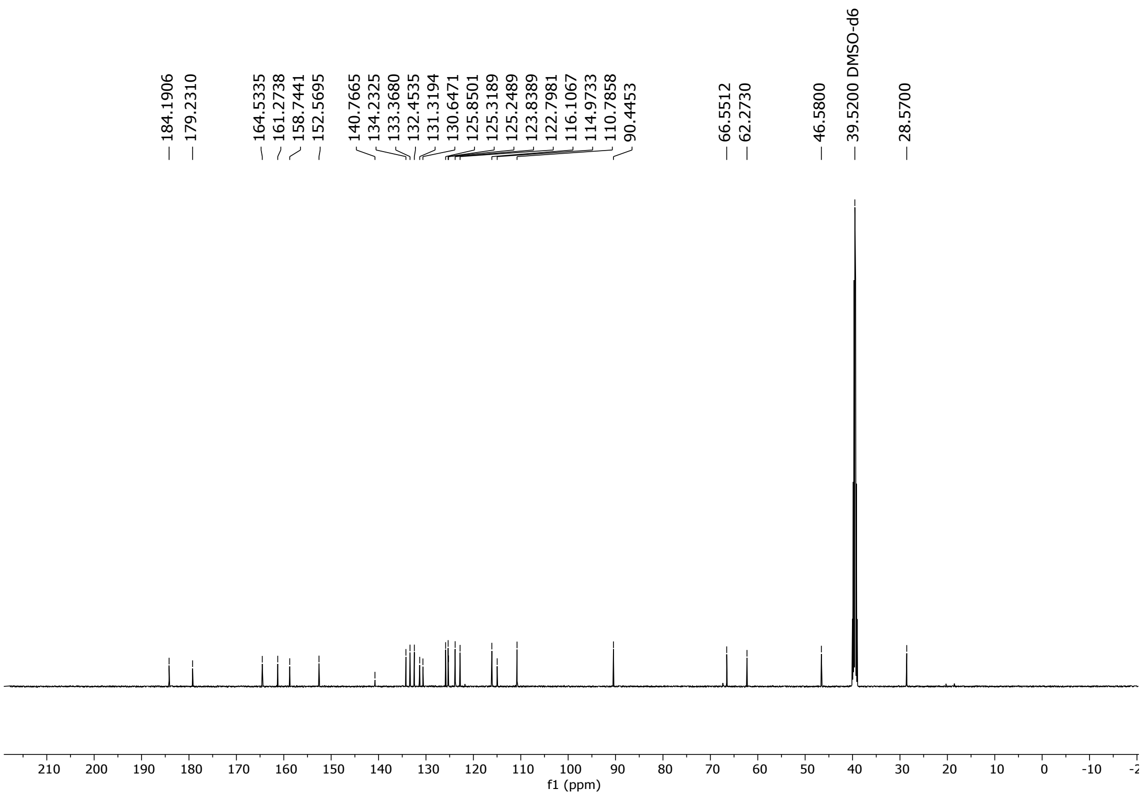

Figure S17. ^{13}C NMR spectrum (125.00 MHz, DMSO-d$_6$) of 2-((1-(3-((2-oxo-2*H*-chromen-4-yl)oxy)propyl)-1*H*-1,2,3-triazol-4-yl)methoxy) naphthalene-1,4-dione (**7f**)

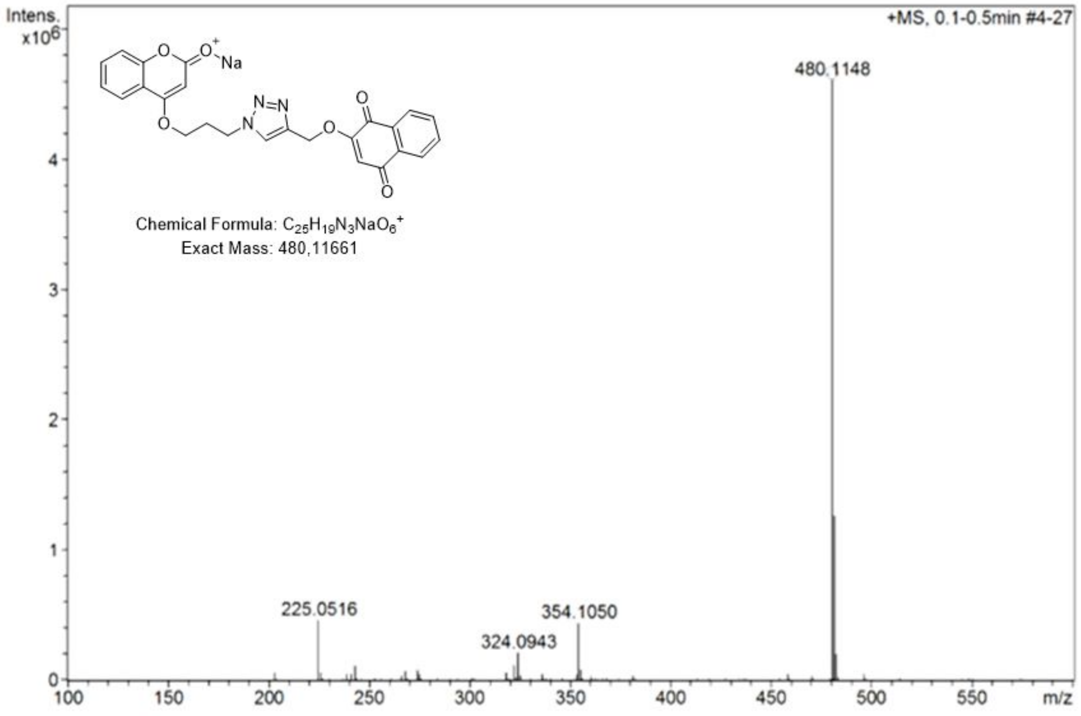

Figure S18. HRMS spectrum of 2-((1-(3-((2-oxo-2*H*-chromen-4-yl)oxy)propyl)-1*H*-1,2,3-triazol-4-yl)methoxy) naphthalene-1,4-dione (**7f**)

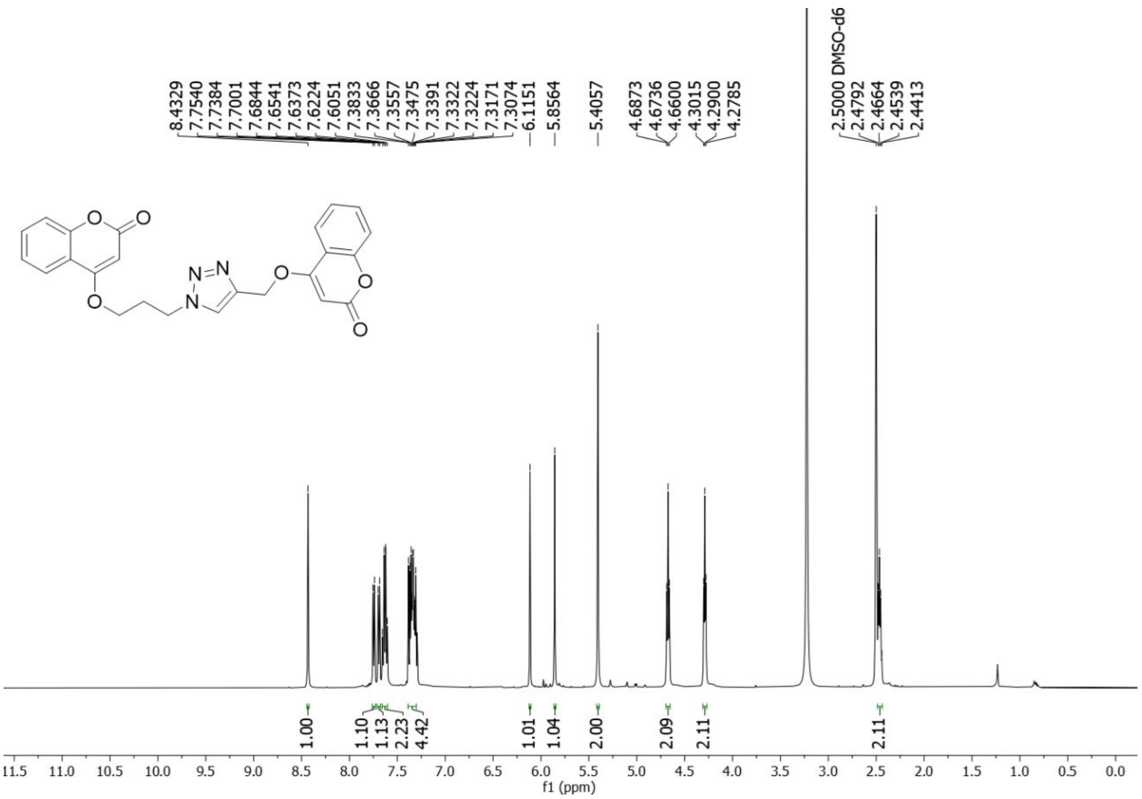

Figure S19. ^{1}H NMR spectrum (500.00 MHz, DMSO-d$_6$) of 4-(3-(4-(((2-oxo-2*H*-chromen-4-yl)oxy)methyl)-1*H*-1,2,3-triazol-1-yl)propoxy)-2*H*-chromen-2-one (**7g**)

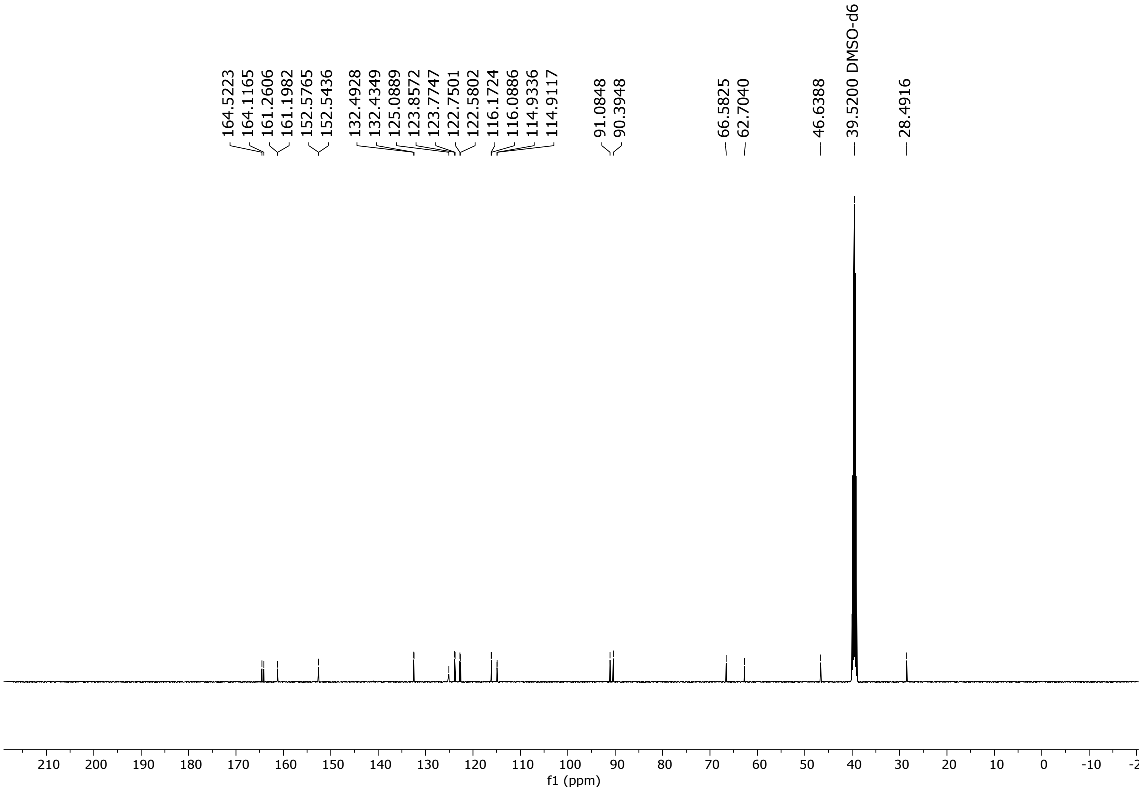

Figure S20. ^{13}C NMR spectrum (125.00 MHz, DMSO-d$_6$) of 4-(3-(4-(((2-oxo-2*H*-chromen-4-yl)oxy)methyl)-1*H*-1,2,3-triazol-1-yl)propoxy)-2*H*-chromen-2-one (**7g**)

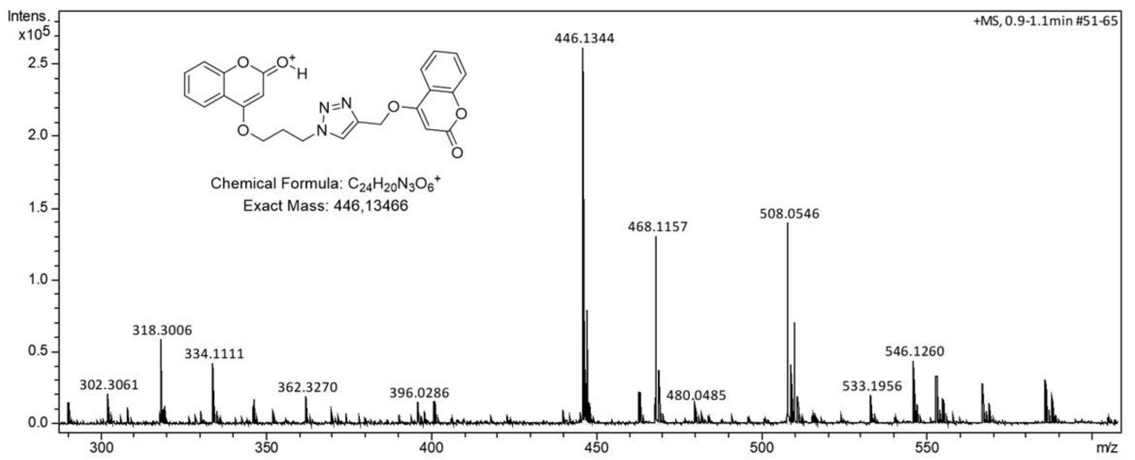

Figure S21. HRMS spectrum of 4-(3-(4-(((2-oxo-2*H*-chromen-4-yl)oxy)methyl)-1*H*-1,2,3-triazol-1-yl)propoxy)-2*H*-chromen-2-one (**7g**)

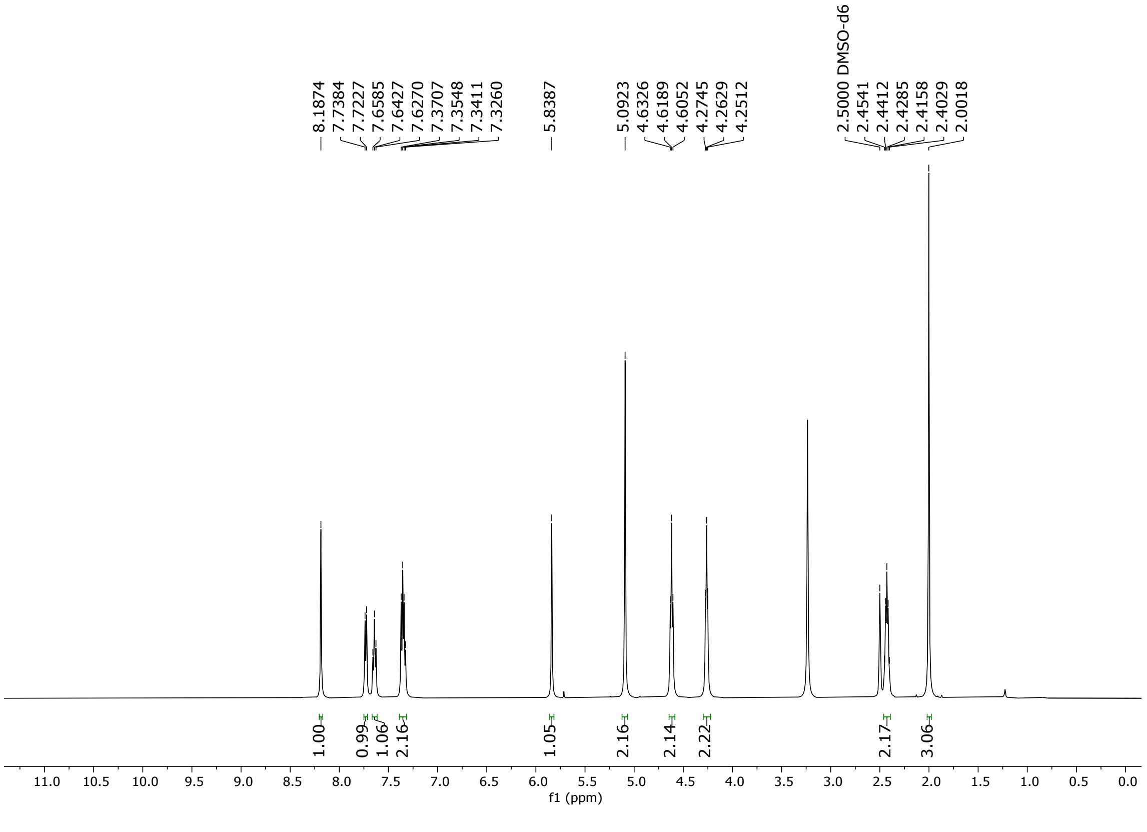

Figure S22. ^{1}H NMR spectrum (500.00 MHz, DMSO-d$_6$) of (1-(3-((2-oxo-2*H*-chromen-4-yl)oxy)propyl)-1*H*-1,2,3-triazol-4-yl)methylacetate (**7h**)

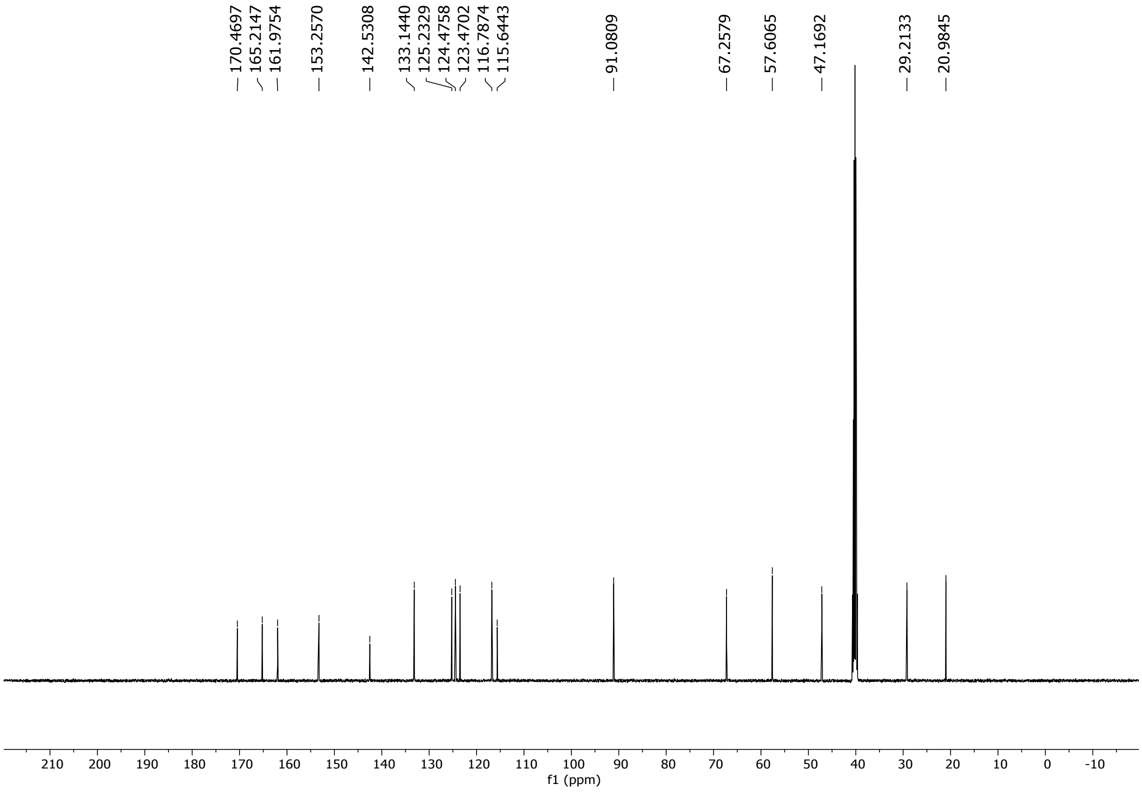

Figure S23. ^{13}C NMR spectrum (125.00 MHz, DMSO-d$_6$) of (1-(3-((2-oxo-2*H*-chromen-4-yl)oxy)propyl)-1*H*-1,2,3-triazol-4-yl)methylacetate (**7h**)

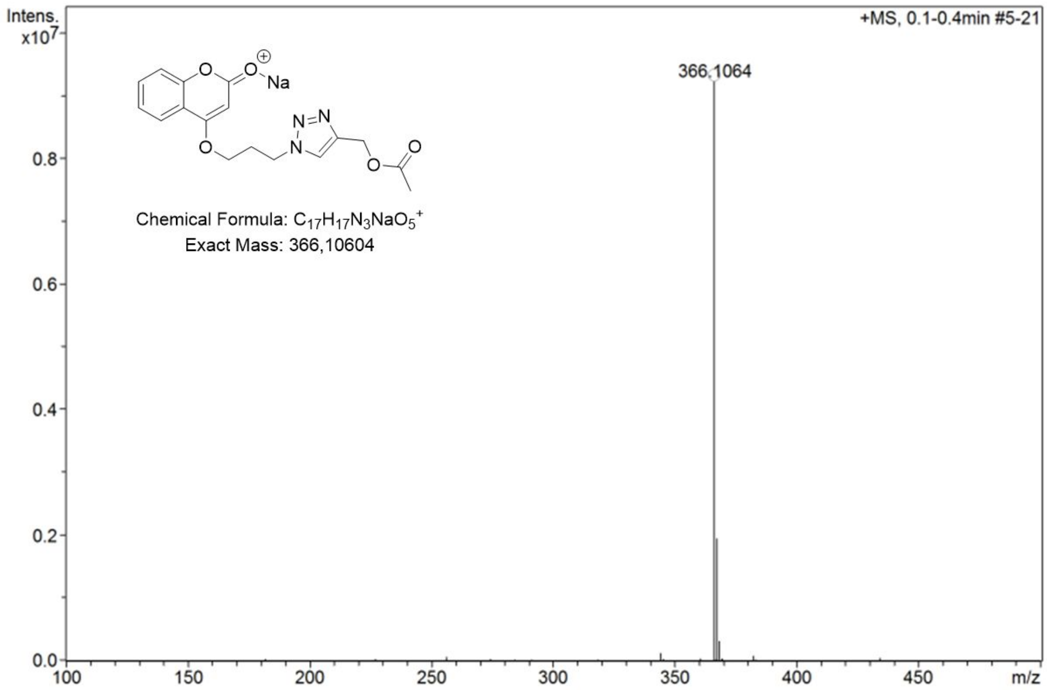

Figure S24. HRMS spectrum of (1-(3-((2-oxo-2*H*-chromen-4-yl)oxy)propyl)-1*H*-1,2,3-triazol-4-yl)methylacetate (**7h**)

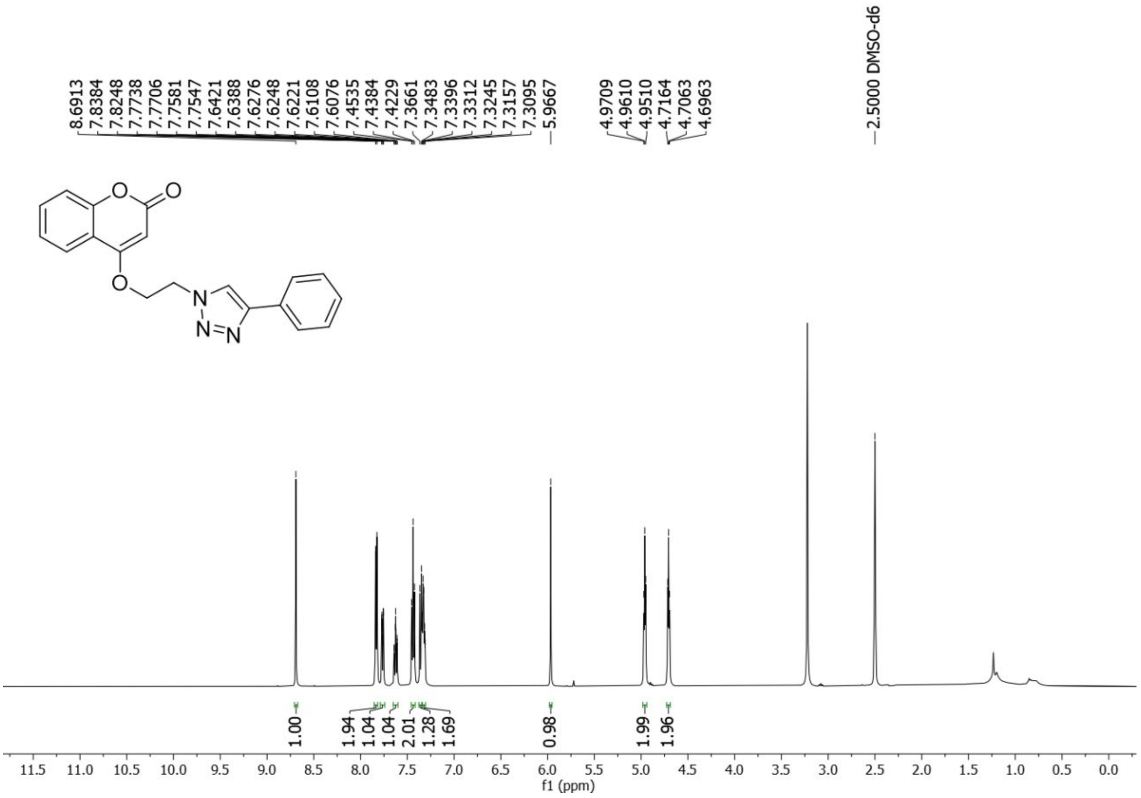

Figure S25. ^{1}H NMR spectrum (500.00 MHz, DMSO-d$_6$) of 4-(2-(4-phenyl-1*H*-1,2,3-triazol-1-yl)ethoxy)-2*H*-chromen-2-one (**7i**)

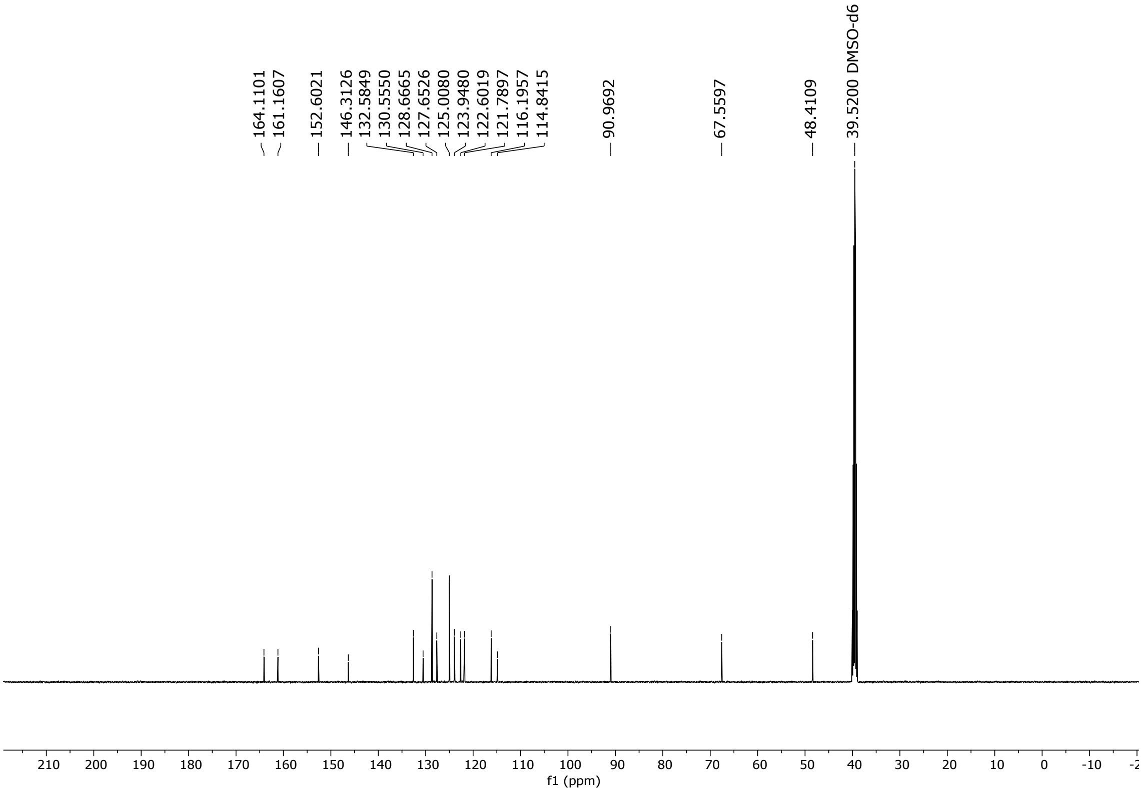

Figure S26. ^{13}C NMR spectrum (125.00 MHz, DMSO-d$_6$) of 4-(2-(4-phenyl-1*H*-1,2,3-triazol-1-yl)ethoxy)-2*H*-chromen-2-one (**7i**)

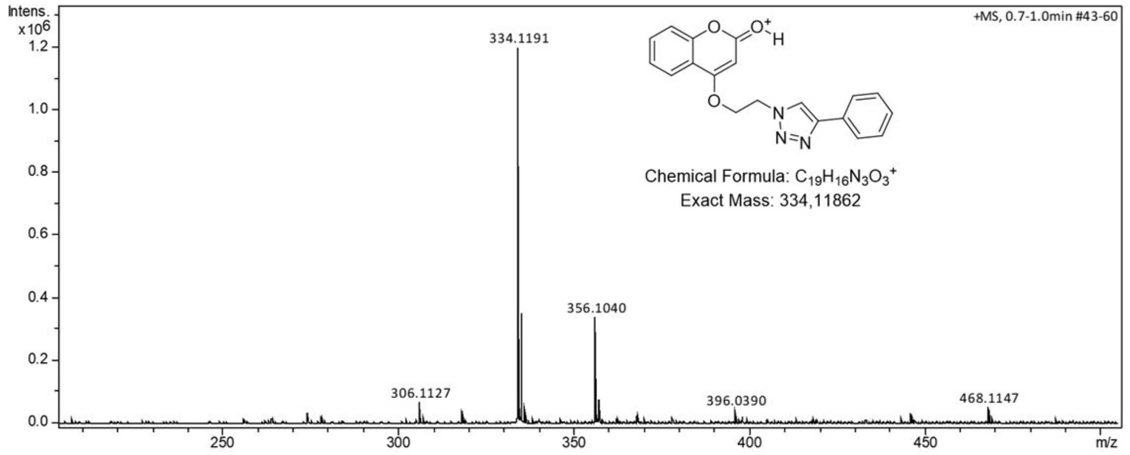

Figure S27. HRMS spectrum of 4-(2-(4-phenyl-1H-1,2,3-triazol-1-yl)ethoxy)-2H-chromen-2-one (7i)

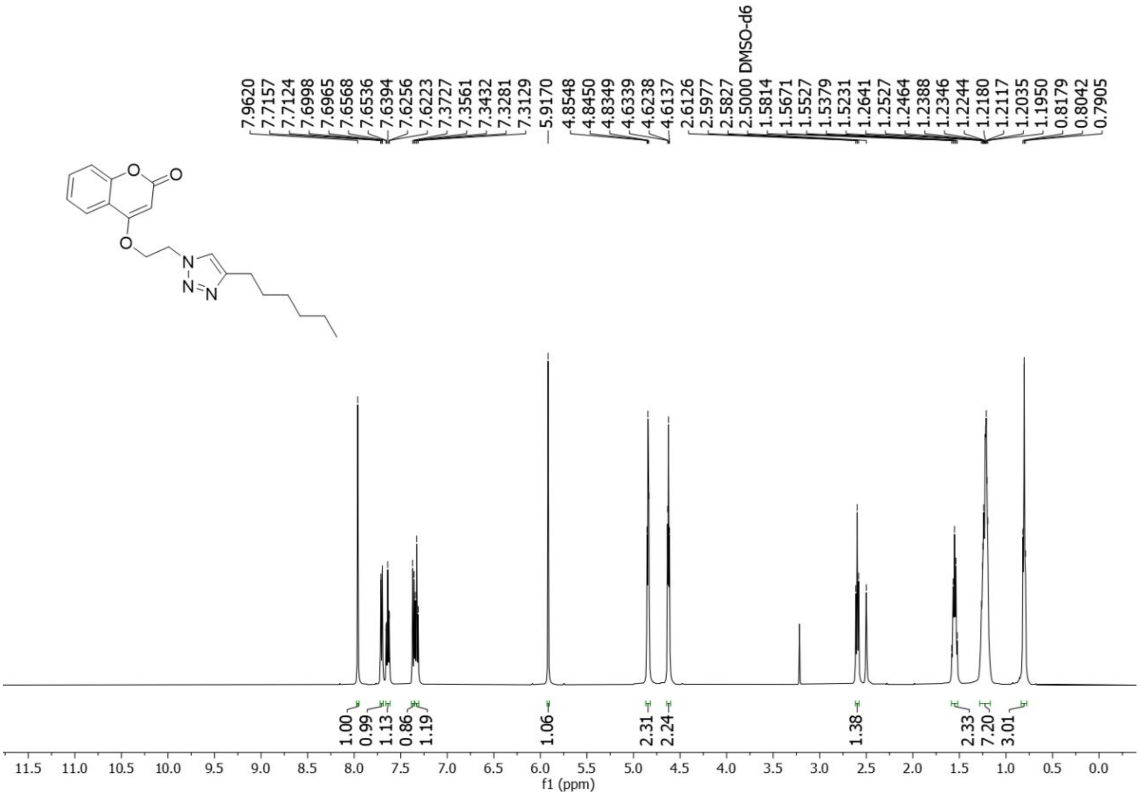

Figure S28. ^{1}H NMR spectrum (500.00 MHz, DMSO-d_6) of 4-(2-(4-hexyl-1*H*-1,2,3-triazol-1-yl)ethoxy)-2*H*-chromen-2-one (**7j**)

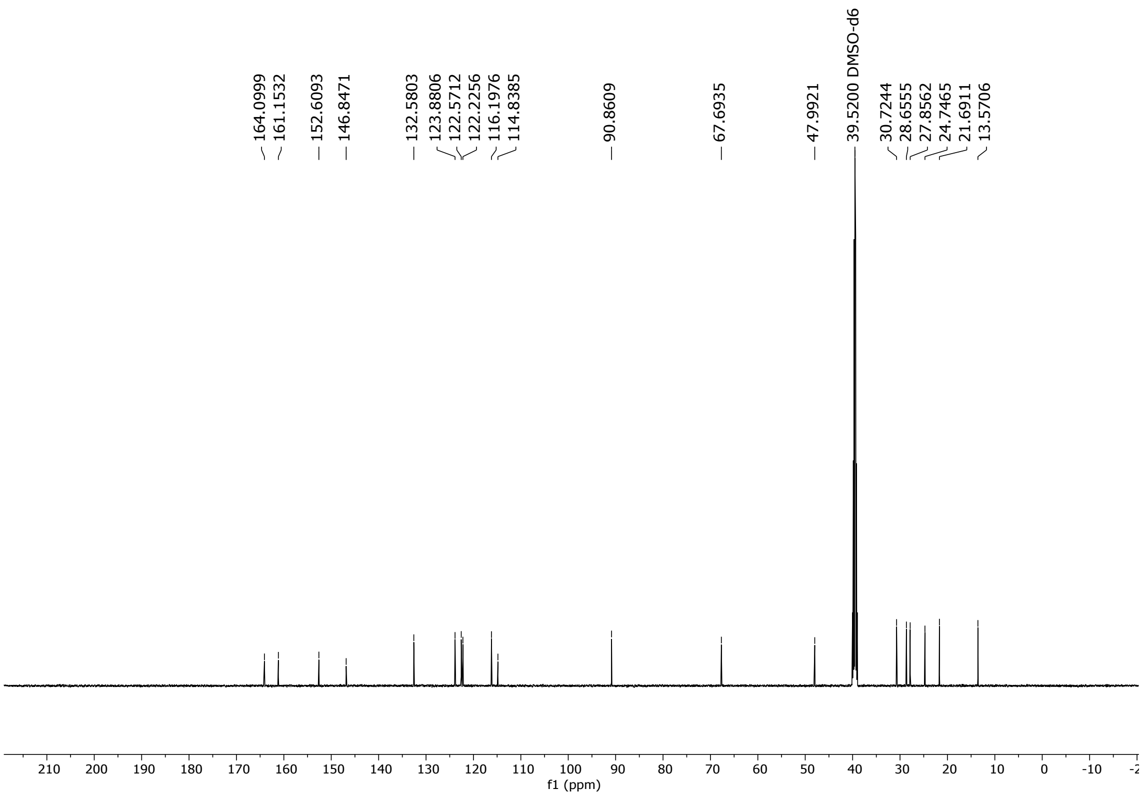

Figure S29. ^{13}C NMR spectrum (125.00 MHz, DMSO-d_6) of 4-(2-(4-hexyl-1*H*-1,2,3-triazol-1-yl)ethoxy)-2*H*-chromen-2-one (**7j**)

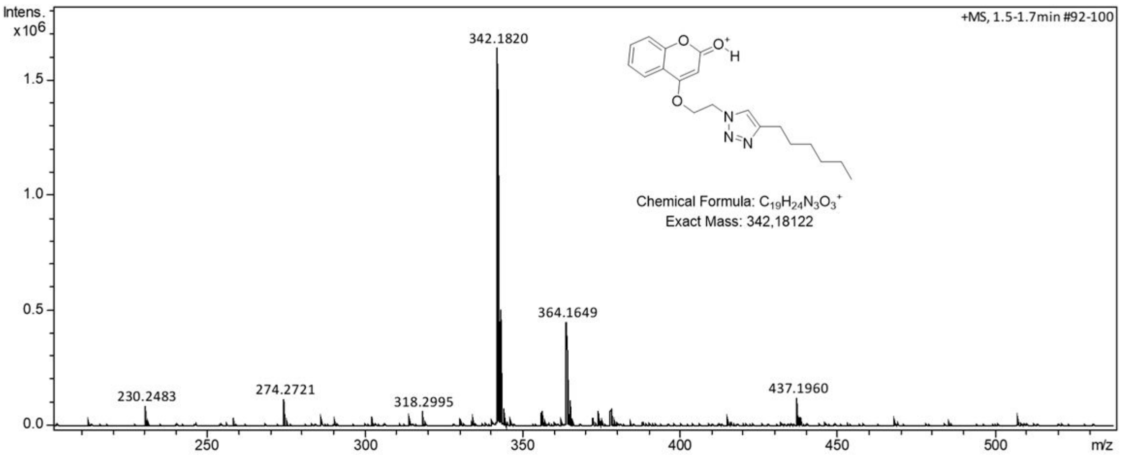

Figure S30. HRMS spectrum of 4-(2-(4-hexyl-1*H*-1,2,3-triazol-1-yl)ethoxy)-2*H*-chromen-2-one (**7j**)

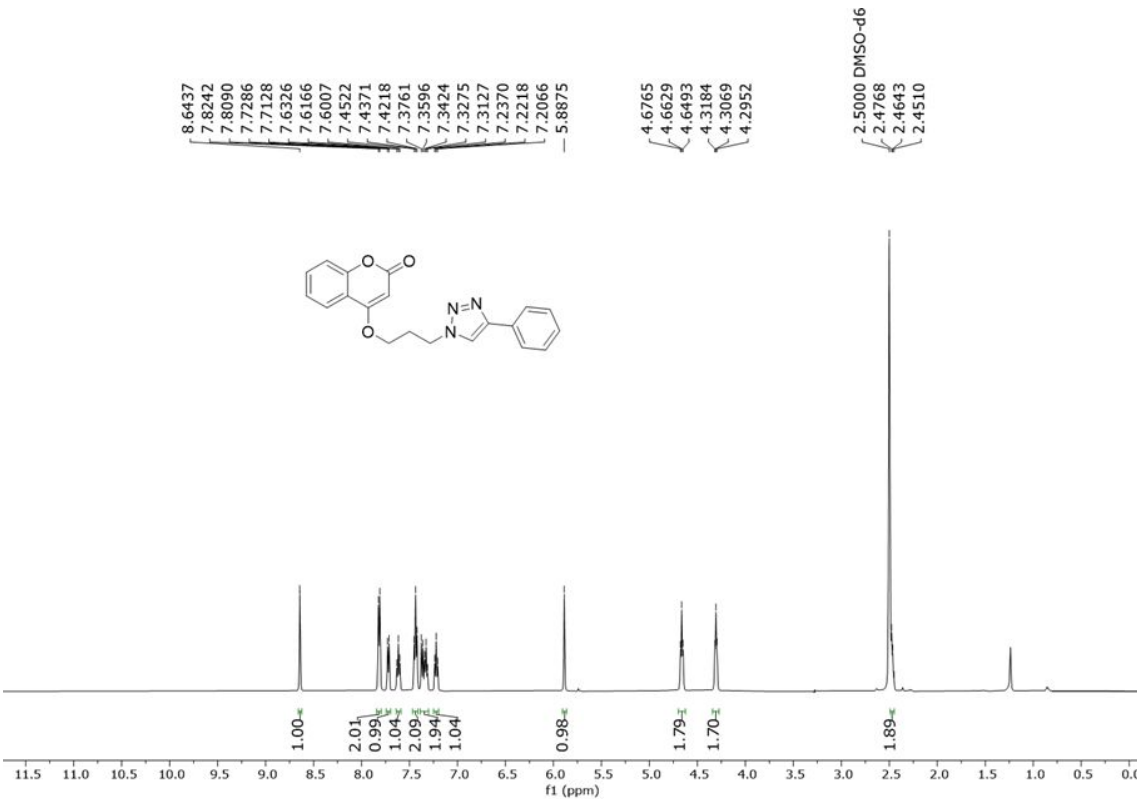

Figure S31. ^{1}H NMR spectrum (500.00 MHz, DMSO-d$_6$) of 4-(3-(4-phenyl-1*H*-1,2,3-triazol-1-yl)propoxy)-2*H*-chromen-2-one (**7k**)

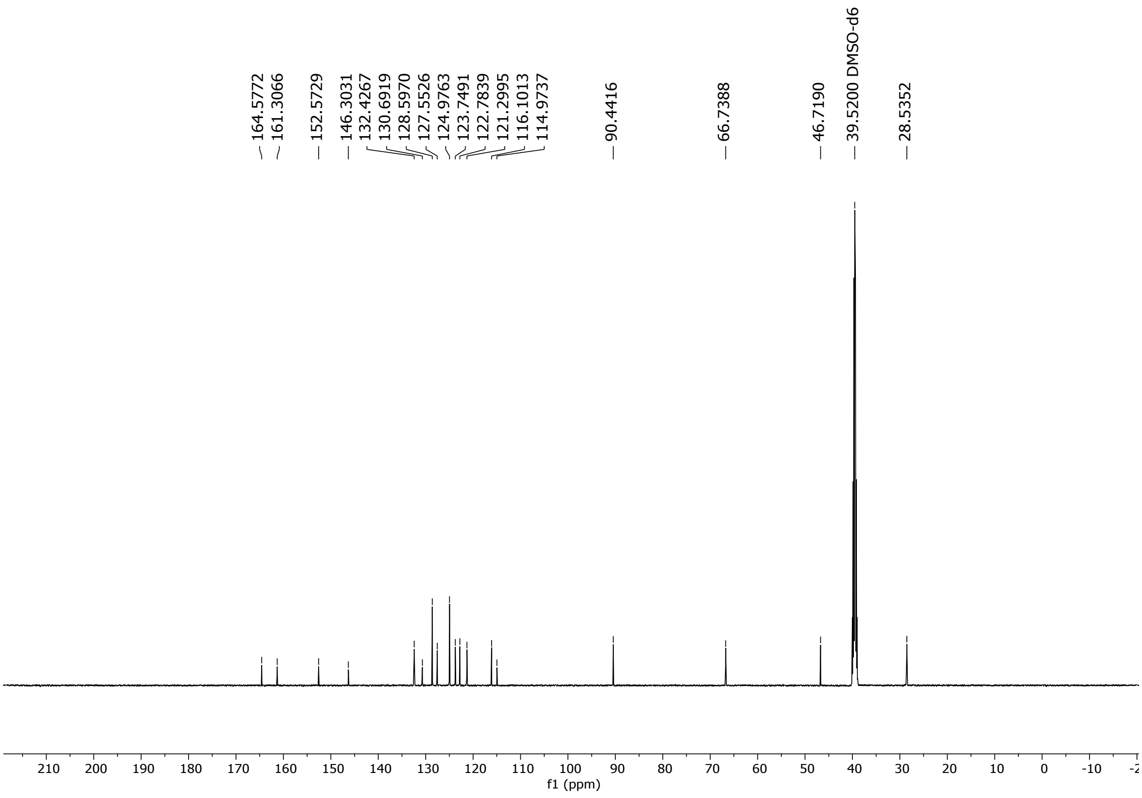

Figure S32. ^{13}C NMR spectrum (125.00 MHz, DMSO-d$_6$) of 4-(3-(4-phenyl-1*H*-1,2,3-triazol-1-yl)propoxy)-2*H*-chromen-2-one (**7k**)

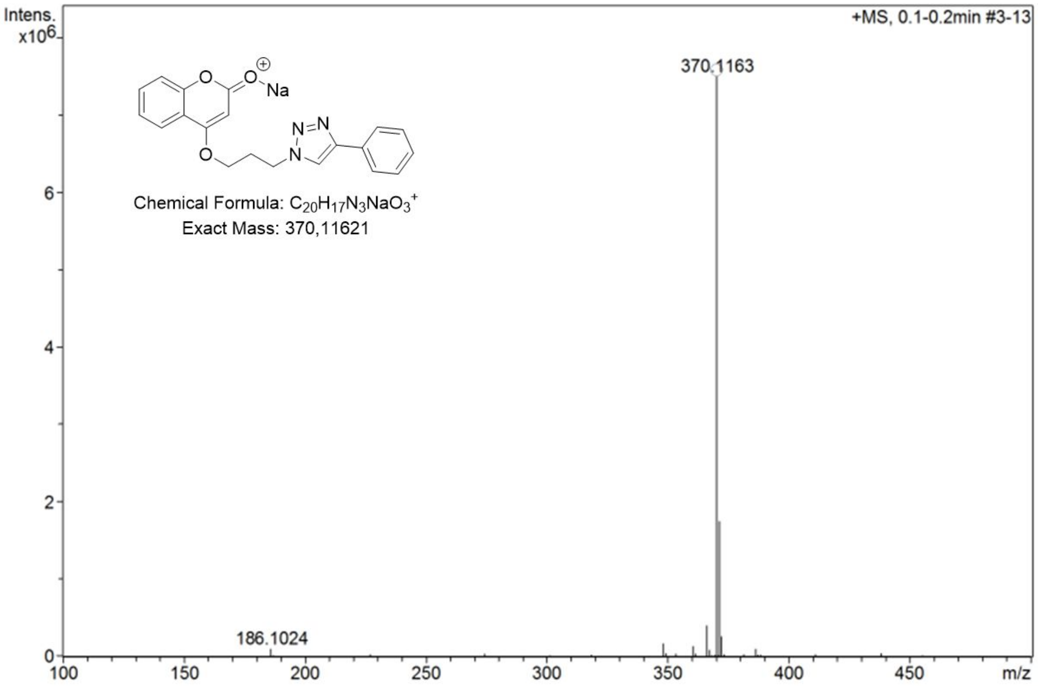

Figure S33. HRMS spectrum of 4-(3-(4-phenyl-1*H*-1,2,3-triazol-1-yl)propoxy)-2*H*-chromen-2-one (**7k**)

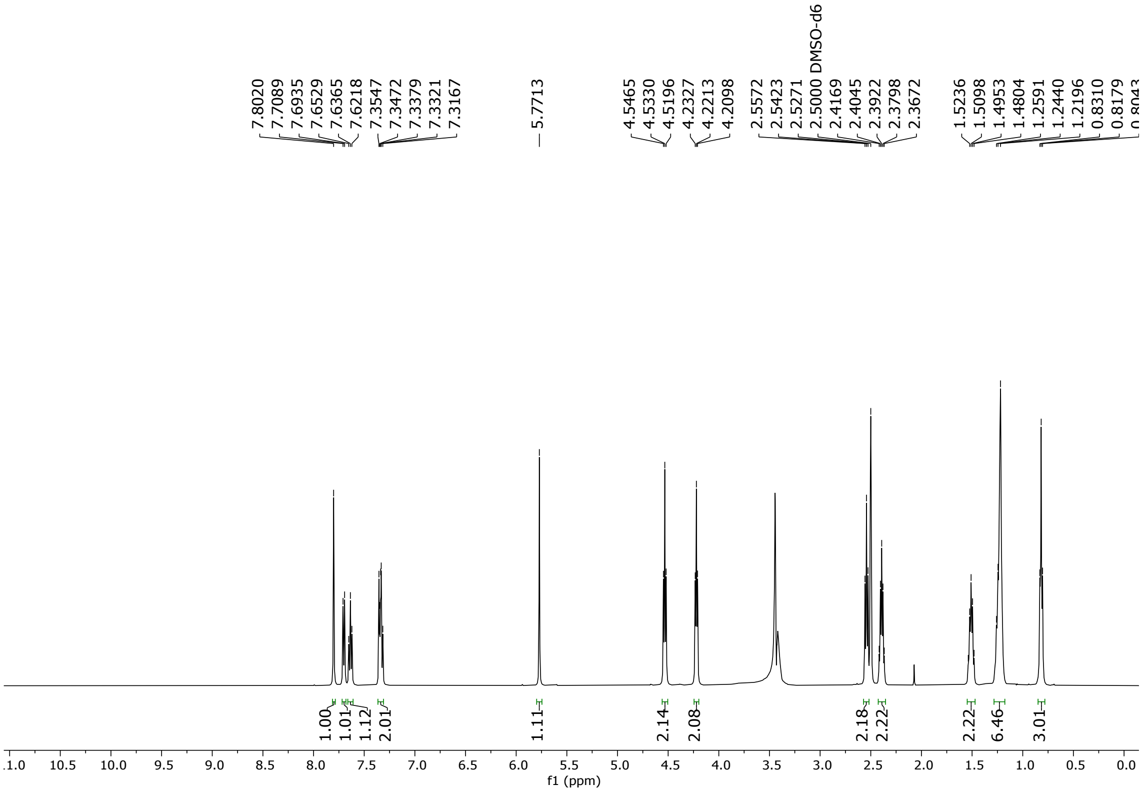

Figure S34. ^{1}H NMR spectrum (500.00 MHz, DMSO-d$_6$) of 4-(3-(4-hexyl-1*H*-1,2,3-triazol-1-yl)propoxy)-2*H*-chromen-2-one (**7l**)

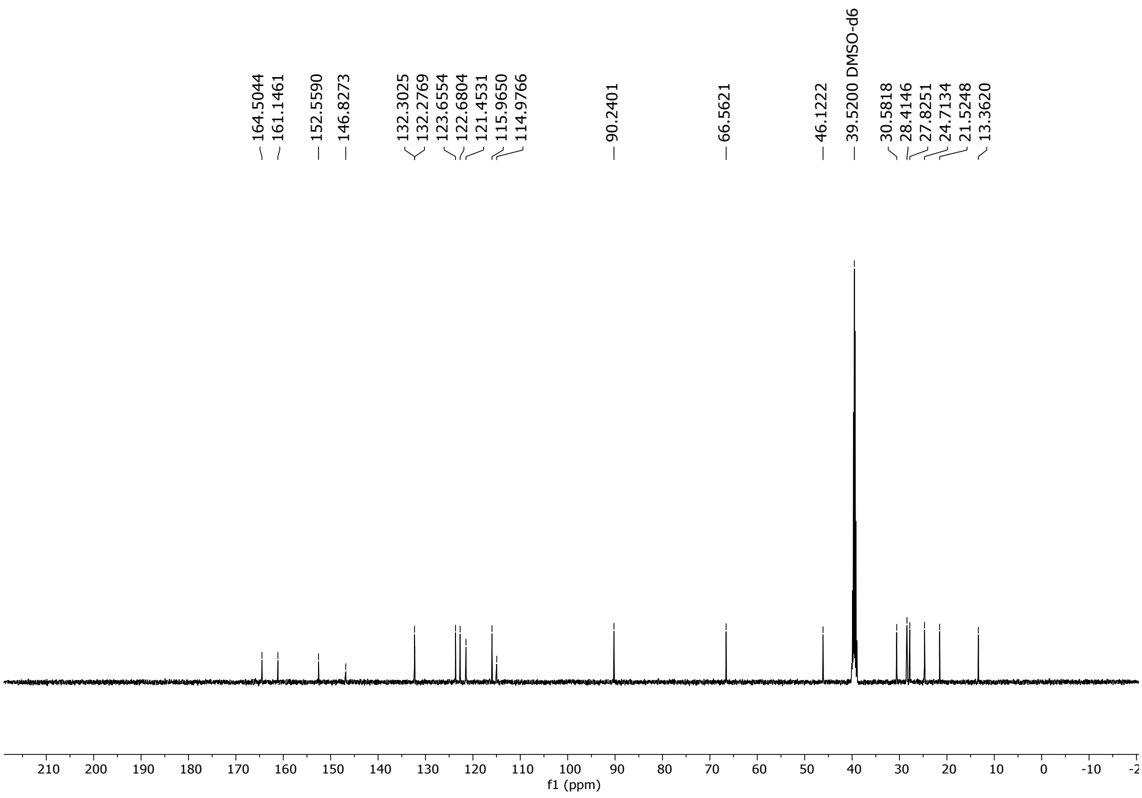

Figure S35. ^{13}C NMR spectrum (125.00 MHz, DMSO-d$_6$) of 4-(3-(4-hexyl-1*H*-1,2,3-triazol-1-yl)propoxy)-2*H*-chromen-2-one (**7l**)

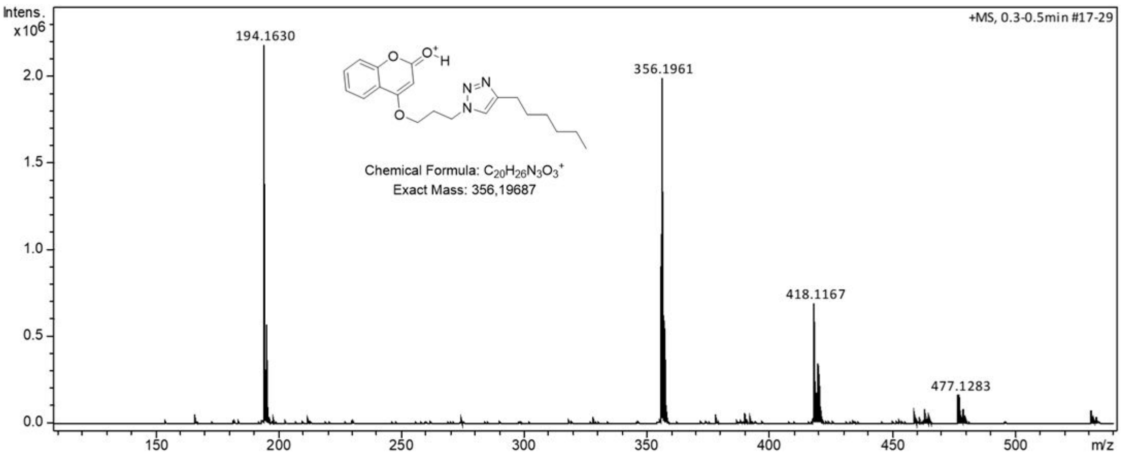

Figure S36. HRMS spectrum of 4-(3-(4-hexyl-1*H*-1,2,3-triazol-1-yl)propoxy)-2*H*-chromen-2-one (**7l**)

Biological

Gross necropsy and morbidity

Gross necropsy of the main organs was performed. As an indication of morbidity, the following signs were assessed: tremors; convulsion; salivation; diarrhea; lethargy; coma; pain signs;

increased rear arching, and mobility impairment. The necropsy included an examination of the external characteristics of the carcass; external body orifices; the abdominal, thoracic, and cranial cavities; organs/tissues of liver, thymus, right kidney, right testicle, heart, and lung.

Acute toxicity in mice

Animals upon euthanasia had their organs evaluated for signs of toxicity or morphological changes. No changes in any of the morbidity signs or mortality were observed in the treated groups.

Table S1. The monitoring of the animals indicated low toxicity for the coumarin-based hybrid **7f**. The results for the tested dose groups (control and treated) and the corresponding observations are presented: changes in body weight and food intake, signs of morbidity, possible mortality, and macroscopic alterations in collected organs. Each experimental group consisted of three C57BL/6 mice. The animals were monitored for 14 days, after which they were euthanized and necropsied.

**Treatament**	**Dose (mg/kg)**	**Change in body weight**	**Change in food consumption**	**Morbidity**	**Mortality**	**Gross Necropsy**
**Control**	0 N=3	Absent	Absent	Absent	Absent	Absent
**7f**	100 N=3	Absent	Absent	Absent	Absent	Absent
	200 N=3	Absent	Absent	Absent	Absent	Absent
	400 N=3	Absent	Absent	Absent	Absent	Absent

Target search through structural similarity

Structural similarity searches were performed based on the structure of the most promising compound (**7f**) in the Protein Data Bank, PubChem, ZINC15, and DrugBank databases. Targets were selected according to their structural similarity to these compounds, based on the Tanimoto coefficient, and on evidence suggesting these biological targets may be related to cancer. Similarity was considered significant when above 70%, which suggests that the compounds may share a molecular target. Additionally, searches were conducted in databases such as PubMed for scientific articles related to the identified targets.

Next, the structures of the targets were retrieved from the RCSB Protein Data Bank. The selected targets met the following criteria: the protein structure had to be from *Homo sapiens*, have the highest amino acid coverage, be complexed with a ligand, and have the lowest resolution. The obtained protein structures were protonated at pH 7.4 using the online PDB2PQR server with default PropKa settings and minimized using 2500 steps of steepest descent followed by 250 steps of conjugate gradient minimization in Swiss-SPDBViewer.

Molecular docking

All chemical structures of the docked compounds are shown in Supplementary Figure S38 for clarification.

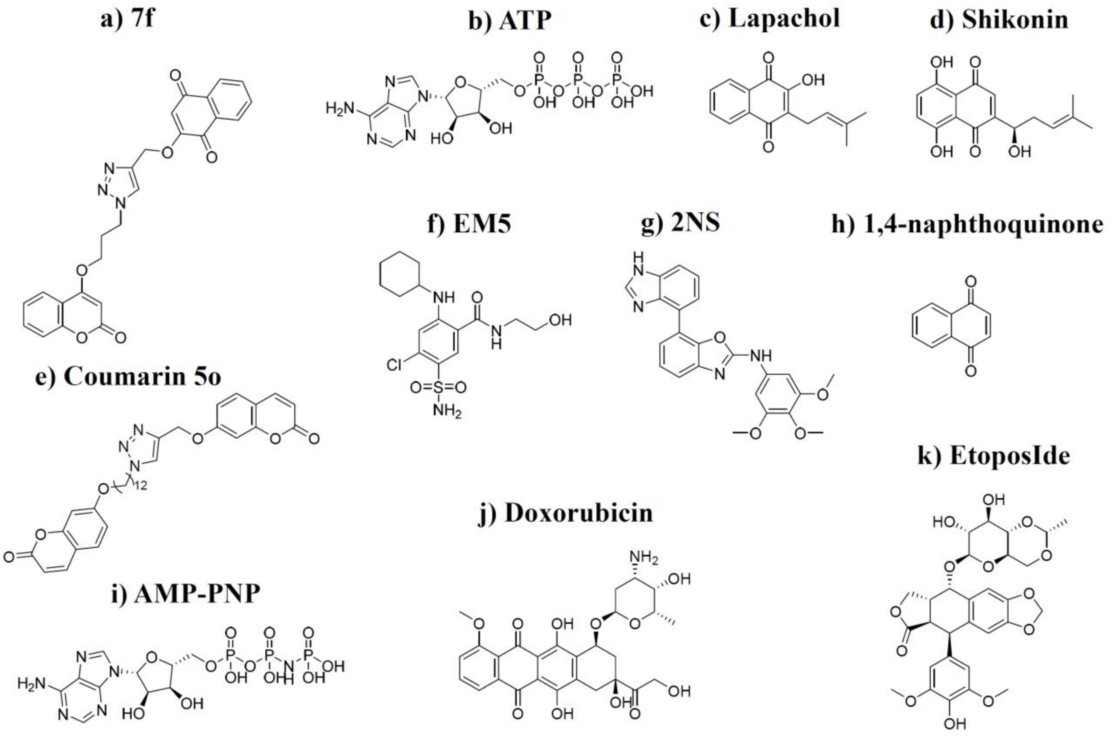

Supplementary Figure S37. Chemical structures of the compounds analyzed: (a) 7f; (b) ATP; (c) Lapachol; (d) Shikonin; (e) Coumarin 5o; (f) 4-chloranyl-2-(cyclohexylamino)-N-(2-hydroxyethyl)-5-sulfamoyl-benzamide (EM5); (g) 2-amino-7-substituted benzoxazole (2NS); (h) 1,4-naphthoquinone; (i) AMP-PNP; (j) Doxorubicin; and (k) Etoposide.

Molecular docking was performed using **7f** for molecular targets. Initially, the ligand was built, optimized and electrostatic charges were calculated using Spartan'10 software (Wavefunction Inc, CA, USA). A conformational analysis was performed using the MMFF force field, and the lowest-energy conformer was optimized using the semi-empirical PM3 method. Subsequently, an energy calculation was conducted using the Hartree-Fock method with the 6-31G* basis set (HF/6-31G*), and Mulliken charges were applied. The naphthoquinones shikonin,

lapachol and doxorubicin (DOX) were subjected to the same protocol to obtain their structures and were used for comparative purposes.*Targets search by similarities and structures retrieval*

Through literature review, naphthoquinone-targeted protein and their derivatives with antineoplastic activity were identified. Some of the selected targets for this study had been previously investigated as potential targets of naphthoquinones such as topoisomerases IIα and IIβ [1,2]. The complete set of three-dimensional structures of these proteins was retrieved from the Protein Data Bank (PDB) under the following codes: carbonic anhydrase XII (PDB 6G5L), ribosomal protein S6 kinase 2 (RSK2; PDB 4NW6), ATPase domain of topoisomerase IIα (PDB 1ZXM), DNA-binding domain of topoisomerase I (PDB 1K4T), topoisomerase IIα (PDB 5GWK), topoisomerase IIβ (PDB 3QX3). In the search for molecular targets based on the structural similarity with compounds from database repositories, only PubChem returned results with similarity above 70%, which was the threshold across all analyzed databases. When compared to **7f**, 81 results were obtained with high levels of similarity (>85%). Based on this analysis, carbonic anhydrase XII emerged as promising target for further molecular docking studies.

Binding with carbonic anhydrase XII (CAXII) was evaluated (Fig. S1). Carbonic anhydrases are metalloenzymes that coordinate a Zn^{2+} ion, which is essential for catalysis. Among the 16 isoenzymes, two—carbonic anhydrase IX and XII—are predominantly expressed in hypoxic tumors [3]. A similarity-based study revealed that coumarin 5o [3] shares over 85% structural similarity with **7f**. Although 5o is not a naphthoquinone, it contains coumarin and triazole moieties also present in **7f**, and was therefore chosen as a reference molecule, as it has demonstrated inhibitory activity against CAXII.

Despite the existence of known inhibitors for CA IX and XII, there are no reports of naphthoquinones acting as such. Compound **7f** maintained overlap in their coumarin and triazole moieties. When compared with compound 5o, their coumarin portions were also superimposed on those of the active reference compound. The co-crystallized ligand, 4-chloranyl-2-(cyclohexylamino)-N-(2-hydroxyethyl)-5-sulfamoyl-benzamide, also contains an aromatic ring, which overlapped with the coumarin portion of the compound. Kurt et al. (2019) suggested that the interaction of certain coumarins with residues Tyr6, Asn64, Lys69, Thr88, and Gln89 may account for their high affinity for this target.

Interestingly, **7f** formed interactions with these key residues, but notably through their naphthoquinone moieties. While compound 5o maintained weak van der Waals interactions, **7f** established hydrogen bonds with Asn64, Lys69, and Gln89. This could explain the stronger binding affinity observed for **7f** (-9.9 kcal·mol^{-1}), compared to the reference compound 5o (-7.8 kcal·mol^{-1}) and the redocking of the co-crystallized ligand (-8.9 kcal·mol^{-1}).

Taken together, the interaction profiles, binding energies, and visual inspection of docking poses suggest that CAXII is a promising molecular target for compound **7f**.

The binding of **7f** with RSK2 was also evaluated. RSK is a serine/threonine kinase that regulates several cellular processes such as proliferation, cell cycle progression, and apoptosis. RSK2 (ribosomal protein S6 kinase 2), one of its isoforms, translocates to the nucleus upon activation, where it phosphorylates substrates and controls actin cytoskeleton adhesion and rearrangement, thereby affecting motility in head and neck squamous cell carcinoma (HNSCC) [4,5]. Therefore, RSK2 emerges as an important therapeutic target in various types of cancer, particularly in head and neck cancer. As demonstrated by Zu et al. (2019), inhibition of this target mediates the cytotoxicity of lapachol in squamous cell carcinoma [5]. Hybrid **7f** in comparison with lapachol, did not show an overlap of their naphthoquinonic moieties. Lapachol retained its hydrogen bond interaction with Leu150, which may play a critical role in the binding of several RSK2 inhibitors [6–8] while **7f** exhibited van der Waals interaction with the same residue. However, lapachol showed lower binding affinity toward the RSK2 target compared to compound **7f** (-8.1 kcal·mol^{-1} and 10 kcal·mol^{-1}, respectively), possibly due to its molecular structure. However, despite their lower binding energy, **7f** appear to have been stabilized through intramolecular interactions between their rings due to their molecular flexibility, which may limit their ability to interact with the target. Consequently, the binding mode differed from that of lapachol, a known inhibitor. Our *in silico* results indicate that **7f** is unlikely to exert its effects primarily through this molecular target, although experimental validation would be required to confirm this observation.

Compound **7f** was subsequently evaluated against the DNA-binding domains of topoisomerases I, IIα, and IIβ. Doxorubicin, an anticancer drug widely used in the treatment of several malignancies, is known to inhibit DNA topoisomerases through intercalation with DNA, similarly to certain quinones [9–11]. Therefore, doxorubicin was selected for comparison with the docking poses obtained in these DNA-binding domains. **7f** intercalated into the nucleobases within the DNA bound to topoisomerases I, IIα, and IIβ.

The interaction of **7f** with the ATPase domain of topoisomerase IIα, in comparison with 1,4-naphthoquinone and the co-crystallized ligand AMP-PNP. Wei et al. (2005) demonstrated that the ribose ring of the co-crystallized ligand is stabilized through hydrogen bonds between the 2′ and 3′ hydroxyl groups and the side chains of residues Ser149 and Asn150 [12–14], although **7f** formed hydrogen bonds with residue Asn150, only van der Waals interactions were observed with residue Ser140. In addition, **7f** maintained van der Waals contacts with key active site residues such as Asn91 and Thr215. When compared to 1,4-naphthoquinone (-7.3 kcal·mol^{-1}) and 7f (-10,7 kcal·mol^{-1}) exhibited binding energies with higher affinity than 1,4-naphthoquinone, yet

lower than that of the co-crystallized ligand (-11.8 kcal·mol^{-1}). Although 1,4-naphthoquinone overlapped with compound **7f**, the naphthoquinone moiety did not occupy the same position within the active site. Instead, the coumarin portion overlapped the naphthoquinone of 1,4-naphthoquinone. Given the distinct binding mode observed for **7f** compared to the reference inhibitor, it is plausible that this protein may not represent its primary molecular target; however, further experimental studies are necessary to substantiate this inference.

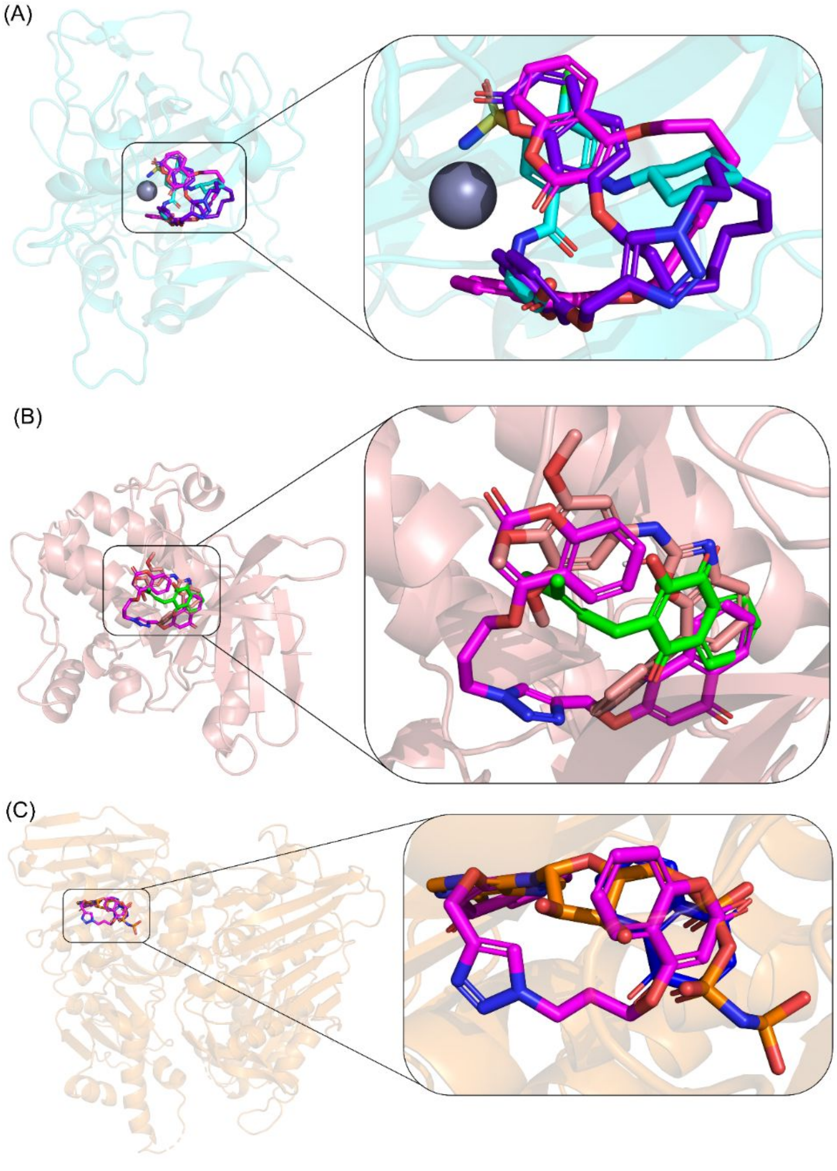

Figure S38. Binding mode of compounds (a) **7f** (magenta), the co-crystallized ligand (cyan), and the 5o compound (purple) to carbonic anhydrase XII. The gray sphere represents the metal ion Zn2+. (B) Binding mode of compounds **7f** (magenta), the co-crystallized ligand (pink), and the lapachol compound (green) to RSK2. (C) Binding mode of compounds **7f** (magenta), the co-

crystallized ligand (orange), and the 1,4-naphthoquinone compound (blue) to the ATPase domain of topoisomerase IIα.

Anthracyclines, such as doxorubicin, are classified as gold-standard anticancer therapies and are considered essential medicines by the World Health Organization [15]. In a recent review, Swedan et al. (2023) highlighted that the planar aglycone structure and polyaromatic system of anthracyclines are primarily responsible for their DNA intercalating properties. In docking studies with the DNA-binding domain of topoisomerase IIα, **7f** aligned with nucleobases DA12–DT9 and DG13–DC8, similarly to doxorubicin and the co-crystallized inhibitor etoposide. Notably, guanine 7 formed a hydrogen bond with the oxygen atom in the naphthoquinone moiety of the compounds.

Swedan et al. (2023) also emphasized the importance of the glycosyl moiety in anthracyclines, which projects into the helical groove, increasing interactions with the DNA-binding domain of topoisomerase II and stabilizing the drug–DNA–enzyme complex. As shown in Figure S38, the glycosyl moiety of doxorubicin lies in close proximity to the coumarin moiety of **7f**. This glycosyl group interacts via van der Waals forces with residues Met762, Met766, and the nucleobase DC11. **7f** establishes additional interactions, which likely contribute to its more favorable binding energy: −11.1 kcal·mol^{-1} for **7f**, compared to −10.3 kcal·mol^{-1} for co-crystallized etoposide and −10.2 kcal·mol^{-1} for doxorubicin.

The naphthoquinone moiety of **7f** partially overlaps with that of doxorubicin. Etoposide contains a 2,6-dimethoxyphenol group, recognized as a key structural feature for topoisomerase interaction [15], and this aromatic ring appears to align with the naphthoquinone ring of 7**f**, suggesting a potential similarity in their mechanism of action. Altogether, the DNA-binding domain of topoisomerase IIα emerges as a promising molecular target for **7f**.

Although **7f** shared several common interacting residues with topoisomerase IIα, its binding mode within the DNA-binding domain of topoisomerase IIβ appeared markedly different. Due to the higher flexibility of **7f**, a greater number of contacts were observed compared to reference drugs; however, these interactions were generally weaker, including van der Waals interactions with nucleobases DG10–DC11, without the expected intercalation into the DNA base pairs typical of inhibitors.

Compound **7f**, along with doxorubicin and the co-crystallized ligand etoposide, engaged nucleobases DC8–DG13 through hydrogen bonding (**7f**, and the co-crystallized ligand) and π-stacking and hydrogen bonding (**7f**). Nevertheless, as previously mentioned, maintaining a planar

ring structure that allows for DNA intercalation appears to be critical for establishing key interactions and for enzymatic inhibition—something that was not observed for **7f**.

Thus, the compound displayed a distinct binding mode compared to doxorubicin and etoposide, with only partial overlap between the naphthoquinone ring of **7f** and the 2,6-dimethoxyphenol ring of etoposide (Figure S39). Notably, **7f** exhibited a slightly more favorable binding energy (−11.7 kcal·mol^{-1}) than doxorubicin (−11.5 kcal·mol^{-1}). However, etoposide displayed the strongest binding affinity (−14.6 kcal·mol^{-1}).

Similar to the co-crystallized ligand and doxorubicin, **7f** aligned with nucleobases DA113–DT10 and DC112–TGP11 in the DNA-binding domain of topoisomerase I. The naphthoquinone moiety of **7f** formed hydrogen bonds with TGP11, while doxorubicin also established a hydrogen bond with DT10 via its quinone moiety. **7f** exhibited lower binding energies (−11.2 kcal·mol^{-1}) compared to the known inhibitor doxorubicin (−10.0 kcal/mol), and slightly higher than the co-crystallized ligand AMP-PNP (−11.3 kcal·mol^{-1}).

Although base pairing interactions were observed in these docking poses, the pronounced differences in orientation and binding mode relative to doxorubicin and AMP-PNP suggest that this protein is less likely to be the primary target of 7f, pending experimental confirmation.

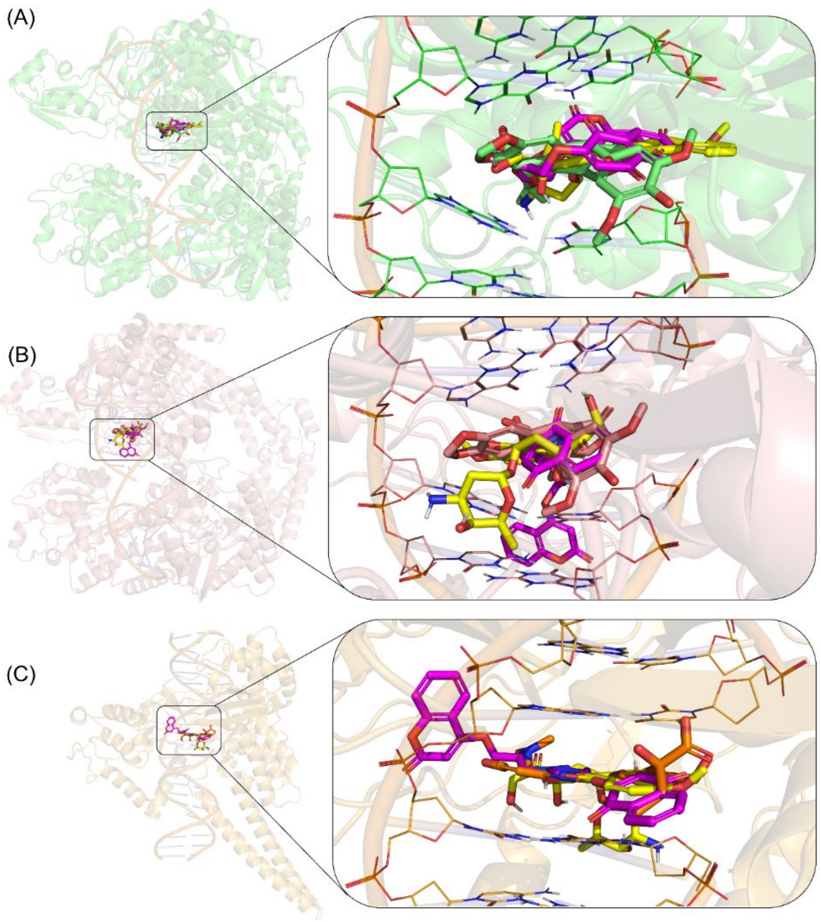

Figure S39. Binding mode of compounds (A) **7f** (magenta), the co-crystallized ligand (green), and the drug doxorubicin (yellow) to the DNA domain of topoisomerase IIα. (B) Binding mode of compounds **7f** (magenta), the co-crystallized ligand (pink), and the drug doxorubicin (yellow) to the DNA domain of topoisomerase IIβ. (C) Binding mode of compounds **7f** (magenta), the co-crystallized ligand (orange), and the drug doxorubicin (yellow) to the DNA domain of topoisomerase I.

Taken together, despite the high similarity between the DNA-binding domains of topoisomerase IIα and IIβ, the β isoform does not appear to be the molecular target of **7f**.

Supplementary Table S2. Raw data used for IC_{50} calculation shown in Figure 4A. Enzymatic activity of PKM2 in the presence of increasing concentrations of compound **7f** (0.8–100 µM), measured using an LDH-coupled assay. Activity values are expressed as percentage relative to the vehicle control (DMSO). Each concentration was tested in five independent experimental replicates. These data were used to generate the nonlinear regression curve and to determine the IC_{50} value presented in Figure 4A.

CONCENTRATION OF 7f		EXPERIMENTAL REPLICATES (% of activity relative to control)				
**µM**	**Log10(µM)**	**1**	**2**	**3**	**4**	**5**
100	2	42.38579	13.85542	46.12903	34.17367	45.14286
20	1.30103	68.0203	47.4026	85.71429	68.03279	87.41935
4	0.60206	96.22093	88.81988	107.0205	91.86352	99.18478
0.8	-0.09691	96.14325	81.35593	72.10777	97.25482	76.4557

Prediction of toxicity and pharmacokinetic properties.

The values of calculated octanol-water partition coefficient (cLogP), molecular weight (MW), number of hydrogen bond acceptors (nON), number of hydrogen bond donors (nOH/NH), and topological polar surface area (TPSA) were calculated using the SwissADME web server (http://www.swissadme.ch/)[16]. The most selective compound was analyzed, and carboplatin and doxorubicin were used as controls.

Physicochemical properties of 7f predicted favorable druglikeness parameters

In the context of developing new drug candidates, it is crucial to overcome toxic events caused by off-target interaction vital biomolecules. Throughout the years, extensive analysis of the physicochemical profiles of drugs and their interaction patterns has led to the development of web-based platforms designed to predict potential interactions of novel compounds prior to pre-

clinical or clinical testing, thereby facilitating the development of safer drugs [17]. Examples of such tools include SwissADME, which was employed in this study to assess compound **7f**. This platform provides a range of parameters to predict drug-likeness, relying on established rules that link physicochemical characteristics to potential adverse effects. One of the most widely applied guidelines is Lipinski's "Rule of Five" which classifies a compound as a poor candidate for oral administration if violates two of more of the following properties: (1) CLogP ≤ 5 (or MLogP, (2) hydrogen bond acceptors (nON) ≤ 10, (3) hydrogen bond donors (nOH/NH) ≤ 5, and (4) molecular weight (MW) ≤ 500 Da [18]. Additional frameworks, such as the "GSK 4/400 Rule", associates a CLogP > 4 and/or MW > 400 Da with poor ADMET outcomes across various molecule types, including acidic, basic and neutral compounds. These effects include increased plasma protein binding, decreased bioavailability, active efflux via phosphoglycoprotein-P, and hERG channel inhibition, that may lead to risk of cardiotoxicity [19]. In a complimentary manner, the "Pfizer Rule" was established based on animal studies involving 245 Pfizer's compounds developed over the years. A positive correlation was observed between compound toxicity and the combination of a CLogP > 3 and a TPSA < 75 Å, which increased the likelihood of a compound being classified as toxic by approximately 2.5-fold [20]. According to their study, compounds with low topological polar surface area (TPSA) values tend to exhibit broader tissue distribution, and when associated with high lipophilicity (CLogP), they may bind to various off-target proteins, thus increasing the potential for adverse effects. Furthermore, the work by Clark and coworkers emphasized the importance of TPSA in drug design, suggesting that values must be strictly adjusted between 60 and 140 Å allowing for efficient passive membrane permeability while minimizing excessive tissue penetration or poor absorption [21]. The physicochemical parameters of compound **7f** were obtained using the SwissADME server and are summarized in **Table 4**.

Table 4. Predicted safety profile and SwissADME-derived physicochemical parameters of **7f** in comparison with doxorubicin and carboplatin. Properties related to Lipinski's "Rule of Five" (CLogP, nON, nOH/NH and MW), the "Pfizer Rule" (CLogP and TPSA), "GSK Rule" (CLogP and MW) are shown. The compliance with each rule is indicated as Accepted or Non accepted.

**Compounds**	**Physicochemical Properties**							
	**CLogP**	**nON**	**nOH/NH**	**MW**	**TPSA ($Å^2$)**	**Lipinski's Rule Violation[a]**	**GSK Rule Violation[b]**	**Pfizer Rule Violation[c]**
**7f**	1.12	8	0	457.4	113.52	Accepted	Non Accepted	Accepted

**Doxorubicin**	-2.10	12	6	543.5	206.7	Non Accepted	Non Accepted	Accepted
**Carboplatin**	-1.79	6	4	371.2	126.64	Accepted	Accepted	Accepted

[a] Violations to the Lipinski "rule of 5": CLogP ≤ 5; MW, MW ≤ 500; nON, number of hydrogen bond acceptors ≤ 10; and nOH/NH, number of hydrogen bond donors ≤ 5.
[b] Violations to the "GSK Rule": CLogP > 4 and/or MW > 400.
[c] Violations to the "Pfizer Rule": CLogP > 3 and TPSA < 75 Å.

When compared to clinically used drugs such as doxorubicin and carboplatin, **7f** meets the criteria established by two of the three aforementioned drug-likeness rules with the only exception being the violation of the "GSK Rule" due to its molecular weight exceeding 400 Da. In contrast, doxorubicin violates two of the rules. These findings suggest that **7f**, exhibits a more favorable predicted drug-likeness profile and supports its potential as a safer anticancer candidate when compared to established chemotherapeutic agents.

References

[1] S. Kennedy, J.C. DiCesare, R.J. Sheaff, Topoisomerase I/II inhibition by a novel naphthoquinone containing a modified anthracycline ring system, Biochem Biophys Res Commun 408 (2011) 94–98. https://doi.org/10.1016/j.bbrc.2011.03.126.

[2] J.A. Collins, N. Osheroff, 1,2-Naphthoquinone as a Poison of Human Type II Topoisomerases, Chem Res Toxicol 34 (2021) 1082–1090. https://doi.org/10.1021/acs.chemrestox.0c00492.

[3] B.Z. Kurt, A. Dag, B. Doğan, S. Durdagi, A. Angeli, A. Nocentini, C.T. Supuran, F. Sonmez, Synthesis, biological activity and multiscale molecular modeling studies of bis-coumarins as selective carbonic anhydrase IX and XII inhibitors with effective cytotoxicity against hepatocellular carcinoma, Bioorg Chem 87 (2019) 838–850. https://doi.org/10.1016/j.bioorg.2019.03.003.

[4] W.S. Yang, M.J. Caliva, V.S. Khadka, M. Tiirikainen, M.L. Matter, Y. Deng, J.W. Ramos, RSK1 and RSK2 serine/threonine kinases regulate different transcription programs in cancer, Front Cell Dev Biol 10 (2023). https://doi.org/10.3389/fcell.2022.1015665.

[5] X. Zu, X. Xie, Y. Zhang, K. Liu, A.M. Bode, Z. Dong, D.J. Kim, Lapachol is a novel ribosomal protein S6 kinase 2 inhibitor that suppresses growth and induces intrinsic apoptosis in esophageal squamous cell carcinoma cells, Phytotherapy Research 33 (2019) 2337–2346. https://doi.org/10.1002/ptr.6415.

[6] A. Costales, M. Mathur, S. Ramurthy, J. Lan, S. Subramanian, R. Jain, G. Atallah, L. Setti, M. Lindvall, B.A. Appleton, E. Ornelas, P. Feucht, B. Warne, L. Doyle, S.E. Basham, I. Aronchik, A.B. Jefferson, C.M. Shafer, 2-Amino-7-substituted benzoxazole analogs as potent RSK2 inhibitors, Bioorg Med Chem Lett 24 (2014) 1592–1596. https://doi.org/10.1016/j.bmcl.2014.01.058.

[7] I. Aronchik, B.A. Appleton, S.E. Basham, K. Crawford, M. Del Rosario, L. V. Doyle, W.F. Estacio, J. Lan, M.K. Lindvall, C.A. Luu, E. Ornelas, E. Venetsanakos, C.M.

Shafer, A.B. Jefferson, Novel Potent and Selective Inhibitors of p90 Ribosomal S6 Kinase Reveal the Heterogeneity of RSK Function in MAPK-Driven Cancers, Molecular Cancer Research 12 (2014) 803–812. https://doi.org/10.1158/1541-7786.MCR-13-0595.

[8] R. Jain, M. Mathur, J. Lan, A. Costales, G. Atallah, S. Ramurthy, S. Subramanian, L. Setti, P. Feucht, B. Warne, L. Doyle, S. Basham, A.B. Jefferson, M. Lindvall, B.A. Appleton, C.M. Shafer, Discovery of Potent and Selective RSK Inhibitors as Biological Probes, J Med Chem 58 (2015) 6766–6783. https://doi.org/10.1021/acs.jmedchem.5b00450.

[9] M. Kciuk, A. Gielecińska, S. Mujwar, D. Kołat, Ż. Kałuzińska-Kołat, I. Celik, R. Kontek, Doxorubicin—An Agent with Multiple Mechanisms of Anticancer Activity, Cells 12 (2023) 659. https://doi.org/10.3390/cells12040659.

[10] H. Taymaz-Nikerel, M.E. Karabekmez, S. Eraslan, B. Kırdar, Doxorubicin induces an extensive transcriptional and metabolic rewiring in yeast cells, Sci Rep 8 (2018) 13672. https://doi.org/10.1038/s41598-018-31939-9.

[11] J. Lee, M.-K. Choi, I.-S. Song, Recent Advances in Doxorubicin Formulation to Enhance Pharmacokinetics and Tumor Targeting, Pharmaceuticals 16 (2023) 802. https://doi.org/10.3390/ph16060802.

[12] H. Wei, A.J. Ruthenburg, S.K. Bechis, G.L. Verdine, Nucleotide-dependent Domain Movement in the ATPase Domain of a Human Type IIA DNA Topoisomerase, Journal of Biological Chemistry 280 (2005) 37041–37047. https://doi.org/10.1074/jbc.M506520200.

[13] D. Gurbani, V. Kukshal, J. Laubenthal, A. Kumar, A. Pandey, S. Tripathi, A. Arora, S.K. Jain, R. Ramachandran, D. Anderson, A. Dhawan, Mechanism of Inhibition of the ATPase Domain of Human Topoisomerase IIα by 1,4-Benzoquinone, 1,2-Naphthoquinone, 1,4-Naphthoquinone, and 9,10-Phenanthroquinone, Toxicological Sciences 126 (2012) 372–390. https://doi.org/10.1093/toxsci/kfr345.

[14] N. Boonyalai, P. Sittikul, N. Pradidphol, N. Kongkathip, Biophysical and molecular docking studies of naphthoquinone derivatives on the ATPase domain of human Topoisomerase II, Biomedicine & Pharmacotherapy 67 (2013) 122–128. https://doi.org/10.1016/j.biopha.2012.08.005.

[15] H.K. Swedan, A.E. Kassab, E.M. Gedawy, S.E. Elmeligie, Topoisomerase II inhibitors design: Early studies and new perspectives, Bioorg Chem 136 (2023) 106548. https://doi.org/10.1016/j.bioorg.2023.106548.

[16] A. Daina, O. Michielin, V. Zoete, SwissADME: a free web tool to evaluate pharmacokinetics, drug-likeness and medicinal chemistry friendliness of small molecules, Sci Rep 7 (2017) 42717. https://doi.org/10.1038/srep42717.

[17] S.Q. Pantaleão, P.O. Fernandes, J.E. Gonçalves, V.G. Maltarollo, K.M. Honorio, Recent Advances in the Prediction of Pharmacokinetics Properties in Drug Design Studies: A Review, ChemMedChem 17 (2022). https://doi.org/10.1002/cmdc.202100542.

[18] C.A. Lipinski, F. Lombardo, B.W. Dominy, P.J. Feeney, Experimental and computational approaches to estimate solubility and permeability in drug

discovery and development settings, Adv Drug Deliv Rev 46 (2001) 3–26. https://doi.org/10.1016/S0169-409X(00)00129-0.

[19] M.P. Gleeson, Generation of a Set of Simple, Interpretable ADMET Rules of Thumb, J Med Chem 51 (2008) 817–834. https://doi.org/10.1021/jm701122q.

[20] J.D. Hughes, J. Blagg, D.A. Price, S. Bailey, G.A. DeCrescenzo, R. V. Devraj, E. Ellsworth, Y.M. Fobian, M.E. Gibbs, R.W. Gilles, N. Greene, E. Huang, T. Krieger-Burke, J. Loesel, T. Wager, L. Whiteley, Y. Zhang, Physiochemical drug properties associated with in vivo toxicological outcomes, Bioorg Med Chem Lett 18 (2008) 4872–4875. https://doi.org/10.1016/j.bmcl.2008.07.071.

[21] D.E. Clark, Rapid calculation of polar molecular surface area and its application to the prediction of transport phenomena. 1. Prediction of intestinal absorption, J Pharm Sci 88 (1999) 807–814. https://doi.org/10.1021/js9804011.
